# Multi-Substituted Quinolines as HIV-1 Integrase Allosteric Inhibitors

**DOI:** 10.3390/v14071466

**Published:** 2022-07-02

**Authors:** Long Phi Dinh, Jian Sun, Courtney D. Glenn, Krunal Patel, Julie A. Pigza, Matthew G. Donahue, Larry Yet, Jacques J. Kessl

**Affiliations:** 1 Department of Chemistry, University of South Alabama, Mobile, AL 36688, USA; dinh.66@buckeyemail.osu.edu (L.P.D.); cdg1721@jagmail.southalabama.edu (C.D.G.); 2 Department of Chemistry and Biochemistry, University of Southern Mississippi, Hattiesburg, MS 39406, USA; jian.sun@usm.edu (J.S.); krunal.patel@usm.edu (K.P.); julie.pigza@usm.edu (J.A.P.); matthew.donahue@usm.edu (M.G.D.)

**Keywords:** HIV-1, integrase, ALLINI, quinolines, multimerization

## Abstract

Allosteric HIV-1 integrase (IN) inhibitors, or ALLINIs, are a new class of antiviral agents that bind at the dimer interface of the IN, away from the enzymatic catalytic site and block viral replication by triggering an aberrant multimerization of the viral enzyme. To further our understanding of the important binding features of multi-substituted quinoline-based ALLINIs, we have examined the IN multimerization and antiviral properties of substitution patterns at the 6 or 8 position. We found that the binding properties of these ALLINIs are negatively impacted by the presence of bulky substitutions at these positions. In addition, we have observed that the addition of bromine at either the 6 (6-bromo) or 8 (8-bromo) position conferred better antiviral properties. Finally, we found a significant loss of potency with the 6-bromo when tested with the ALLINI-resistant IN A128T mutant virus, while the 8-bromo analog retained full effectiveness.

## 1. Introduction

The Integrase (IN) enzyme of the Human Immunodeficiency Virus type 1 (HIV-1) is required for viral replication. The catalytic activity of IN plays a major role during the early stage of the virus life cycle, as it is responsible for the integration of the viral DNA (vDNA) into the host chromatin. This vDNA integration activity has been developed as a therapeutic target, and four FDA-approved inhibitors (Raltegravir, Elvitegravir, Dolutegravir, and Bictegravir) [1] that bind to the IN catalytic site are currently used clinically to treat HIV-1-infected patients. Although these treatments are very effective, resistant strains have emerged against several of these drugs due to the high mutation rates of the virus [2]. Thus, the development of these drugs escaping mutations within the active site of IN underlines the importance of pursuing alternative inhibition sites on the enzyme.

The HIV-1 IN is structured into three distinct domains: the N-terminal domain (NTD), the catalytic core domain (CCD), and the C-terminal domain (CTD) [3,4]. During the integration process, these three IN domains work together to form a stable tetramer, or higher-order multimer (i.e., dodecamer), where vDNA and host chromosomal DNA are bound to the viral enzyme [5,6,7,8]. The integration of the HIV-1 genome also involves the interaction between IN and the cellular chromatin-associated protein LEDGF/p75, which bridges the IN-vDNA complex to active genes [9,10,11,12,13,14]. The LEDGF/p75 interacts with the stable IN tetramer through its C-terminal integrase binding domain (IBD) by inserting a small loop into a v-shaped pocket located at the IN CCD dimer interface [12,15].

**Figure 1:**
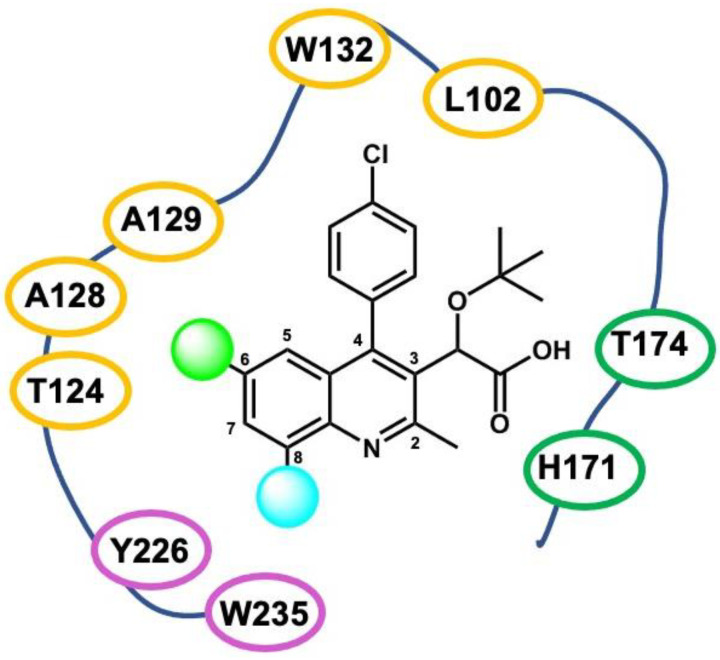
Representation of the quinoline scaffold within the integrase binding domain (IBD) pocket. The pocket space is defined with the green, magenta, and yellow segments which denote the individual monomeric units of the integrase multimer.

This binding pocket (Figure 1) has become of significant interest as an IN alternative target option. Allosteric IN inhibitors (ALLINIs) [16,17,18,19,20], which are also known as LEDGINs (LEDGF/p75 Inhibitors) [21], NCINIs (Noncatalytic Site Integrase Inhibitors) [22], or INLAIs (IN-LEDGF Allosteric Inhibitors) [23], selectively bind at the IBD binding pocket, away from the IN catalytic site and potently inhibit HIV-1 replication in cell culture. Importantly, these compounds are able to retain full potency against clinical strains resistant to the FDA-approved IN catalytic inhibitors [21].

While originally designed to block IN–LEDGF/p75 interactions, these ALLINIs have been shown to also impact IN functions by triggering an aberrant multimerization of the viral enzyme [16,22,24]. Additionally, several studies on the antiviral mode of action of ALLINIs have revealed that these compounds not only inhibit integration but also target the late stages of HIV-1 replication by yielding non-infectious virions. Electronic microscopy observations of the morphology of these mature virions have revealed striking drug-induced defects, where the viral ribonucleoprotein complex (RNP) is mis-localized to an eccentric position between the empty capsid (CA) core and the virion’s matrix layer, instead of being within the CA core [25]. These discoveries strongly suggested that IN not only catalyzes vDNA integration but also contributes to the maturation steps of the replication cycle. Using several in vitro and ex vivo approaches, we have previously reported a strong and essential interaction between viral RNA and IN within the virion [26]. Thus, these observations have led us and others to propose that IN plays a critical non-catalytic role during viral maturation that can be uniquely targeted using ALLINIs [26].

Through molecular modeling [27], crystallography [28], and biochemical experiments [29,30], it has been suggested that in addition to binding at the IN CCD dimer interface (Figure 1, green and yellow IN dimer), some ALLINIs have molecular extensions allowing them to bridge with the CTD of a third IN subunit (Figure 1, magenta IN monomer), an important pharmacological feature for the development of the antiviral potency of this class of inhibitors. We have previously derivatized position 7 of our quinoline-based ALLINI scaffold (quinoline scaffold position numbers are indicated in Figure 1) and observed that the IN’s multimerization properties are enhanced by optimizing hydrophobic interactions between the compound and the CTD (Figure 1, magenta IN monomer) [31]. These features not only improved the overall anti-viral potencies of these compounds but also significantly shifted the ALLINIs selectivity toward the viral maturation stage. In the present study, we have performed a sequential derivatization at both the 6 and 8 positions and tested the synthetized compounds through IN multimerization and antiviral assays. We found that while the potencies of these ALLINIs are negatively impacted by the presence of bulky substitutions, the addition of bromine at either position 6 or 8 conferred better antiviral properties against viral constructs.

## 2. Materials and Methods

*Synthesis of ALLINIs.* The experimental details of the organic synthesis and chemical characterization of the multi-substituted quinolines series are described in the supporting information (Supplementary File S1).

*Recombinant Proteins and IN Multimerization Assay.* The recombinant proteins 6xHis-tagged and FLAG-tagged full-length INs were expressed in *E. coli* and purified by column chromatography, as previously described [16]. The HTRF-based assay used to monitor inhibitor-induced aberrant multimerization of IN was performed as previously reported [32]. Briefly, two separate preparations of His-tagged and FLAG-tagged IN proteins were mixed in the presence of an increasing concentration of the test compounds and incubated for 2.5 h at room temperature. Anti-His6-XL665 and anti-FLAG-EuCryptate antibodies (Cisbio, Inc., Bedford, MA, USA) were then added to the reaction and incubated at room temperature for 3 h. The IN multimerization HTRF signal was recorded using a PerkinElmer EnSpire multimode plate reader, and dose–response curves were fitted with a sigmoidal dose–response equation with Hill slope to determine the compound EC50 using Origin software.

*Antiviral Assay.* The A128T substitution was introduced into the IN-coding region of the pNL4-3 using site-directed mutagenesis and verified by sequencing. The late-stage EC_50_ values were determined in single replication cycle as described previously [18]. Briefly, HEK293T cells were transfected with pNL4-3 plasmids (WT or A128T IN mutant) to produce the respective viruses in the absence or presence of indicated concentrations of ALLINIs. The virus supernatants were collected 24 hrs after drug treatment, and p24 concentrations were determined by a HIV-1 Gag p24 ELISA (ZeptoMetrix) following the manufacturer’s protocol. The HeLa TZM-bl target cells were then infected with quantities of virions equivalent to 4 ng of the HIV-1 p24. The cells were cultured for 48 hrs and cell extracts were prepared using a reporter lysis buffer (Promega). Luciferase activity was determined using a commercially available kit (Promega). The dose–response curves were fitted with a sigmoidal dose–response equation with Hill slope to determine the compound IC_50_ using Origin software.

*Molecular modeling*. The computer-based binding simulations were carried out using the Biovia Discovery Studio 2021 software package (Dassault Systèmes Biovia Inc, San Diego, CA, USA) on an Alienware area 51 R2 workstation using the methodology previously described [31]. The PDB code 5HOT, a 4.4 Å X-ray structure of the original isoquinoline ligand, was modified with quinoline. A symmetry operation was applied to protein files to correct the placement of the CTD following the ligand-modification-induced changes. For the calculation of the relative free binding energy of the ligand with the A128T IN mutant, the Ala-128 residue of protein Chain B was mutated to Thr-128. The protein was prepped using the automated clean protein function within the preparation of a standard dynamic cascade to correct for following issues: non-standard naming, disordered regions, incomplete residues, the protonation state of ionizable residues based on their pKa, missing hydrogens, correct terminal residues, and to fix connectivity- and bond-related issues. The best fit harmonic restraint with a maximum force constant of 7.50 was applied to all HIV-1 IN residues within 4.5 Å of the ligand. A fixed constraint was applied to all other remaining residues and the protein backbone within 9.5 Å of the ligand. Additional following distance restraints with force constant of 10.0 were applied to ensure correct placement of ligand during standard dynamic cascade: the carboxylic acid group on C3 position to Glu-170 and Ala-169 backbone of CCD protein chain A, the methyl group on the C2 position, and the tertbutoxy group on the C3 position to the His-171 sidechain. A dihedral restraint was applied to the quinoline scaffold. All distant residues outside of the 9.5 Å shell were excluded from the calculation during the standard dynamic cascade to optimize simulation speed. The standard dynamic cascade calculations were performed in the absence of a water shell to optimize calculation time. Automated standard dynamic cascade runs were performed in five steps. The first two steps consisted of 1000 iterations of the Steepest Descent minimization with a 1.0 RMS gradient, followed by 2000 iterations of the conjugated gradient minimalization at a 0.1 RMS gradient. Subsequently, simulated heating runs were performed, from 800 to 298 K, with a simulation time of 5 ps. This was followed by equilibration with a 10 ps simulation time at 298 K. Finally, the production run was carried out with a 20 ps simulation time, with the Nose variable temperature production method applied. The time interval for every step was set to 0.5 fs/step with results saved at every step. A 9.5 Å atom-based cut-off for nonbonding interactions was used during the standard dynamic cascade calculations, with the dielectric constant set at 2.0. After the final round of molecular dynamics, the lowest energy conformation structure was selected for the binding energy calculations. The ligand was isolated and manually defined for the binding energy calculations. Using smart minimization consisting of 1000 iterations of the Steepest Descent method, followed by 2000 iterations of the conjugate gradient in succession, the selected conformation was minimized to a final convergence criterion of 0.001. The ligand relative free binding energy was determined using an integrated binding energy-calculation program. All the above steps were repeated for each ligand in the series with both the WT HIV-1 IN and the A128T HIV-1 IN mutant.

## 3. Results

**Figure 2:**
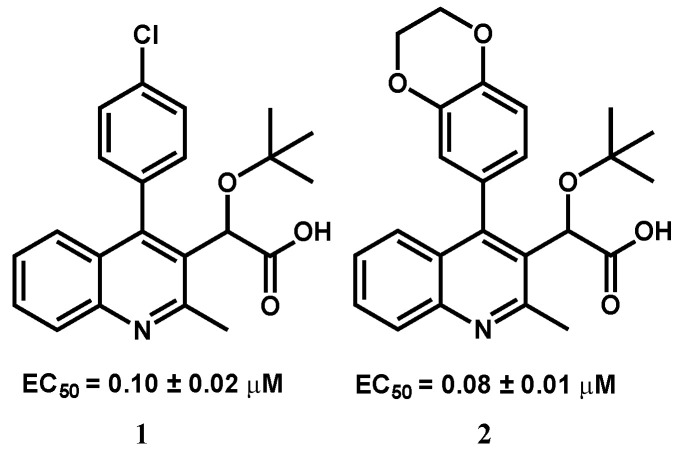
Quinolines utilized for HIV-1 IN inhibition whose EC_50_ values were previously evaluated utilizing the multimerization assay.

Potent ALLINIs share several common structural features including a central aromatic scaffold (quinoline, isoquinoline, pyrimidine, indole, or others) and a 3-α-*tert*-butoxy acetic acid side chain [16,17,21] (quinoline scaffold position numbers are indicated in Figure 1). This substitution on position 3 is ideal, as it provides several hydrogen bonds with the T174 and H171 residues that mimic the LEDGF/p75 IBD binding pattern [16,17,18]. Recent derivatization studies have focused on position 4 [20,33]. We and others have found that large aromatic substitutions at this site greatly enhance multimerization properties of the compounds by maximizing interaction with the hydrophobic pocket [20,33], including residues W132 and L102 (Figure 1). We found that a *para*-chloro-4-phenylquinoline substitution (Figure 1 and Figure 2) showed effective inhibition with an in vitro IN multimerization EC_50_ of 100 nM due to the stabilizing Cl–π interaction between the aromatic side chain of W132 and the chlorobenzene group [20]. Our most potent compound from this series was the 2,3-benzo [1,4] dioxanyl group at the 4-position of the quinoline (Figure 2), which showed an in vitro IN multimerization EC_50_ of 80 nM [20]. This observation led to the hypothesis that larger, multicyclic structures have the capability to rotate within the pocket, allowing for optimal interactions.

Examination of the available crystal structures of quinoline-based ALLINIs bound to the IN CCD [16,17] reveals a cluster of amino acids (T124, A128, and A129 in Figure 1) capable of providing additional stabilizing features to the IN–compound complex. Therefore, we initiated an investigation to probe for additional interactions in this area of the pocket. Compound **1** (Figure 2) was used as an initial scaffold to test a library of 6-substitued quinolines through systematic structure activity relationship studies using a previously described homogeneous time-resolved fluorescence (HTRF)-based IN multimerization in vitro assay known to accurately predict the antiviral potency for this class of inhibitors [16,32].

**Scheme 1:**
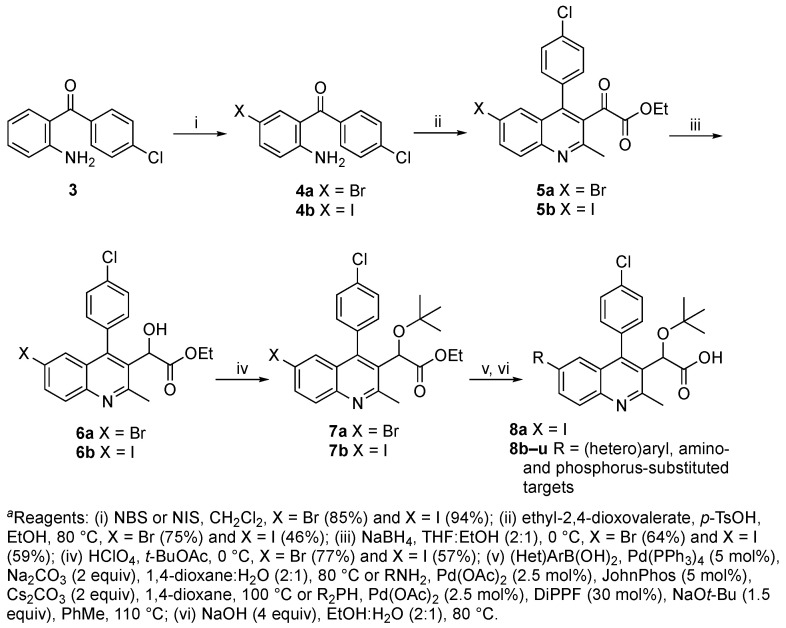
Synthesis of 6-Substituted-4-chlorophenylquinolines.

To probe the steric and electronic interactions within the T124-A128-A129 pocket residues, substitution at position 6 via Suzuki coupling was implemented as the key derivatizing step. Commercially available 2-amino-4′-chlorobenzophenone **3** was first brominated or iodinated at position 5 with *N*-bromosuccinimide (NBS) or with *N*-iodosuccinimide (NIS) in methylene chloride (Scheme 1) [34]. The afforded 2-amino-5-bromo (or iodo)-4′-chlorobenzophenone **4a/4b** was further reacted with ethyl-2,4-dioxovalerate with *p*-TsOH as a catalyst in a Friedlander quinoline reaction [35] to give the α-ketoquinoline esters **5a/5b** excellent purity. The obtained products **5a/5b** were reduced to α-hydroxy esters **6a/6b** with sodium borohydride in the THF:MeOH (2:1) [36]. The afforded ethyl 2-hydroxyacetate **6a/6b** were further reacted with *tert*-butyl acetate with perchloric acid as a catalyst to supply the desired *tert*-butoxyquinoline esters **7a/7b** [37]. These compounds served as common scaffold to react with a library of (hetero)arylboronic acids via Suzuki cross-coupling reactions using tetrakis(triphenylphosphine)palladium(0) in 1,4-dioxane/H_2_O (2:1) at 80 °C to attach the various (hetero)aryl groups at position 6 of the quinoline core or to undergo Buchwald–Hartwig aminations or phosphino additions. The products of the cross-coupling reactions were further saponified in sodium hydroxide at 80 °C to afford our targeted quinolines **8a–u** (Table 1 and Table 2).

With the quinoline multi-substitution methodology in hand, we implemented our study of position 6. The 4-chlorophenylquinoline (Compound **1**—Figure 2 and Table 1) was utilized as reference point for relative comparison. Substitution with bromine resulted in a previously reported compound (**ALLINI-2**) [17,33] showing a slight decrease in the measured EC_50_ value, implying increased ability to cause multimerization (Table 1). However, substitution with iodine (**8a**) or amino (**8b**) groups resulted in a two-fold decrease in the EC_50_ values (Table 1). In order to probe for additional interactions of our scaffold with alanine residues 128 and 129, we generated a small library of 4-chlorophenylquinolines **8c–l** with 6-subtitued phenyl groups (Table 2). We tested several factors including size restrictions and steric and aromatic interactions that may contribute significantly to the binding affinity. We found that contrary to the trend, we previously observed with position 4 [20], the addition of large aryl groups on position 6 negatively impacted the multimerization properties of the scaffold. We observed a further increase in the measured EC_50_ with *para*-substituted phenyl groups (compare compound 8c with compounds **8d** to **8g** in Table 2). To further explore the properties of this series, we synthesized and tested both the *meta* (8h) and the *ortho* (**8i**) variants of **8g** (Table 2) and found no significant difference. Additional increases in the substitution’s size on *ortho* rendered the scaffold fully inactive (**8j** and **8k**). Because of the negative impact measured with our 6-substituted phenyl group series, we also generated and tested several other groups (Table 2), such as 3-pyridinyl (**8m**), anilinyl (**8n**), diphenylphosphinyl (**8o**), non-aromatic 6-membered heterocycles (**8p** and **8q**), and 5-membered aromatic heterocycles (**8r-u**). Similar to our observation from the previous series, we found a significantly lower potency, or the complete loss of potency, caused by the added bulk.

**Scheme 2:**
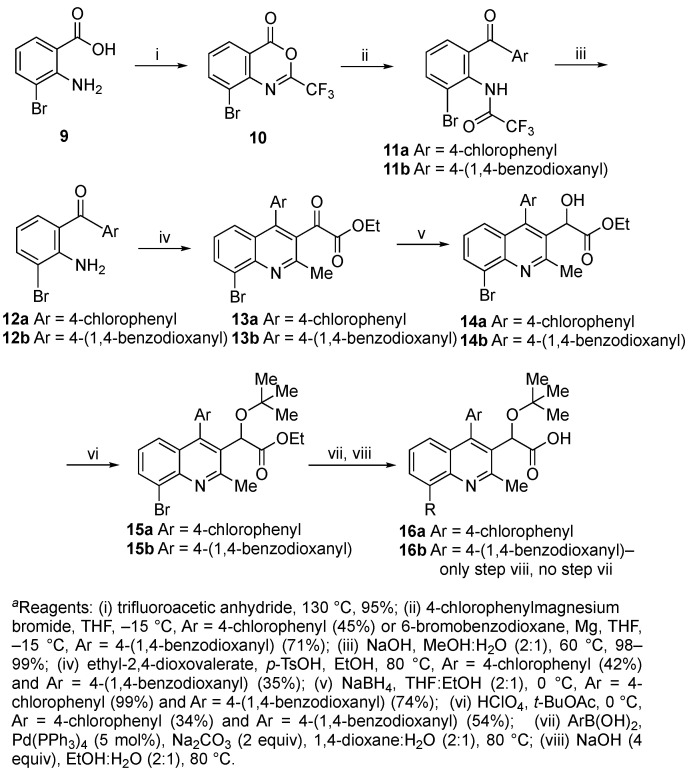
Synthesis of 4,8-Disubtituted Quinolines.

To probe for additional electronic interactions of the 4-chlorophenylquinoline scaffold with the Y226-W235 residues of the CTD, various substitutions at position 8 were also generated by using a similar synthetic route (Scheme 2). The oxazinone **10** was obtained by refluxing commercially available 3-bromoanthranilic acid (**9**) with four equivalents of trifluoroacetic acid anhydride. The oxazinone **10** was then reacted with 4-chlorophenylmagnesium bromide to give 4-chlorobenzophenone **11a**, which was hydrolyzed with sodium hydroxide in an MeOH/H_2_O (2:1) mixture to obtain the 2-aminobenzophenone product **12a** in excellent yields. As similarly described above (Scheme 1), **12a** was further reacted with ethyl-2,4-dioxovalerate with *p*-TsOH as a catalyst in a Friedlander quinoline reaction to yield **13a.** The afforded α-ketoquinoline ester **13a** was then reduced using sodium borohydride in THF:EtOH (2:1) to yield the α-hydroxy ester **14a,** which was further reacted with *tert*-butyl acetate in perchloric acid as catalyst to yield the *tert*-butoxy quinoline ester **15a**. This compound served as common precursor to react with a small library of arylboronic acids via Suzuki cross-coupling reactions using tetrakis(triphenylphosphine)palladium(0) in 1,4-dioxane/H_2_O (2:1) at 80 °C to attach various groups at position 8 of the quinoline core. The products of the cross-coupling reactions were further saponified in sodium hydroxide at 80 °C to obtain several 8-substituted-4-chlorophenylquinolines **16aa–ad** (Table 3).

Using the IN multimerization assay, we found that the addition of a bromine on position 8 (Table 3, **16aa**) slightly improved the observed in vitro EC_50_. With the objective of promoting further hydrophobic interactions between the compound and the Y226-W235 residues of the CTD, we tested a phenyl group addition (**16ab**) and *ortho*, *meta*, and *para* 8-methoxylpheny-substituted analogs **16ac–ae** (Table 3). We observed that the addition of these aryl groups on position 8 negatively impacted the multimerization properties of the scaffold.

As the 1,4-benzodioxanyl group on position 4 has been shown previously to slightly improve the compound inhibition, we adapted our synthetic route by using 1,4-benzodioxane-6-magnesium bromide on the oxazinone **10** to obtain the 4-(1 4-benzodioxanyl)benzophenone **11b** in similar yields, which was then hydrolyzed to benzophenone intermediate **12b.** The remainder of the route was kept unchanged and produced the *tert*-butoxyquinoline ester precursor **15b**, which was then saponified to deliver **16ba** (Table 4). The preparation of targets **17–19** is included separately in the Supporting Information section (Supplementary File S1). With this scaffold modification, we measured only minor and non-significant impact on the IN multimerization properties of the compound. Similar to the chlorophenyl series, a slight improvement was observed with the addition of a bromine at position 8 (Table 4, **16ba**).

Previous virological studies have identified the A128T substitution in HIV-1 IN as one of the primary mechanisms for resistance to several quinoline-based ALLINI compounds [17,21,22]. Indeed, a comparison of the crystal structures of the ALLINIs bound to wild-type (WT) and A128T IN constructs have revealed that the A to T substitution (see Figure 1) affects the positioning of the quinoline scaffold by exerting a negative steric effect to the compound [17].

Therefore, we have compared the impact of the bromine substitutions by measuring the antiviral properties on the WT and A128T viral constructs (Table 5). As quinoline-based ALLINIs are known to affect primarily the morphology of mature virions [23,25,31,38], we performed a dose–response effect on the late phase of replication. For this, HEK293T cells were transfected with replication-competent pNL4-3 (WT or A128T mutant) constructs in the presence of increasing concentrations of compounds, and allowed to produce virions. Twenty-four hours post-addition of the compounds, the virus-containing cell-free supernatant fractions were collected, and the amounts of viral particles produced were measured by p24 ELISA. As previously reported [17], the production of viral particles was not affected by the presence of ALLINIs. The infectivity of these progeny virions produced in presence of compounds was measured using a reporter cell line (TZM-bl) containing the HIV-1 LTR-luciferase reporter gene. For this, the TZM-bl target cells were infected by a constant amount of p24 equivalent virions (4 ng) with no further inhibitor being added. As previously described with several other ALLINIs [18,25,31], the progeny virions obtained in the presence of compounds displayed dose–response effects relative to infectivity (Table 5). We found that the addition of a bromine on position 6 (**17**) had a more positive effect than on position 8 (**16ba**) on the measured IC_50_ with the WT construct (Table 5). Interestingly, this difference was dramatically inverted with the IN A128T mutated construct.

**Figure 3:**
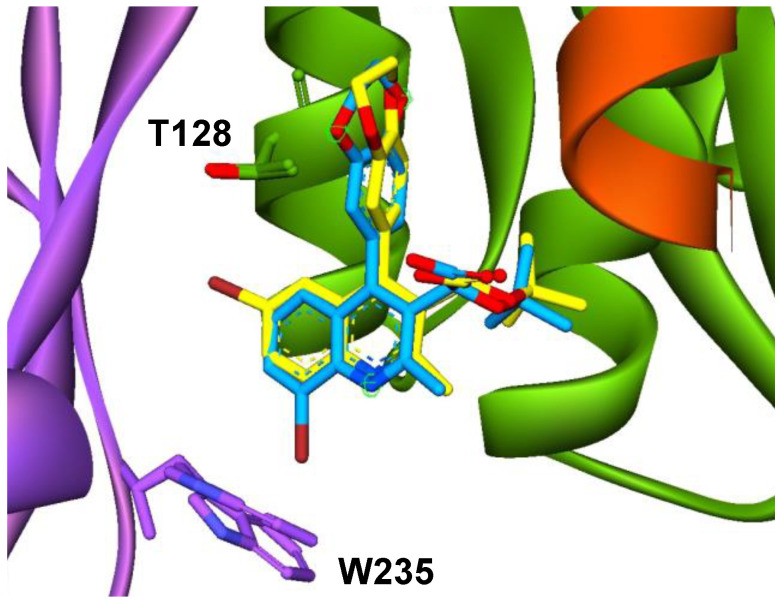
Overlay of the Binding Model of 17 (blue) vs. 16ba (yellow) in A128T.

**Figure 4:**
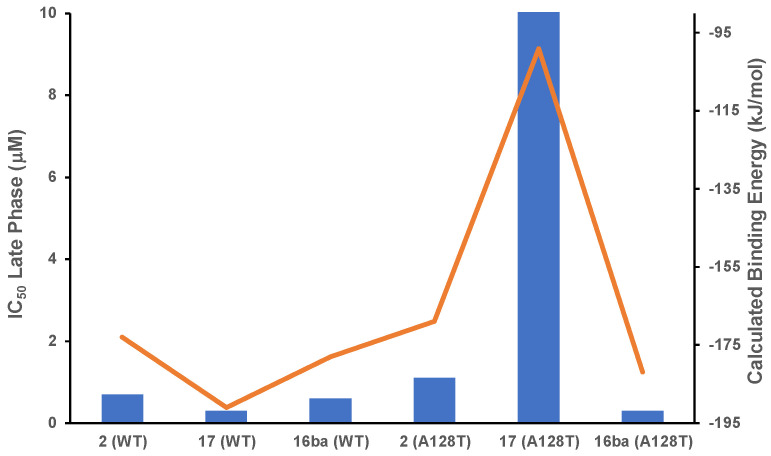
Antiviral activities and Calculated Binding Energy. The antiviral IC_50_ for each compound with the WT or IN A128T mutant is shown with blue bars (values extracted from Table 5). The orange line shows the calculated binding energy for each compound with the WT or IN A128T mutant.

To explain those observations, we performed a computational study of the binding of these compounds in the WT and mutated 3D structures. As a starting point, we used our previously calculated model of **2** bound to the WT IN tetramer [31] to generate new binding models for **17** and **16ba** with both the WT and the A128T constructs. It should be noted that while our compounds were synthesized as racemic mixtures, we chose to use the same stereoisomeric *S*-configuration for the 3- α-tert-butoxy acetic acid side chain for all our models. Using a CHARMM-based forcefield setting, a sequence of dynamic cascade runs that included minimization steps were applied to each structure with docked compounds, in which the lowest energy conformation was selected (details in Methods section). A representative display of the energy-minimized models of **17** and **16ba** bound to the A128T construct is shown on Figure 3. A close examination of the IN A128T mutated structure reveals that the T128 shift the whole scaffold slightly out of the binding pocket, as previously observed through crystallography with the chlorophenyl quinoline ALLINI-2 [17]. Interestingly, when no substitution is present on position 6, the compound is still slightly pushed, but the added bromine on position 8 in **16ba** provides a weak π–Br interaction [39] with the W235 of the CTD (Figure 3), sufficient to maintain binding. The calculated the binding energies of these compounds with both proteins was then extracted from each model and plotted against the experimentally measured antiviral IC_50_s (Figure 4). The calculated binding energies significantly correlated with the antiviral values as shown in Figure 4.

## 4. Discussion

Early optimization work on quinoline-based ALLINIs has shown that the tert-butoxy acetic acid side chain in position 3 (Figure 1) is ideal [16,17,21] as it provides several hydrogen bond interactions with the T174 and H171 residues. Several studies have also found that large aromatic substitutions at position 4 [20,33] greatly enhance the multimerization properties of the compounds by maximizing interaction with residues W132 and L102 (Figure 1). More recently [31], we have observed that a substitution in position 7 significantly enhances the compound properties through an increase in the hydrophobic interactions between the compound and the IN CTD. In the present study, to further explore additional features of the quinoline scaffold toward IN multimerization, we have kept both the tert-butoxy acetic acid side chain in position 3 and our previously optimized groups in position 4 constant [20] (Figure 2) to sequentially derivatize positions 6 and 8. We found that the addition of large bulky groups on these two positions negatively impacted the multimerization properties of the scaffold. Interestingly, adding a bromine at either 6 [17] or 8 slightly increases the antiviral potency of the quinoline-based ALLINIs. The selection of the resistance mutation IN A128T has been frequently observed with quinoline-based ALLINIs [17,21,22]. Previously, pyridine-based ALLINIs have been shown to overcome this resistance mutation by repositioning the scaffold away from the A128 IN residue while increasing contact points with the CCD dimer interface [18] (Figure 1, green and yellow IN monomers). Here, we have observed that the same effect can be achieved with quinoline-based ALLINIs by leaving position 6 unsubstituted and adding a bromine on 8 to optimize interaction with the CTD of the third subunit (Figure 1 and Figure 3, magenta IN monomer).

## 5. Conclusions

We have shown that the binding properties and antiviral activities of both the 4-chlorophenyl and 4-benzodioxane substituted quinoline-based ALLINIs are negatively impacted by the presence of bulky substitutions in positions 6 and 8 of the scaffold. In addition, we have observed that the addition of bromine at either the 6 or 8 position on 4-benzodioxane analogs conferred better antiviral properties against the WT construct. Interestingly, the quinoline-based ALLINI-resistant IN A128T viral construct was fully sensitive to the compound when the bromine was on position 8.

## Figures and Tables

**Table 1:** In vitro IN multimerization EC50 activities of-substituted-4-chloroquinolines. a Data from Jentsch N. et al. (2018). b Data from Feng L. et al. (2013).

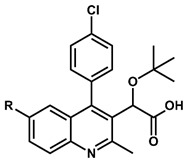		
**Compound**	**R**	**EC_50_ (µM)**
**1**	H	0.10 ±0.02 ^a^
**ALLINI-2**	Br	0.09 ±0.01 ^b^
**8a**	I	0.20 ±0.06
**8b**	NH_2_	0.19 ±0.05

**Table 2:** In vitro IN multimerization EC50 activities of 6-substituted-4-chlorophenyl quinolines. NA (non-active).

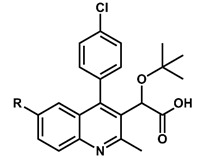					
**Compound**	**R**	**EC_50_ (µM)**	**Compound**	**R**	**EC_50_ (µM)**
**8c**	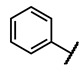	1.17 ± 0.14	**8m**	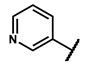	0.57 ± 0.29
**8d**	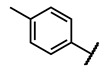	1.59 ± 0.04	**8n**	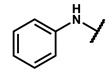	NA
**8e**	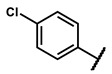	1.93 ± 0.01	**8o**	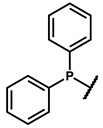	0.38 ± 0.01
**8f**	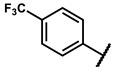	1.29 ± 0.01	**8p**	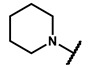	0.97 ± 0.40
**8g**	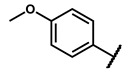	1.28 ± 0.01	**8q**	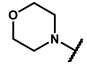	NA ^a^
**8h**	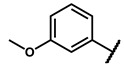	1.30 ± 0.37	**8r**	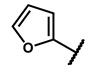	1.08 ± 0.17
**8i**	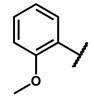	1.20 ± 0.14	**8s**	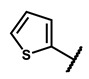	1.53 ± 0.13
**8j**	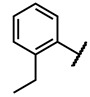	NA	**8t**	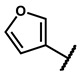	NA
**8k**	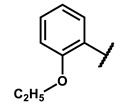	NA	**8u**	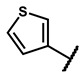	1.53 ± 0.33
**8l**	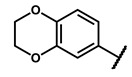	NA ^a^			

**Table 3:** In vitro IN multimerization EC50 activities of 8-substituted-4-chlorophenylquinolines.

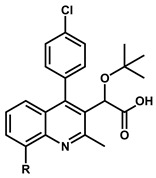		
**Compound**	**R**	**EC_50_ (µM)**
**16aa**	Br	0.09 ± 0.01
**16ab**	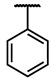	0.28 ± 0.03
**16ac**	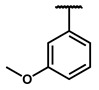	0.24 ± 0.01
**16ad**	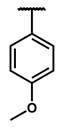	0.35 ± 0.08
**16ae**	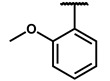	0.26 ± 0.04

**Table 4:** In vitro IN multimerization EC50 activities of 8-substituted-4-(1,2-benzendioxanyl) quinolines. a Data from Jentsch N. et al. (2018).

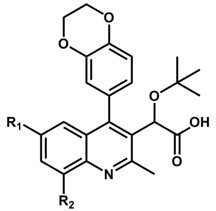			
**Compound**	**R_1_**	**R_2_**	**EC_50_ (µM)**
**2**	H	H	0.08 ± 0.01 ^a^
**17**	Br	H	0.10 ± 0.02
**18**	Me	H	0.09 ± 0.01
**16ba**	H	Br	0.05 ± 0.01
**19**	H	Cl	0.08 ± 0.01

**Table 5:** Antiviral activities IC50 of 6 or 8-substituted benzodioxanequinolines against the WT and A128T IN mutant.

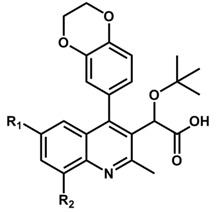					
**Compound**	**R_1_**	**R_2_**	**WT-IC_50_ (µM)**	**A128T-IC_50_ (µM)**
**2**	H	H	0.7 ± 0.1	1.1 ± 0.2
**17**	Br	H	0.3 ± 0.1	10.2 ± 2.5
**16ba**	H	Br	0.6 ± 0.1	0.3 ± 0.1

## Data Availability

All data are contained within the manuscript and the Supplementary Materials section.

## Supplementary Materials

The following supporting information can be downloaded at: https://www.mdpi.com/article/10.3390/v14071466/s1: Supplementary File S1: Organic synthesis.
