# Supplementary material for: Multi-Substituted Quinolines as HIV-1 Integrase Allosteric Inhibitors
Source: Viruses. 2022 Jul 2;14(7):1466. doi: 10.3390/v14071466 (PMC9324412; doi:10.3390/v14071466)
Supplement: Supplementary file 1 [file viruses-14-01466-s001.zip › viruses-1770254-supplementary.pdf]

**Multi-substituted Quinolines as HIV-1 Integrase Allosteric Inhibitors**

Long Phi Dinh[1], Jian Sun[2], Courtney Glenn[1], Krunal Patel[2], Julie A. Pigza[2], Matthew G. Donahue[2], Larry Yet[1*], Jacques J. Kessl[2*]

[1]*Department of Chemistry, University of South Alabama, Mobile, AL 36688*
[2]*Department of Chemistry and Biochemistry, University of Southern Mississippi, Hattiesburg, MS 39406*

## Supporting Information

**General Experimental Procedures** .......... 4
(2-Amino-5-bromophenyl) (4-chlorophenyl) methanone (**4a**) 4
(2-Amino-5-iodophenyl) (4-chlorophenyl) methanone (**4b**) 4
Ethyl 2-(6-bromo-4-(4-chlorophenyl)-2-methylquinolin-3-yl)-2-oxoacetate (5a) 5
Ethyl 2-(4-(4-chlorophenyl)-6-iodo-2-methylquinolin-3-yl)-2-oxoacetate (**5b**) 5
Ethyl 2-(6-bromo-4-(4-chlorophenyl)-2-methylquinolin-3-yl)-2-hydroxyacetate (**6a**) 5
Ethyl 2-(4-(4-chlorophenyl)-6-iodo-2-methylquinolin-3-yl)-2-hydroxyacetate (**6b**) 5
Ethyl 2-(6-bromo-4-(4-chlorophenyl)-2-methylquinolin-3-yl)-2-(*tert*-butoxy)acetate (**7a**) 6
Ethyl 2-(*tert*-butoxy)-2-(4-(4-chlorophenyl)-6-iodo-2-methylquinolin-3-yl)acetate (**7b**) 6
6-Bromo-2-(trifluoromethyl)-4*H*-benzo[*d*][1,3]oxazin-4-one 6
6-Iodo-2-(trifluoromethyl)-4*H*-benzo[*d*][1,3]oxazin-4-one 6
*N*-(4-Bromo-2-(2,3-dihydrobenzo[*b*][1,4]dioxine-6-carbonyl)phenyl)-2,2,2-trifluoroacetamide 7
*N*-(2-(2,3-Dihydrobenzo[*b*][1,4]dioxine-6-carbonyl)-4-iodophenyl)-2,2,2-trifluoroacetamide 7
(2-Amino-5-bromophenyl)(2,3-dihydrobenzo[*b*][1,4]dioxin-6-yl)methanone 7
(2-Amino-5-iodophenyl)(2,3-dihydrobenzo[*b*][1,4]dioxin-6-yl)methanone 7
Ethyl 2-(6-bromo-4-(2,3-dihydrobenzo[*b*][1,4]dioxin-6-yl)-2-methylquinolin-3-yl)-2-oxoacetate 8
Ethyl 2-(4-(2,3-dihydrobenzo[*b*][1,4]dioxin-6-yl)-6-iodo-2-methylquinolin-3-yl)-2-oxoacetate 8
Ethyl 2-(6-bromo-4-(2,3-dihydrobenzo[*b*][1,4]dioxin-6-yl)-2-methylquinolin-3-yl)-2-hydroxyacetate 8
Ethyl 2-(4-(2,3-dihydrobenzo[*b*][1,4]dioxin-6-yl)-6-iodo-2-methylquinolin-3-yl)-2-hydroxyacetate .......... 9
Ethyl 2-(6-bromo-4-(2,3-dihydrobenzo[*b*][1,4]dioxin-6-yl)-2-methylquinolin-3-yl)-2-(tert-butoxy)acetate .......... 9
Ethyl 2-(tert-butoxy)-2-(4-(2,3-dihydrobenzo[*b*][1,4]dioxin-6-yl)-6-iodo-2-methylquinolin-3-yl)acetate .......... 9
8-Bromo-2-(trifluoromethyl)-4*H*-benzo[*d*][1,3]oxazin-4-one (**10**) .......... 9
*N*-(2-Bromo-6-(4-chlorobenzoyl)phenyl)-2,2,2-trifluoroacetamide (**11a**) .......... 10
*N*-(2-Bromo-6-(2,3-dihydrobenzo[*b*][1,4]dioxine-6-carbonyl)phenyl)-2,2,2-trifluoroacetamide (**11b**) .......... 10
(2-Amino-3-bromophenyl)(4-chlorophenyl)methanone (**12a**) .......... 10
(2-Amino-3-bromophenyl)(2,3-dihydrobenzo[*b*][1,4]dioxin-6-yl)methanone (**12b**) .......... 10
Ethyl 2-(8-bromo-4-(4-chlorophenyl)-2-methylquinolin-3-yl)-2-oxoacetate (**13a**) .......... 11
Ethyl 2-(8-bromo-4-(2,3-dihydrobenzo[*b*][1,4]dioxin-6-yl)-2-methylquinolin-3-yl)-2-oxoacetate (**13b**) .......... 11
Ethyl 2-(8-bromo-4-(4-chlorophenyl)-2-methylquinolin-3-yl)-2-hydroxyacetate (**14a**) .......... 11
Ethyl 2-(8-bromo-4-(2,3-dihydrobenzo[*b*][1,4]dioxin-6-yl)-2-methylquinolin-3-yl)-2-hydroxyacetate (**14b**) .......... 12
Ethyl 2-(8-bromo-4-(4-chlorophenyl)-2-methylquinolin-3-yl)-2-(*tert*-butoxy)acetate (**15a**) .......... 12
Ethyl 2-(8-bromo-4-(2,3-dihydrobenzo[*b*][1,4]dioxin-6-yl)-2-methylquinolin-3-yl)-2-(*tert*-butoxy)acetate (**15b**) .......... 12
6-Methyl-2-(trifluoromethyl)-4*H*-benzo[*d*][1,3]oxazin-4-one .......... 12
*N*-(2-(2,3-Dihydrobenzo[*b*][1,4]dioxine-6-carbonyl)-4-methylphenyl)-2,2,2-trifluoroacetamide .......... 13
(2-Amino-5-methylphenyl)(2,3-dihydrobenzo[*b*][1,4]dioxin-6-yl)methanone .......... 13
Ethyl 2-(4-(2,3-dihydrobenzo[*b*][1,4]dioxin-6-yl)-2,6-dimethylquinolin-3-yl)-2-oxoacetate .......... 13
Ethyl 2-(4-(2,3-dihydrobenzo[*b*][1,4]dioxin-6-yl)-2,6-dimethylquinolin-3-yl)-2-hydroxyacetate .......... 13
Ethyl 2-(*tert*-butoxy)-2-(4-(2,3-dihydrobenzo[*b*][1,4]dioxin-6-yl)-2,6-dimethylquinolin-3-yl)acetate .......... 14
*N*-(2-(4-Chlorobenzoyl)-4-methylphenyl)-2,2,2-trifluoroacetamide .......... 14
8-Chloro-2-(trifluoromethyl)-4*H*-benzo[*d*][1,3]oxazin-4-one .......... 14

*N*-(2-Chloro-6-(2,3-dihydrobenzo[*b*][1,4]dioxine-6-carbonyl)phenyl)-2,2,2-trifluoroacetamide....................................14
(2-Amino-3-chlorophenyl)(2,3-dihydrobenzo[*b*][1,4]dioxin-6-yl)methanone ....................................................................15
Ethyl 2-(8-chloro-4-(2,3-dihydrobenzo[*b*][1,4]dioxin-6-yl)-2-methylquinolin-3-yl)-2-oxoacetate....................................15
Ethyl 2-(8-chloro-4-(2,3-dihydrobenzo[*b*][1,4]dioxin-6-yl)-2-methylquinolin-3-yl)-2-hydroxyacetate ...........................15
Ethyl 2-(*tert*-butoxy)-2-(8-chloro-4-(2,3-dihydrobenzo[*b*][1,4]dioxin-6-yl)-2-methylquinolin-3-yl)acetate ...................15

**General Procedure for the Preparation of 6- and 8- Aryl Quinolines** ............................................................................
Ethyl 2-(6-amino-4-(4-chlorophenyl)-2-methylquinolin-3-yl)-2-(*tert*-butoxy)acetate 16
Ethyl 2-(*tert*-butoxy)-2-(4-(4-chlorophenyl)-2-methyl-6-phenylquinolin-3-yl)acetate 16
Ethyl 2-(*tert*-butoxy)-2-(4-(4-chlorophenyl)-2-methyl-6-(*o*-tolyl)quinolin-3-yl)acetate 16
Ethyl 2-(4,6-bis(4-chlorophenyl)-2-methylquinolin-3-yl)-2-(*tert*-butoxy)acetate 17
Ethyl 2-(*tert*-butoxy)-2-(4-(4-chlorophenyl)-2-methyl-6-(4-(trifluoromethyl)phenyl)quinolin-3-yl)acetate 17
Ethyl 2-(*tert*-butoxy)-2-(4-(4-chlorophenyl)-6-(4-methoxyphenyl)-2-methylquinolin-3-yl)acetate 17
Ethyl 2-(*tert*-butoxy)-2-(4-(4-chlorophenyl)-6-(3-methoxyphenyl)-2-methylquinolin-3-yl)acetate 17
Ethyl 2-(*tert*-butoxy)-2-(4-(4-chlorophenyl)-6-(2-methoxyphenyl)-2-methylquinolin-3-yl)acetate 18
Ethyl 2-(*tert*-butoxy)-2-(4-(4-chlorophenyl)-6-(2-ethylphenyl)-2-methylquinolin-3-yl)acetate 18
Ethyl 2-(*tert*-butoxy)-2-(4-(4-chlorophenyl)-6-(2-ethoxyphenyl)-2-methylquinolin-3-yl)acetate 18
Ethyl 2-(*tert*-butoxy)-2-(4-(4-chlorophenyl)-2-methyl-6-(pyridin-3-yl)quinolin-3-yl)acetate 19
Ethyl 2-(*tert*-butoxy)-2-(4-(4-chlorophenyl)-2-methyl-6-(phenylamino)quinolin-3-yl)acetate 19
Ethyl 2-(tert-butoxy)-2-(4-(4-chlorophenyl)-6-(diphenylphosphaneyl)-2-methylquinolin-3-yl)acetate 19
Ethyl 2-(*tert*-butoxy)-2-(4-(4-chlorophenyl)-2-methyl-6-(piperidin-1-yl)quinolin-3-yl)acetate 19
Ethyl 2-(*tert*-butoxy)-2-(4-(4-chlorophenyl)-2-methyl-6-morpholinoquinolin-3-yl)acetate 20
Ethyl 2-(*tert*-butoxy)-2-(4-(4-chlorophenyl)-6-(furan-2-yl)-2-methylquinolin-3-yl)acetate 20
Ethyl 2-(*tert*-butoxy)-2-(4-(4-chlorophenyl)-2-methyl-6-(thiophen-2-yl)quinolin-3-yl)acetate 20
Ethyl 2-(*tert*-butoxy)-2-(4-(4-chlorophenyl)-6-(furan-3-yl)-2-methylquinolin-3-yl)acetate 21
Ethyl 2-(*tert*-butoxy)-2-(4-(4-chlorophenyl)-2-methyl-6-(thiophen-3-yl)quinolin-3-yl)acetate 21
Ethyl 2-(*tert*-butoxy)-2-(4-(4-chlorophenyl)-6-(2,3-dihydrobenzo[*b*][1,4]dioxin-6-yl)-2-methylquinolin-3-yl)acetate 21
Ethyl 2-(*tert*-butoxy)-2-(4-(4-chlorophenyl)-6-(4-fluorophenyl)-2-methylquinolin-3-yl)acetate 21
Ethyl 2-(*tert*-butoxy)-2-(4-(2,3-dihydrobenzo[*b*][1,4]dioxin-6-yl)-2-methyl-6-phenylquinolin-3-yl)acetate 22
Ethyl 2-(*tert*-butoxy)-2-(4-(2,3-dihydrobenzo[*b*][1,4]dioxin-6-yl)-6-(4-methoxyphenyl)-2-methylquinolin-3-yl)acetate22
Ethyl 2-(*tert*-butoxy)-2-(4-(4-chlorophenyl)-2-methyl-8-phenylquinolin-3-yl)acetate 22
Ethyl 2-(*tert*-butoxy)-2-(4-(4-chlorophenyl)-8-(2-methoxyphenyl)-2-methylquinolin-3-yl)acetate 23
Ethyl 2-(*tert*-butoxy)-2-(4-(4-chlorophenyl)-8-(3-methoxyphenyl)-2-methylquinolin-3-yl)acetate 23
Ethyl 2-(*tert*-butoxy)-2-(4-(4-chlorophenyl)-8-(4-methoxyphenyl)-2-methylquinolin-3-yl)acetate 23
Ethyl 2-(8-amino-4-(4-chlorophenyl)-2-methylquinolin-3-yl)-2-(*tert*-butoxy)acetate 24

**General Procedure for the Preparation of 6- and 8- Aryl Quinoline Acetic Acids**
2-(6-Bromo-4-(4-chlorophenyl)-2-methylquinolin-3-yl)-2-(*tert*-butoxy)acetic acid (**ALLINI-2**) 24
2-(*Tert*-butoxy)-2-(4-(4-chlorophenyl)-6-iodo-2-methylquinolin-3-yl)acetic acid (**8a**) 24
2-(6-Amino-4-(4-chlorophenyl)-2-methylquinolin-3-yl)-2-(*tert*-butoxy)acetic acid (**8b**) 24
2-(*Tert*-butoxy)-2-(4-(4-chlorophenyl)-2-methyl-6-phenylquinolin-3-yl)acetic acid (**8c**) 25
2-(*Tert*-butoxy)-2-(4-(4-chlorophenyl)-2-methyl-6-(*p*-tolyl)quinolin-3-yl)acetic acid (**8d**) 25
2-(4,6-Bis(4-chlorophenyl)-2-methylquinolin-3-yl)-2-(*tert*-butoxy)acetic acid (**8e**) 25
2-(*Tert*-butoxy)-2-(4-(4-chlorophenyl)-2-methyl-6-(4-(trifluoromethyl)phenyl)quinolin-3-yl)acetic acid (**8f**) 26
2-(*Tert*-butoxy)-2-(4-(4-chlorophenyl)-6-(4-methoxyphenyl)-2-methylquinolin-3-yl)acetic acid (**8g**) 26
2-(*Tert*-butoxy)-2-(4-(4-chlorophenyl)-6-(3-methoxyphenyl)-2-methylquinolin-3-yl)acetic acid (**8h**) 26
2-(*Tert*-butoxy)-2-(4-(4-chlorophenyl)-6-(2-methoxyphenyl)-2-methylquinolin-3-yl)acetic acid (**8i**) 27
2-(*Tert*-butoxy)-2-(4-(4-chlorophenyl)-6-(2-ethylphenyl)-2-methylquinolin-3-yl)acetic acid (**8j**) 27
2-(*Tert*-butoxy)-2-(4-(4-chlorophenyl)-6-(2-ethoxyphenyl)-2-methylquinolin-3-yl)acetic acid (**8k**) 27

2-(tert-butoxy)-2-(4-(4-chlorophenyl)-2-methyl-6-(pyridin-3-yl)quinolin-3-yl)acetic acid (**8m**) 28
2-(*Tert*-butoxy)-2-(4-(4-chlorophenyl)-2-methyl-6-(phenylamino)quinolin-3-yl)acetic acid (**8n**) 28
2-(*Tert*-butoxy)-2-(4-(4-chlorophenyl)-6-(diphenylphosphaneyl)-2-methylquinolin-3-yl)acetic acid (**8o**) 28
2-(*Tert*-butoxy)-2-(4-(4-chlorophenyl)-2-methyl-6-(piperidin-1-yl)quinolin-3-yl)acetic acid (**8p**) 28
2-(*Tert*-butoxy)-2-(4-(4-chlorophenyl)-2-methyl-6-morpholinoquinolin-3-yl)acetic acid (**8q**) 29
2-(*Tert*-butoxy)-2-(4-(4-chlorophenyl)-6-(furan-2-yl)-2-methylquinolin-3-yl)acetic acid (**8r**) 29
2-(*Tert*-butoxy)-2-(4-(4-chlorophenyl)-2-methyl-6-(thiophen-2-yl)quinolin-3-yl)acetic acid (**8s**) 29
2-(*Tert*-butoxy)-2-(4-(4-chlorophenyl)-6-(furan-3-yl)-2-methylquinolin-3-yl)acetic acid (**8t**) 30
2-(*Tert*-butoxy)-2-(4-(4-chlorophenyl)-2-methyl-6-(thiophen-3-yl)quinolin-3-yl)acetic acid (**8u**) 30
2-(*Tert*-butoxy)-2-(4-(4-chlorophenyl)-6-(2,3-dihydrobenzo[*b*][1,4]dioxin-6-yl)-2-methylquinolin-3-yl)acetic acid 30
2-(*Tert*-butoxy)-2-(4-(4-chlorophenyl)-6-(4-fluorophenyl)-2-methylquinolin-3-yl)acetic acid 30
2-(6-bromo-4-(2,3-dihydrobenzo[*b*][1,4]dioxin-6-yl)-2-methylquinolin-3-yl)-2-(tert-butoxy)acetic acid 31
2-(tert-butoxy)-2-(4-(2,3-dihydrobenzo[*b*][1,4]dioxin-6-yl)-6-iodo-2-methylquinolin-3-yl)acetic acid 31
2-(*Tert*-butoxy)-2-(4-(2,3-dihydrobenzo[*b*][1,4]dioxin-6-yl)-2-methyl-6-phenylquinolin-3-yl)acetic acid 31
2-(*Tert*-butoxy)-2-(4-(2,3-dihydrobenzo[*b*][1,4]dioxin-6-yl)-6-(4-methoxyphenyl)-2-methylquinolin-3-yl)acetic acid 32
2-(8-Bromo-4-(4-chlorophenyl)-2-methylquinolin-3-yl)-2-(*tert*-butoxy)acetic acid (**16aa**) 32
2-(*Tert*-butoxy)-2-(4-(4-chlorophenyl)-2-methyl-8-phenylquinolin-3-yl)acetic acid (**16ab**) 32
2-(*Tert*-butoxy)-2-(4-(4-chlorophenyl)-8-(3-methoxyphenyl)-2-methylquinolin-3-yl)acetic acid (**16ac**) 33
2-(*Tert*-butoxy)-2-(4-(4-chlorophenyl)-8-(4-methoxyphenyl)-2-methylquinolin-3-yl)acetic acid (**16ad**) 33
2-(*Tert*-butoxy)-2-(4-(4-chlorophenyl)-8-(2-methoxyphenyl)-2-methylquinolin-3-yl)acetic acid (**16ae**) 33
2-(8-Bromo-4-(2,3-dihydrobenzo[*b*][1,4]dioxin-6-yl)-2-methylquinolin-3-yl)-2-(tert-butoxy)acetic acid (**16ba**) 34
2-(*Tert*-butoxy)-2-(4-(2,3-dihydrobenzo[*b*][1,4]dioxin-6-yl)-2,6-dimethylquinolin-3-yl)acetic acid (**18**) 34
2-(*Tert*-butoxy)-2-(8-chloro-4-(2,3-dihydrobenzo[*b*][1,4]dioxin-6-yl)-2-methylquinolin-3-yl)acetic acid (**19**) 34

**General Experimental Procedures**

**Materials:** Common abbreviations.[1] Starting materials were purchased from commercial vendors and used without purification unless noted.[2]

**General Experimental Techniques:** Unless otherwise noted, all reactions were carried out using oven-dried glassware and standard syringe, cannula, and septa techniques, when necessary.[3] Tetrahydrofuran (THF), 1,4-dioxane were purchased from purchased as extra dry, anhydrous, AcroSeal 1-L glass bottle from Thermo Scientific. All boronic acids were purchased from either Combi-Blocks or Oakwood Chemicals. TLC analyses were performed on Whatman flexible aluminum backed TLC plates with a fluorescent indicator. Detection was conducted by UV absorption (254 nm). High-purity grade silica gel (Merck Grade 7734), pore size 60 Å, 70-230 mesh was used for all chromatographic separations. Flash column chromatography was performed on Biotage® SNAP KP-Sil or Sfär silica column. All chemicals used for synthetic procedures were reagent grade or better. Solutions were concentrated *in vacuo* with a rotary evaporator. All yields refer to isolated material that is chromatographically (TLC) and spectroscopically ($^1$H NMR) homogenous.

**NMR Characterization:** Chemical shifts were reported in parts per million (ppm, δ). All final compounds were demonstrated to have ≥90% purity. $^1$H NMR spectra were obtained on a JEOL 500 MHz spectrometer and were recorded in parts per millions from internal chloroform (7.26 ppm), dimethyl sulfoxide (2.54 ppm) on the δ scale. $^{13}$C NMR data were recorded on a JEOL 500 MHz NMR at 125 MHz spectrometer. NMR multiplicities were reported as follows: chemical shift [multiplicity (s=singlet, d=doublet, t=triplet, q=quartet, m=multiplet, br=broad signal), coupling constant(s) in hertz, integration, interpretation].

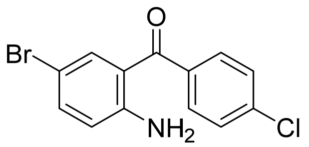

**(2-Amino-5-bromophenyl) (4-chlorophenyl) methanone (4a):** To a 250 mL round bottom flask was added (2-aminophenyl)-4′-chlorobenzophenone (500 mg, 2.16 mmol) in dry methylene chloride (10 mL) under nitrogen. The solution was cooled in an ice bath. *N*-Bromosuccinimide (384 mg, 2.16 mmol) was added portionwise. The reaction was allowed to stir overnight. Upon completion, the solution was quenched with saturated sodium thiosulfate (20 mL) and extracted with methylene chloride (3 x 50 mL). The organic layers were dried with sodium sulfate and condensed *in vacuo* to give a yellow solid (570 mg, 85%), which was used for the next step without further purification. $^1$H NMR (500 MHz, $CDCl_3$) δ 7.55 (d, *J* = 8.3 Hz, 2H), 7.46 (dd, *J* = 7.2, 2.1 Hz, 1H), 7.41 (d, *J* = 8.3 Hz, 2H), 7.39 (dd, *J* = 8.7, 2.0 Hz, 1H), 6.54 (d, *J* = 8.8 Hz, 1H), 6.08 (s, 2H).$^{13}$C NMR (126 MHz, $CDCl_3$) δ 196.6, 149.9, 138.0, 137.6, 137.2, 135.9, 130.7, 128.8, 119.1, 119.0, 106.8.

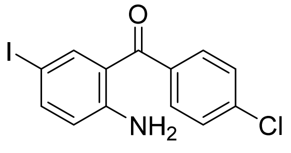

**(2-Amino-5-iodophenyl) (4-chlorophenyl) methanone (4b):** To a 250 mL round bottom flask was added (2-aminophenyl)-4′-chlorobenzophenone (200 mg, 0.864 mmol) on dry methylene chloride (10 mL) under nitrogen. The solution was cooled in an ice bath. *N*-Iodosuccinimide (194 mg, 0.864 mmol) was added portionwise. The reaction was allowed to stir overnight. Upon completion, the reaction mixture was quenched with saturated sodium thiosulfate (20 mL) and extracted with methylene chloride (3 x 50 mL). The organic layer were dried with sodium sulfate and condensed *in vacuo* to give a black red solid (290 mg, 94%). $^1$H NMR (500 MHz, $CDCl_3$) δ 7.65 (d, *J* = 2.1 Hz, 1H), 7.57 (d, *J* = 1.9 Hz,

---

[1] (a) For a general overview of organic chemistry acronyms see: Daub, G. H.; Leon, A. A.; Silverman, I. R.; Daub, G. W.; Walker, S. B. "The Use of Acronyms in Organic Chemistry." *Aldrichimica Acta* **1984**, 17(*1*), 13-23.

[2] For general purification procedures see: Armarego, W. L. F.; Perrin, D. D. "Purification of Laboratory Chemicals." 4th Ed. 1996, Butterworth-Heinemann.

[3] For general laboratory techniques see: (a) Furniss, B. S.; Hannaford, A. J.; Smith, P. W. G.; Tatchell, A. R. "Vogel's, Textbook of Practical Organic Chemistry." 5th Ed. **1989**, Longman. (b) "Handling Air-Sensitive Reagents" Aldrich Technical Bulletin AL-134, revised 12/94; (d) "Handling Pyrophoric Reagents" Aldrich Technical Bulletin, revised 06/95.

1H), 7.56 (d, *J* = 2.0 Hz, 1H), 7.50 (dd, *J* = 8.7, 2.1 Hz, 1H), 7.46 (d, *J* = 1.9 Hz, 1H), 7.44 (d, *J* = 1.9 Hz, 1H), 6.53 (d, *J* = 8.7 Hz, 1H), 6.08 (s, 3H). $^{13}$C NMR (126 MHz, $CDCl_3$) δ 196.5, 150.3, 142.6, 142.0, 138.0, 134.6, 130.7, 128.8, 119.4, 117.2, 75.2.

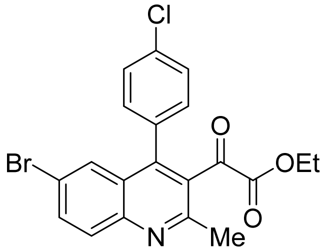


**Ethyl 2-(6-bromo-4-(4-chlorophenyl)-2-methylquinolin-3-yl)-2-oxoacetate (5a):** To 200 mL oven-dried heavy wall pressure vessel round bottom flask, (2-amino-5-bromophenyl) (4-chlorophenyl) methanone (9.39 g, 30.3 mmol), *p*-toluenesulfonic acid monohydrate (1.15 g, 6.06 mmol), ethyl-1,4-dioxovalerate (17.0 mL, 121.1 mmol) were dissolved in absolute ethanol (100 mL) and purged with argon gas. The flask was sealed and heated at 80 °C overnight. Upon completion, the solution was condensed in vacuo to give a thick orange oil. The crude product was purified by flash chromatography using ethyl acetate (0-30%) in petroleum ether to give a yellow solid (9.77 g, 75%). $^1$H NMR (500 MHz, $CDCl_3$) δ 7.97 (d, J = 8.9 Hz, 1H), 7.84 (dd, *J* = 8.9, 2.1 Hz, 1H), 7.70 (d, *J* = 2.2 Hz, 1H), 7.50 (d, *J* = 8.3 Hz, 2H), 7.24 (d, *J* = 8.3 Hz, 2H), 3.98 – 3.93 (m, 2H), 2.72 (s, 3H), 1.15 – 1.11 (m, 3H). $^{13}$C NMR (126 MHz, $CDCl_3$) δ 189.5, 161.6, 156.0, 146.9, 145.5, 136.2, 134.8, 132.2, 131.9, 131.0, 130.7, 130.2, 129.4, 128.8, 127.9, 125.9, 121.3, 63.0, 24.1, 13.8.

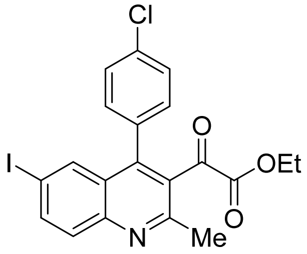


**Ethyl 2-(4-(4-chlorophenyl)-6-iodo-2-methylquinolin-3-yl)-2-oxoacetate (5b):** To a 50 mL oven-dried heavy wall pressure vessel round bottom flask, (2-Amino-5-iodophenyl) (4-chlorophenyl) methanone (500 mg, 1.40 mmol), *p*-toluenesulfonic acid monohydrate (53 mg, 0.28 mmol), ethyl-1,4-dioxovalerate (390 µl, 2.8 mmol) were dissolved in 200 proofs ethanol (10 mL) and purged with argon gas. The flask was sealed and heated at 80 °C overnight. Upon completion, the solution was condensed in vacuo to give a thick dark orange oil. The crude product was purified by flash chromatography using ethyl acetate (0-30%) in petroleum ether to give a yellow solid (308 mg, 46%). $^1$H NMR (500 MHz, $CDCl_3$) δ 7.95 (dd, *J* = 8.8, 1.9 Hz, 1H), 7.88 (dd, *J* = 1.9, 0.4 Hz, 1H), 7.77 (d, *J* = 8.9 Hz, 1H), 7.49 – 7.45 (m, 2H), 7.23 – 7.19 (m, 2H), 3.92 (q, *J* = 7.1 Hz, 2H), 2.68 (s, 3H), 1.09 (t, *J* = 7.2 Hz, 3H). $^{13}$C NMR (126 MHz, $CDCl_3$) δ 189.4, 161.6, 156.1, 147.2, 145.2, 140.0, 134.5, 132.2, 131.9, 130.9, 130.0, 129.3, 126.3, 93.0, 63.0, 24.2, 13.8.

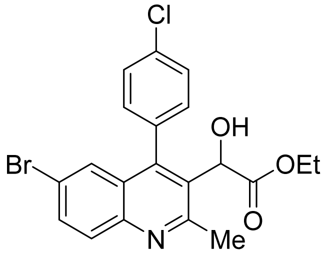


**Ethyl 2-(6-bromo-4-(4-chlorophenyl)-2-methylquinolin-3-yl)-2-hydroxyacetate (6a):** To a 100 mL Schlenk tube, ethyl 2-(6-bromo-4-(4-chlorophenyl)-2-methylquinolin-3-yl)-2-oxoacetate (9.77 g, 22.6 mmol), was dissolved in mixture of anhydrous THF and absolute ethanol (200 mL:100 mL). The flask was placed on an ice bath and purged with argon for 5 min. Sodium borohydride (855 mg, 22.6 mmol) was added portionwise. The flask was allowed to stir for 3 h under argon. Upon completion, the reaction was quenched with 1M HCl (100 mL) and extracted with ethyl acetate (4x150 mL). The organic layer was dried with anhydrous sodium sulfate and condensed in vacuo. The crude product was triturated with acetonitrile to yield a white powder (6.23 g, 64%). $^1$H NMR (500 MHz, DMSO-$d_6$) δ 7.90 (d, *J* = 8.9 Hz, 1H), 7.83 (dd, *J* = 8.9, 2.2 Hz, 1H), 7.68 – 7.58 (m, 2H), 7.41 – 7.33 (m, 1H), 7.36 – 7.28 (m, 1H), 7.30 – 7.23 (m, 1H), 6.22 (d, *J* = 4.5 Hz, 1H), 5.03 (d, *J* = 4.5 Hz, 1H), 4.13 – 3.94 (m, 2H), 2.64 (s, 3H), 1.07 (t, *J* = 7.1 Hz, 3H). $^{13}$C NMR (126 MHz, DMSO-$d_6$) δ 172.6, 159.5, 145.5, 145.3, 134.2, 134.2, 133.2, 132.4, 131.6, 131.3, 131.0, 129.3, 128.3, 127.5, 120.0, 69.4, 61.5, 24.2, 14.5.

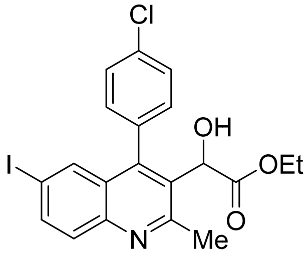


**Ethyl 2-(4-(4-chlorophenyl)-6-iodo-2-methylquinolin-3-yl)-2-hydroxyacetate (6b):**
To a 100 mL Schlenk tube, ethyl 2-(6-iodo-4-(4-chlorophenyl)-2-methylquinolin-3-yl)-2-oxoacetate (587 mg, 1.21 mmol), was dissolved in mixture of anhydrous THF and absolute ethanol (15 mL:3 mL). The flask was placed on an ice bath and purged with argon for 5 min. Sodium borohydride (46 mg, 1.21 mmol) was added portionwise. The flask was allowed to stir for 3 h under argon. The reaction was quenched with 1M HCl (10 mL) and extracted with ethyl acetate (3x40 mL). The organic layer was dried with anhydrous sodium sulfate and condensed *in vacuo*. The crude product was triturated with acetonitrile to yield white powder (344 mg, 59%). $^1$H NMR (500 MHz, $CDCl_3$) δ 7.88 (dd, *J* = 8.8, 1.9 Hz, 1H), 7.72 (d, *J* = 8.8 Hz, 1H), 7.60 (d, *J* = 1.9 Hz, 1H), 7.50 – 7.45 (m, 2H), 7.28 – 7.19 (m, 2H), 5.16 (s, 1H), 4.24 – 4.13 (m, 2H), 2.68 (s, 3H), 1.18 (t, *J* = 7.1 Hz, 3H). $^{13}$C NMR (126 MHz, $CDCl_3$) δ 173.5, 159.0, 146.8, 145.9, 138.7, 135.3, 133.8, 131.6, 130.7, 130.4, 129.2, 129.0, 128.5, 92.1, 69.7, 62.8, 24.0, 14.2.

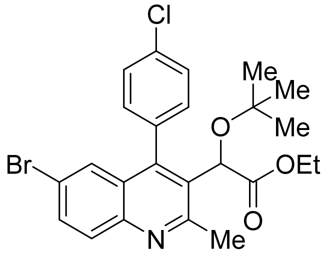

**Ethyl 2-(6-bromo-4-(4-chlorophenyl)-2-methylquinolin-3-yl)-2-(*tert*-butoxy)acetate (7a):** To a 500 mL tri-neck round bottomed flask, ethyl 2-(6-bromo-4-(4-chlorophenyl)-2-methylquinolin-3-yl)-2-hydroxyacetate (5.8 g, 13.4 mmol) was dissolved in *tert*-butyl acetate (100 mL). The vial was placed on an ice bath and purged with argon. Perchloric acid (4 mL) was added dropwise to the solution. The reaction vial was allowed to stir for 4 h. The reaction was neutralized with saturated sodium bicarbonate and extracted with ethyl acetate (3x150 mL). The combined organic layers were dried with anhydrous sodium sulfate and condensed *in vacuo.* Flash column chromatography was performed using hexane: ethyl acetate (4:1) to give a yellow oil (5.05 g, 77%). $^1$H NMR (500 MHz, $CDCl_3$) δ 7.90 (d, *J* = 8.9 Hz, 1H), 7.72 (dd, *J* = 8.9, 2.3 Hz, 1H), 7.55 – 7.52 (m, 2H), 7.44 – 7.41 (m, 1H), 7.40 (d, *J* = 2.2 Hz, 1H), 7.25 – 7.22 (m, 1H), 5.05 (s, 1H), 4.18 (m, 2H), 2.81 (s, 3H), 1.22 (t, *J* = 7.1 Hz, 3H), 0.98 (s, 9H). $^{13}$C NMR (126 MHz, $CDCl_3$) δ 172.3, 160.1, 145.5, 144.4, 135.1, 134.0, 132.9, 132.3, 131.1, 129.1, 128.7, 128.5, 127.5, 120.1, 76.3, 76.2, 70.8, 61.6, 28.1, 25.0, 14.2.

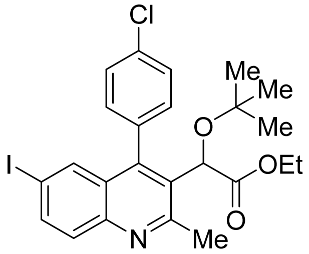

**Ethyl 2-(*tert*-butoxy)-2-(4-(4-chlorophenyl)-6-iodo-2-methylquinolin-3-yl)acetate (7b):** To a tall glass vial, ethyl 2-(4-(4-chlorophenyl)-6-iodo-2-methylquinolin-3-yl)-2-hydroxyacetate (236 mg, 0.499 mmol) was dissolved in *tert*-butyl acetate (10 mL). The vial was placed on an ice bath and purged with argon. Perchloric acid (1 mL) was added dropwise to the solution. The reaction vial was allowed to stir for 3 h. The reaction was neutralized with saturated sodium bicarbonate and extracted with ethyl acetate (3x20 mL). The combined organic layers were dried with anhydrous sodium sulfate and condensed *in vacuo.* Flash column chromatography was performed using hexane: ethyl acetate (4:1) to give a yellow oil (150 mg, 57%). $^1$H NMR (500 MHz, $CDCl_3$) δ 7.89 (ddd, *J* = 8.8, 2.1, 1.0 Hz, 1H), 7.75 (dd, *J* = 8.9, 0.9 Hz, 1H), 7.61 (dd, *J* = 2.0, 0.9 Hz, 1H), 7.55 – 7.52 (m, 2H), 7.42 (ddd, *J* = 8.4, 2.2, 1.1 Hz, 1H), 7.25 – 7.22 (m, 2H), 5.04 (d, *J* = 1.0 Hz, 1H), 4.21 – 4.13 (m, 2H), 2.80 (d, *J* = 0.9 Hz, 3H), 1.22 (td, *J* = 7.1, 1.0 Hz, 3H), 0.97 (d, *J* = 1.1 Hz, 9H). $^{13}$C NMR (126 MHz, $CDCl_3$) δ 172.2, 160.3, 145.8, 144.2, 138.2, 135.1, 134.0, 132.3, 131.1, 130.5, 129.1, 128.7, 128.0, 91.7, 76.3, 76.3, 70.7, 61.6, 28.1, 25.0, 14.2.

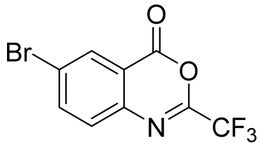

**6-Bromo-2-(trifluoromethyl)-4*H*-benzo[*d*][1,3]oxazin-4-one:** To a 50 mL oven-dried heavy wall pressure vessel round bottom flask, 5-bromoanthranilic acid (10.0 g, 46.3 mmol) was dissolved in trifluoroacetic anhydride (26.1 mL, 185 mmol). The flask was purged with argon gas and sealed. The reaction was heated up to 130 °C for 24 h. The reaction was quenched with saturated sodium bicarbonate solution to pH 8 and extracted with methylene chloride (3x200 mL). The combined organic layers were dried with anhydrous sodium sulfate and condensed *in vacuo* to yield brown powder (5.75 g, 52%). $^1$H NMR (500 MHz, $CDCl_3$) δ 8.38 (d, *J* = 2.2 Hz, 1H), 8.02 (dd, *J* = 8.6, 2.3 Hz, 1H), 7.65 (d, *J* = 8.6 Hz, 1H). $^{13}$C NMR (126 MHz, $CDCl_3$) δ 155.3, 147.7 (q, *J* = 42.5 Hz), 142.9, 140.7, 131.9, 30.0, 124.9, 119.3, 116.1 (q, *J* = 276.1 Hz). $^{19}$F NMR (471 MHz, $CDCl_3$) δ -72.3.

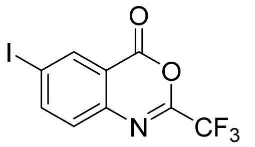

**6-Iodo-2-(trifluoromethyl)-4*H*-benzo[*d*][1,3]oxazin-4-one:** To a 50 mL oven-dried heavy wall pressure vessel round bottom flask, 5-iodoanthranilic acid (4.0 g, 15.2 mmol) was dissolved in trifluoroacetic anhydride (8.6 mL, 60.83 mmol). The flask was purged with argon gas and sealed. The reaction was heated up to 130 °C for 24 h. The reaction was quenched with saturated sodium bicarbonate solution to pH 8 and extracted with ethyl acetate (3x200 mL). The combined organic layers were dried with anhydrous sodium sulfate and condensed *in vacuo* to yield a brown powder (4.98 g, 96%). $^1$H NMR (500 MHz, DMSO-$d_6$) δ 8.31 (d, *J* = 2.2 Hz, 1H), 8.14 (d, *J* = 8.6 Hz, 1H), 7.77 (dd, *J* = 8.6, 2.2 Hz, 1H). $^{13}$C NMR (126 MHz, DMSO-$d_6$) δ 168.8, 154.6 (q, *J* = 36.4 Hz), 140.5, 140.0, 139.2, 126.2, 122.2, 120.3 – 112.7 (m), 88.8. $^{19}$F NMR (471 MHz, DMSO-$d_6$) δ -75.0.

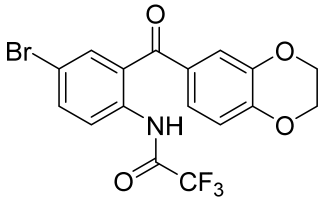

***N*-(4-Bromo-2-(2,3-dihydrobenzo[*b*][1,4]dioxine-6-carbonyl)phenyl)-2,2,2-trifluoroacetamide:** To a 200 mL Schlenk tube, 6-bromo-2-(trifluoromethyl)-4*H*-benzo[*d*][1,3]oxazin-4-one (5.75 g, 23.9 mmol) was dissolved in anhydrous THF (50 mL). The tube was purged with argon and placed in ice mixed with table salt to maintain the temperature below -10 °C. To a different tall oven-dried vial, 6-bromobenzodioxane (4.83 mL, 35.9 mmol) was dissolved in THF (30 mL) and purged with argon gas. Turned magnesium (919 mg, 38.3 mmol), and a few crystals of $I_2$ were added. The vial was placed in a warm water bath for 3 min then stirred at room temperature. The reaction was allowed to stir for an additional 10 min after the solution turned colorless. The solution then was loaded onto the syringe and added dropwise to the Schlenk tube. The reaction was allowed to stir for an additional 3 h. The reaction was quenched with 1M HCl (20 mL) and extracted with ethyl acetate (3x100 mL). The combined organic layers were dried with anhydrous sodium sulfate and condensed *in vacuo*. The crude solid was triturated with ice-cooled diethyl ether to yield faint yellow powder (5.45 g, 53%). $^1$H NMR (500 MHz, $CDCl_3$) δ 11.6 (N-H, s, 1H), 8.46 (d, *J* = 8.9 Hz, 1H), 7.78 (d, *J* = 2.4 Hz, 1H), 7.71 (dd, *J* = 8.9, 2.4 Hz, 1H), 7.30 (d, *J* = 2.1 Hz, 1H), 7.24 (ddd, *J* = 8.4, 2.1, 0.5 Hz, 1H), 6.95 (d, *J* = 8.5 Hz, 1H), 4.37 – 4.33 (m, 2H), 4.30 (ddd, *J* = 4.3, 3.4, 2.1 Hz, 2H). $^{13}$C NMR (126 MHz, $CDCl_3$) δ 196.3, 155.4 (q, *J* = 38.0 Hz), 148.8, 143.7, 136.8, 136.6, 135.7, 130.4, 126.2, 124.8, 123.4, 119.8, 117.5, 117.3, 119.2 – 112.1 (m), 64.9, 64.2. $^{19}$F NMR (471 MHz, $CDCl_3$) δ -76.0.

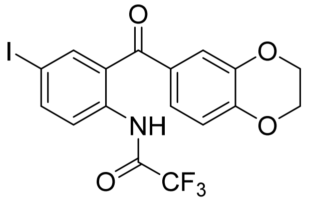

***N*-(2-(2,3-Dihydrobenzo[*b*][1,4]dioxine-6-carbonyl)-4-iodophenyl)-2,2,2-trifluoroacetamide:** To a 200 mL Schlenk tube, 6-iodo-2-(trifluoromethyl)-4*H*-benzo[*d*][1,3]oxazin-4-one (4.98 g, 14.6 mmol) was dissolved in anhydrous THF (45 mL). The tube was purged with argon and placed in ice mixed with table salt to maintain the temperature below -10 °C. To a different tall oven-dried vial, 6-bromobenzodioxane (2.95 mL, 21.9 mmol) was dissolved in THF (30 mL) and purged with argon gas. Turned magnesium (526 mg, 21.9 mmol), a few crystals of $I_2$ were added. The vial was placed in a warm water bath for 3 min then stirred at room temperature. The reaction was completed when the vial turned colorless. The solution then was loaded onto the syringe and added dropwise to the Schlenk tube. The reaction was allowed to stir for an additional 3 h. The reaction was quenched with 1M HCl (20 mL), water (100 mL), and extracted with ethyl acetate (3x100 mL). The combined organic layers were dried with anhydrous sodium sulfate and condensed *in vacuo*. The crude solid was triturated with ice-cooled diethyl ether to yield faint yellow power (5.24 g, 75%). $^1$H NMR (500 MHz, $CDCl_3$) δ 11.6 (N-H, s, 1H), 8.34 (dd, *J* = 8.7, 0.7 Hz, 1H), 7.96 (dd, *J* = 2.2, 0.8 Hz, 1H), 7.91 (dd, *J* = 8.8, 2.2 Hz, 1H), 7.32 (dd, *J* = 2.2, 0.7 Hz, 1H), 7.27 – 7.20 (m, 1H), 6.97 (dd, *J* = 8.4, 0.7 Hz, 1H), 4.38 – 4.30 (m, 4H). $^{13}$C NMR (126 MHz, $CDCl_3$) δ 196.2, 155.5 (q, *J* = 37.8 Hz), 148.8, 143.7, 142.7, 141.5, 137.2, 130.5, 126.4, 124.8, 123.6, 119.8, 117.5, 115.7 (m), 87.7, 64.9, 64.2. $^{19}$F NMR (471 MHz, $CDCl_3$) δ -76.0.

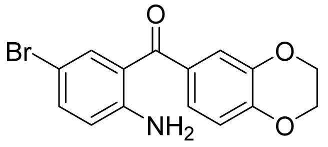

**(2-Amino-5-bromophenyl)(2,3-dihydrobenzo[*b*][1,4]dioxin-6-yl)methanone:** To a 200 mL heavy wall pressure vessel round bottom flask, *N*-(4-bromo-2-(2,3-dihydrobenzo[*b*][1,4]dioxine-6-carbonyl)phenyl)-2,2,2-trifluoroacetamide (3.68 g, 8.6 mmol), sodium hydroxide (2.06 g. 51.4 mmol) was dissolved in methanol (80 mL) and water (120 mL). The flask was capped and heated at 60 °C overnight. Upon completion, the solution was condensed *in vacuo* to a slurry solution. The solution was diluted with water and extracted with methylene chloride (3x100 mL). The combined organic layers were dried with anhydrous sodium sulfate and condensed *in vacuo* to yield a yellow solid (2.59 g, 91%). $^1$H NMR (500 MHz, $CDCl_3$) δ 7.56 (dd, *J* = 2.3, 0.6 Hz, 1H), 7.32 (ddd, *J* = 8.8, 2.5, 0.6 Hz, 1H), 7.23 (dd, *J* = 2.2, 0.6 Hz, 1H), 7.18 (ddd, *J* = 8.4, 2.1, 0.7 Hz, 1H), 6.92 (dd, *J* = 8.5, 0.6 Hz, 1H), 6.61 (dd, *J* = 8.8, 0.6 Hz, 1H), 5.85 (N-H, s, 2H), 4.35 – 4.26 (m, 4H). $^{13}$C NMR (126 MHz, $CDCl_3$) δ 196.4, 149.3, 147.1, 143.4, 136.5, 135.8, 132.5, 123.7, 120.2, 119.1, 118.8, 117.1, 106.8, 64.7, 64.3.

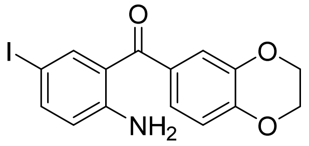

**(2-Amino-5-iodophenyl)(2,3-dihydrobenzo[*b*][1,4]dioxin-6-yl)methanone:** To a 500 mL round bottom flask, *N*-(2-(2,3-dihydrobenzo[*b*][1,4]dioxine-6-carbonyl)-4-iodophenyl)-2,2,2-trifluoroacetamide (5.24 g, 11.0 mmol), sodium hydroxide (2.64 g. 65.9 mmol) was dissolved in MeOH/$H_2O$ (100 mL/200 mL). The flask was capped with water condenser and heated at 60 °C overnight. After the reaction was completed, the solution was condensed *in vacuo* to a slurry solution. The oil solution was added water and extracted with methylene chloride (100 mL x 3). The combined

organic layers were dried with anhydrous sodium sulfate and condensed *in vacuo* to yield an orange solid (4.08 g, 97%). $^1$H NMR (500 MHz, $CDCl_3$) δ 7.72 (d, *J* = 2.1 Hz, 1H), 7.48 (dd, *J* = 8.7, 2.1 Hz, 1H), 7.23 (d, *J* = 2.1 Hz, 1H), 7.17 (dd, *J* = 8.4, 2.1 Hz, 1H), 6.92 (d, *J* = 8.4 Hz, 1H), 6.52 (d, *J* = 8.7 Hz, 1H), 5.86 (N-H, s, 2H), 4.34 – 4.27 (m, 4H). $^{13}$C NMR (126 MHz, $CDCl_3$) δ 196.2, 149.8, 147.1, 143.4, 142.0, 141.8, 132.6, 123.7, 121.0, 119.3, 119.1, 117.1, 64.8, 64.3.

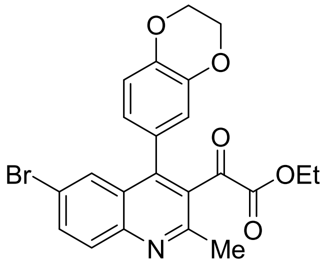


**Ethyl 2-(6-bromo-4-(2,3-dihydrobenzo[*b*][1,4]dioxin-6-yl)-2-methylquinolin-3-yl)-2-oxoacetate:** To 200 mL oven-dried heavy wall pressure vessel round bottom flask, (2-amino-5-bromophenyl)(2,3-dihydrobenzo[*b*][1,4]dioxin-6-yl)methanone (2.51 g, 7.5 mmol), *p*-toluenesulfonic acid monohydrate (285 mg, 1.50 mmol), ethyl-2,4-dioxovalerate (3.69 mL, 26.2 mmol) were dissolved in absolute ethanol (45 mL) and purged with argon gas. The flask was sealed and heated at 80 °C overnight. Upon completion, the product was condensed *in vacuo* to orange oil. The crude product was purified by flash chromatography using ethyl acetate (0-30%) in petroleum ether to give a yellow solid (2.31 g, 68%). $^1$H NMR (500 MHz, $CDCl_3$) δ 7.92 (d, *J* = 8.9 Hz, 1H), 7.84 (d, *J* = 2.2 Hz, 1H), 7.79 (dd, *J* = 8.9, 2.2 Hz, 1H), 6.96 (d, *J* = 8.2 Hz, 1H), 6.79 (d, *J* = 2.1 Hz, 1H), 6.72 (dd, *J* = 8.2, 2.1 Hz, 1H), 4.35 – 4.25 (m, 4H), 3.96 (qd, *J* = 7.2, 1.4 Hz, 2H), 2.69 (s, 3H), 1.11 (t, *J* = 7.1 Hz, 3H). $^{13}$C NMR (126 MHz, $CDCl_3$) δ 189.6, 161.6, 156.1, 146.8, 146.6, 145.0, 143.9, 134.5, 130.8, 130.1, 128.3, 126.4, 126.3, 124.4, 121.0, 120.0, 118.0, 64.6, 64.4, 62.7, 24.1, 14.0.

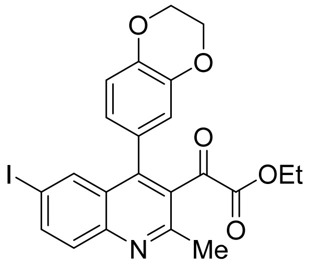


**Ethyl 2-(4-(2,3-dihydrobenzo[*b*][1,4]dioxin-6-yl)-6-iodo-2-methylquinolin-3-yl)-2-oxoacetate:** To 200 mL oven-dried heavy wall pressure vessel round bottom flask, (2-amino-5-iodophenyl)(2,3-dihydrobenzo[b][1,4]dioxin-6-yl)methanone (4.08 g, 10.7 mmol), *p*-toluenesulfonic acid monohydrate (407 mg, 2.14 mmol), ethyl-2,4-dioxovalerate (6.0 mL, 40.7 mmol) were dissolved in absolute ethanol (50 mL) and purged with argon gas. The flask was capped and heated at 80 °C overnight. Upon completion, the product was condensed *in vacuo* to orange oil. The crude product was purified by flash chromatography using ethyl acetate (0-30%) in petroleum ether to give a yellow solid (1.936 g, 36%). $^1$H NMR (500 MHz, $CDCl_3$) δ 8.08 (d, *J* = 1.9 Hz, 1H), 7.99 (dd, *J* = 8.8, 1.9 Hz, 1H), 7.80 (d, *J* = 8.9 Hz, 1H), 6.99 (d, *J* = 8.2 Hz, 1H), 6.82 (d, *J* = 2.1 Hz, 1H), 6.76 (dd, *J* = 8.2, 2.1 Hz, 1H), 4.43 – 4.29 (m, 4H), 3.99 (qd, *J* = 7.2, 1.2 Hz, 2H), 2.72 (s, 3H), 1.15 (t, *J* = 7.2 Hz, 3H). $^{13}$C NMR (126 MHz, $CDCl_3$) δ 189.6, 161.6, 156.2, 147.2, 146.3, 145.0, 143.9, 139.8, 134.9, 130.8, 130.0, 126.8, 126.3, 124.4, 120.0, 118.0, 92.6, 64.6, 64.4, 62.7, 24.2, 14.0.

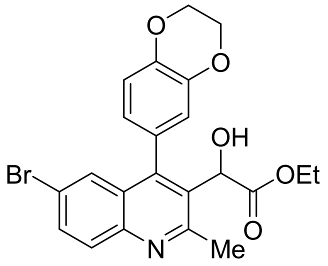


**Ethyl 2-(6-bromo-4-(2,3-dihydrobenzo[*b*][1,4]dioxin-6-yl)-2-methylquinolin-3-yl)-2-hydroxyacetate:** To a 200 mL Schlenk tube, ethyl 2-(6-bromo-4-(2,3-dihydrobenzo[*b*][1,4]dioxin-6-yl)-2-methylquinolin-3-yl)-2-oxoacetate (2.31 g, 5.06 mmol), was dissolved in anhydrous THF (50 mL) and absolute ethanol (10 mL). The flask was placed on an ice bath and purged with argon for 5 min. Sodium borohydride (192 mg, 5.06 mmol) was added portion wise. The flask was sealed and allowed to stir for 3 h. The reaction was quenched with 1M HCl (100 mL) and extracted with ethyl acetate (3x50 mL). The organic layer was dried with anhydrous sodium sulfate and condensed *in vacuo*. The crude product was triturated with petroleum ether to yield an orange powder (2.08 g, 90%). $^1$H NMR (500 MHz, $CDCl_3$) δ 8.13 (d, *J* = 7.1 Hz, 1H), 7.77 (dd, *J* = 9.0, 2.2 Hz, 1H), 7.62 (d, *J* = 2.2 Hz, 1H), 6.99 (dd, *J* = 10.8, 8.1 Hz, 1H), 6.85 (dd, *J* = 15.0, 2.1 Hz, 1H), 6.79 (dd, *J* = 8.2, 2.0 Hz, 1H), 5.42 (s, 1H), 4.42 – 4.29 (m, 3H), 4.27 – 4.07 (m, 2H), 2.83 (s, 3H), 1.27 – 1.19 (m, 3H). $^{13}$C NMR (126 MHz, $CDCl_3$) δ (isomer) 172.9 (172.9), 158.6 (158.6), 144.4 (144.4), 143.7 (143.7), 134.4, 130.2 (130.1), 129.3 (129.3), 128.2, 127.6 127.2 (127.2), 123.0, 122.2, 121.3, 119.0, 118.1, 117.8 (117.7), 69.4 (69.4), 64.5 (64.4), 62.7 (62.6), 60.5, 21.1, 14.2 (14.1).

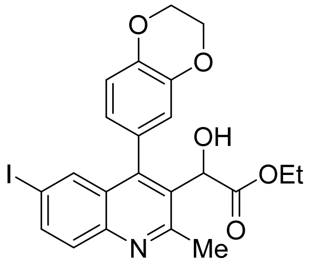

**Ethyl 2-(4-(2,3-dihydrobenzo[*b*][1,4]dioxin-6-yl)-6-iodo-2-methylquinolin-3-yl)-2-hydroxyacetate:** To a 200 mL Schlenk tube, ethyl 2-(4-(2,3-dihydrobenzo[*b*][1,4]dioxin-6-yl)-6-iodo-2-methylquinolin-3-yl)-2-oxoacetate (1.79 g, 3.55 mmol), was dissolved anhydrous THF (40 mL) and 200 proofs ethanol (10 mL). The flask was placed on an ice bath and purged with argon for 5 min. Sodium borohydride (134 mg, 3.55 mmol) was added portion wise. The flask was sealed and allowed to stir for 3h. The reaction was quenched with 1M HCl (50 mL) and extracted with ethyl acetate (3x50 mL). The organic layer was dried with anhydrous sodium sulfate and condensed *in vacuo*. The crude product was triturated with petroleum ether to yield orange powder (537 mg, 30%). $^{1}$H NMR (500 MHz, $CDCl_3$) δ 7.88 (dd, *J* = 8.8, 2.0 Hz, 1H), 7.76 – 7.71 (m, 2H), 6.99 – 6.94 (m, 1H), 6.81 (dd, *J* = 21.5, 2.0 Hz, 1H), 6.74 (ddd, *J* = 13.9, 8.2, 2.1 Hz, 1H), 5.30 (d, *J* = 2.9 Hz, 1H), 4.39 – 4.27 (m, 4H), 4.25 – 4.12 (m, 2H), 2.67 (s, 3H), 1.22 – 1.14 (m, 3H). $^{13}$C NMR (126 MHz, $CDCl_3$) δ (isomer) 173.9 (173.8), 159.0 (159.0), 147.83, 145.85, 144.1 (144.1), 143.7 (143.6), 138.5 (138.5), 135.7 (135.7), 130.2, 128.6 (128.6), 128.2 (128.2), 123.4 (123.3), 122.6 (122.6), 119.3 (119.2), 117.7 (117.5), 91.8, 69.7, 64.5, 62.8, 29.3, 23.9, 14.2.

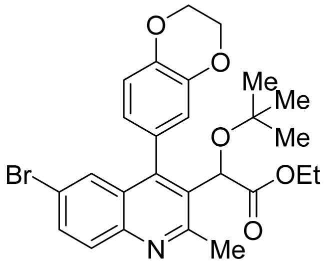

**Ethyl 2-(6-bromo-4-(2,3-dihydrobenzo[b][1,4]dioxin-6-yl)-2-methylquinolin-3-yl)-2-(*tert*-butoxy)acetate:** To a tall glass vial, ethyl 2-(6-bromo-4-(2,3-dihydrobenzo[*b*][1,4]dioxin -6-yl)-2-methylquinolin -3-yl)-2-hydroxyacetate (500 mg, 1.09 mmol) was dissolved in *tert*-butyl acetate (10 mL). The vial was placed on ice. Perchloric acid (312 μL, 2.18 mmol) was added to the solution. The reaction vial was allowed to stir for 3 h. The reaction was neutralized with saturated sodium bicarbonate and extracted with ethyl acetate (3x30 mL). The combined organic layers were dried with anhydrous sodium sulfate and condensed *in vacuo.* Flash column chromatography was performed using hexane: ethyl acetate (4:1) to give a yellow oil (233 mg, 41%). $^{1}$H NMR (500 MHz, $CDCl_3$) δ 7.86 – 7.81 (m, 1H), 7.65 (dq, *J* = 9.1, 2.1 Hz, 1H), 7.52 (dd, *J* = 17.4, 2.2 Hz, 1H), 6.99 – 6.86 (m, 2H), 6.78 – 6.68 (m, 1H), 5.17 (dd, *J* = 5.5, 1.0 Hz, 1H), 4.37 – 4.24 (m, 4H), 4.22 – 4.05 (m, 2H), 2.76 (d, *J* = 1.9 Hz, 3H), 1.20 – 1.16 (m, 3H), 0.95 (dd, *J* = 5.5, 1.4 Hz, 9H). $^{13}$C NMR (126 MHz, $CDCl_3$) δ (isomers) 172.5 (172.4), 160.0, 145.4 (145.3), 144.0, 143.7 (143.3), 132.7 (132.6), 130.8, 130.3 (130.3), 129.0 (128.9), 128.3, 128.0, 123.9, 122.9, 119.9 (119.8), 118.8 (117.6), 117.2, 76.2 (76.2), 70.8 (70.7), 64.5 (64.4) , 61.6 (61.5), 28.2, 24.9, 14.2 (14.2).

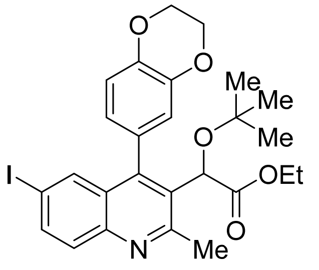

**Ethyl 2-(*tert*-butoxy)-2-(4-(2,3-dihydrobenzo[*b*][1,4]dioxin-6-yl)-6-iodo-2-methylquinolin-3-yl)acetate:** To a tall glass vial, ethyl 2-(4-(2,3-dihydrobenzo[b][1,4]dioxin-6-yl)-6-iodo-2-methylquinolin-3-yl)-2-hydroxyacetate (90 mg, 0.178 mmol) was dissolved in *tert*-butyl acetate (4 mL). The vial was placed on ice. Perchloric acid (46 μL, 0.534 mmol) was added to the solution. The reaction vial was allowed to stir for 3 h. The reaction was neutralized with saturated sodium bicarbonate and extracted with ethyl acetate (3x30 mL). The combined organic layers were dried with anhydrous sodium sulfate and condensed *in vacuo*. Flash chromatography was performed using ethyl acetate (0-20%) in hexanes to give a yellow solid (21 mg, 21%).$^{1}$H NMR (500 MHz, $CDCl_3$) δ 7.87 (ddd, *J* = 8.8, 2.8, 2.0 Hz, 1H), 7.79 – 7.71 (m, 2H), 7.05 – 6.86 (m, 2H), 6.80 – 6.71 (m, 1H), 5.18 (d, *J* = 7.8 Hz, 1H), 4.43 – 4.29 (m, 4H), 4.25 – 4.08 (m, 3H), 2.78 (d, *J* = 1.1 Hz, 3H), 1.27 – 1.18 (m, 3H), 0.99 (d, *J* = 6.4 Hz, 9H).$^{13}$C NMR (126 MHz, $CDCl_3$) δ 172.5 (172.4), 160.3, 145.8, 145.1 (145.1), 144.0 (144.0), 143.7 (143.3), 138.1 (138.0), 135.6 (135.5), 130.7 (130.3), 128.5 (128.4), 124.0, 123.0, 120.0, 118.8, 117.6 (117.2), 91.5 (91.5), 76.3 (76.2), 70.8 (70.7) , 64.6 (64.4), 61.6 (61.5), 28.2, 24.9, 14.2.

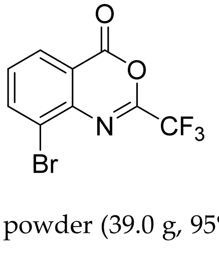

**8-Bromo-2-(trifluoromethyl)-4*H*-benzo[*d*][1,3]oxazin-4-one (10):** To a 200 mL oven-dried heavy wall pressure vessel round bottom flask, 3-bromoanthranilic acid (30.0 g, 138 mmol) was dissolved in trifluoroacetic anhydride (78 mL, 555 mmol). The flask was purged with argon gas and sealed. The reaction was heated up to 130 °C for 24 h. The reaction was quenched with saturated sodium bicarbonate solution to pH 8 and extracted with methylene chloride (3x200 mL). The combined organic layers were dried with anhydrous sodium sulfate and condensed *in vacuo* to yield a brown powder (39.0 g, 95%). $^{1}$H NMR (500 MHz, $CDCl_3$) δ 8.23 (dd, *J* = 7.9, 1.4 Hz, 1H), 8.16 (dd, *J* = 8.0, 1.4 Hz, 1H), 7.55 (t, *J* =

7.9 Hz, 1H). $^{13}$C NMR (126 MHz, $CDCl_3$) δ 156.0, 148.4 – 147.1 (m), 142.4, 141.2, 131.6, 128.6, 123.7, 119.6, 116.1 (q, *J* = 276.3 Hz). $^{19}$F NMR (471 MHz, $CDCl_3$) δ -72.3.

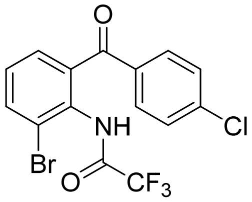

***N*-(2-Bromo-6-(4-chlorobenzoyl)phenyl)-2,2,2-trifluoroacetamide (11a):** To a 200 mL oven-dried Schlenk tube, 8-bromo-2-(trifluoromethyl)-4*H*-benzo[*d*][1,3]oxazin-4-one (13.6 g, 46.2 mmol) was dissolved in anhydrous THF (70 mL). The tube was purged with argon and placed on ice (mixed with table salt) to maintain -10 °C. 4-Chlorophenylmagnesium bromide (70 mL, 70.0 mmol, 1M in THF) was added dropwise. After the addition, the tube was allowed to stir in an ice bath for an additional 3 h. After the reaction was completed, the reaction was quenched with 1M HCl (30 mL), water (100 mL), and extracted with ethyl acetate (3x100 mL). The combined organic layers were dried with anhydrous sodium sulfate and condensed *in vacuo* to yield a yellow solid (8.45 g, 45%). $^{1}$H NMR (500 MHz, $CDCl_3$) δ 8.80 (N-H, s, 1H), 7.81 – 7.75 (m, 3H), 7.47 – 7.44 (m, 2H), 7.38 (dd, *J* = 7.7, 1.4 Hz, 1H), 7.26 (d, *J* = 7.9 Hz, 1H). $^{13}$C NMR (126 MHz, $CDCl_3$) δ 193.4, 155.3 (q, *J* = 38.5 Hz), 140.4, 136.0, 135.8, 134.3, 131.9, 131.2, 129.3, 129.0, 128.3, 121.6, 119.3 – 112.0 (m). $^{19}$F NMR (471 MHz, $CDCl_3$) δ -75.9.

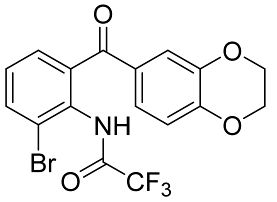

***N*-(2-bromo-6-(2,3-dihydrobenzo[*b*][1,4]dioxine-6-carbonyl)phenyl)-2,2,2-trifluoroacetamide (11b):** To a 200 mL Schlenk tube, 8-bromo-2-(trifluoromethyl)-4*H*-benzo[*d*][1,3]oxazin-4-one (1.53 g, 5.19 mmol) was dissolved in anhydrous THF (15 mL). The tube was purged with argon and placed in ice mixed with table salt to maintain the temperature below -10 °C. To a different tall oven-dried vial, 6-bromobenzodioxane (1.05 mL, 7.78 mmol) was dissolved in THF (15 mL) and purged with argon gas. Turned magnesium (250 mg, 10.4 mmol), and a few crystals of $I_2$ were added. The vial was placed in a warm water bath for 3 min then stirred at room temperature. The reaction was allowed to stir for an additional 10 min after the solution turned colorless. The solution then was loaded onto the syringe and added dropwise to the Schlenk tube. The reaction was allowed to stir for an additional 3 h. The reaction was quenched with 1M HCl (20 mL), water (50 mL), and extracted with ethyl acetate (3x50 mL). The combined organic layers were dried with anhydrous sodium sulfate and condensed *in vacuo*. The crude solid was triturated with ice-cooled diethyl ether to yield a white power (1.49 g, 71%). $^{1}$H NMR (500 MHz, $CDCl_3$) δ 8.81 (N-H, s, 1H), 7.76 (dd, *J* = 8.0, 1.4 Hz, 1H), 7.43 (dd, *J* = 7.6, 1.4 Hz, 1H), 7.40 (d, *J* = 2.1 Hz, 1H), 7.36 (dd, *J* = 8.4, 2.1 Hz, 1H), 7.28 – 7.23 (m, 1H), 6.91 (d, *J* = 8.4 Hz, 1H), 4.33 (ddd, *J* = 5.3, 3.1, 1.1 Hz, 2H), 4.29 – 4.26 (m, 2H). $^{13}$C NMR (126 MHz, $CDCl_3$) δ 193.1, 155.1 (d, *J* = 38.4 Hz), 148.9, 143.4, 136.6, 135.7, 131.4, 129.5, 129.4, 128.3, 125.0, 121.9, 120.0, 117.4, 117.0 – 107.3 (m), 64.9, 64.2. $^{19}$F NMR (471 MHz, $CDCl_3$) δ -75.8.

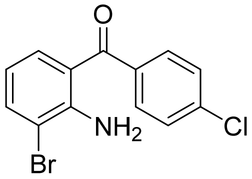

**(2-Amino-3-bromophenyl)(4-chlorophenyl)methanone (12a):** To a 200 mL heavy wall pressure vessel round bottom flask, *N*-(2-bromo-6-(4-chlorobenzoyl)phenyl)-2,2,2-trifluoroacetamide (8.41 g, 20.7 mmol), sodium hydroxide (6.53 mL, 124 mmol, 19M) was dissolved in MeOH/$H_2O$ (100 mL/50 mL). The flask was capped and heated at 60 °C overnight. After the reaction was completed, the solution was evaporated to a slushy solution *in vacuo*. To the oily solution was added water and extracted with methylene chloride (3x100 mL). The combined organic layers were dried with anhydrous sodium sulfate and condensed *in vacuo* to yield a yellow solid (6.31 g, 98%). $^{1}$H NMR (500 MHz, $CDCl_3$) δ 7.59 (ddd, *J* = 7.8, 1.5, 0.7 Hz, 1H), 7.58 – 7.55 (m, 2H), 7.44 – 7.40 (m, 2H), 7.37 (ddd, *J* = 8.1, 1.5, 0.7 Hz, 1H), 6.63 (N-H, s, 2H), 6.49 (td, *J* = 7.9, 0.7 Hz, 1H). $^{13}$C NMR (126 MHz, $CDCl_3$) δ 197.1, 147.9, 137.9, 137.9, 137.5, 133.7, 130.8, 128.6, 118.8, 115.9, 111.3.

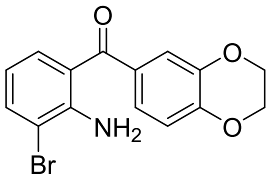

**(2-Amino-3-bromophenyl)(2,3-dihydrobenzo[*b*][1,4]dioxin-6-yl)methanone (12b):** To a 200 mL heavy wall pressure vessel round bottom flask, *N*-(2-bromo-6-(2,3-dihydrobenzo[*b*][1,4]dioxine-6-carbonyl)phenyl)-2,2,2-trifluoroacetamide (1.45 g, 3.57 mmol), sodium hydroxide (856 mg, 21.6 mmol) was dissolved in MeOH/$H_2O$ (60 mL/30 mL). The flask was capped and heated at 60 °C overnight. After the reaction was completed, the solution was evaporated to a slushy solution *in vacuo*. To the oily solution was added water and extracted with methylene chloride (3x100 mL). The combined organic layers were dried with anhydrous sodium sulfate and condensed *in vacuo* to yield a yellow solid (1.32 g, 99%). $^{1}$H NMR (500 MHz, $CDCl_3$) δ 7.57 (dd, *J* = 7.4, 1.0 Hz, 1H), 7.45 (d,

*J* = 7.8 Hz, 1H), 7.26 – 7.23 (m, 1H), 7.19 (ddd, *J* = 8.3, 2.1, 0.9 Hz, 1H), 6.91 (dd, *J* = 8.4, 0.8 Hz, 1H), 6.54 – 6.49 (m, 1H), 6.39 (s, 2H), 4.37 – 4.24 (m, 4H). $^{13}$C NMR (126 MHz, $CDCl_3$) δ 196.9, 147.4, 147.1, 143.2, 136.9, 133.5, 132.8, 123.8, 119.8, 119.3, 117.0, 115.9, 111.1, 64.7, 64.3.

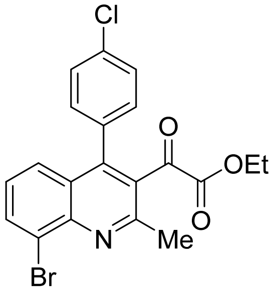


**Ethyl 2-(8-bromo-4-(4-chlorophenyl)-2-methylquinolin-3-yl)-2-oxoacetate (13a):** To 200 mL oven-dried heavy wall pressure vessel round bottom flask, (2-amino-3-bromophenyl)(4-chlorophenyl)methanone (6.31 g, 20.3 mmol), *p*-toluenesulfonic acid monohydrate (773 mg, 4.07 mmol), ethyl-2,4-dioxovalerate (8.6 mL, 61.0 mmol) were dissolved in EtOH (100 mL) and purged with argon gas. The reaction was allowed to stir and heat at 80 °C overnight. After the reaction was completed, the product was condensed *in vacuo* to an orange oil. The crude product was purified by flash chromatography using ethyl acetate (0-15%) in petroleum ether. The product was collected with a small amount of impurity to give a yellow slushy solid. The crude product was triturated with petroleum ether to give a faint yellow solid (3.68 g, 42%). $^{1}$H NMR (500 MHz, $CDCl_3$) δ 8.09 (dd, *J* = 7.5, 1.3 Hz, 1H), 7.55 (dd, *J* = 8.3, 1.3 Hz, 1H), 7.49 – 7.45 (m, 2H), 7.31 (dd, *J* = 8.4, 7.5 Hz, 1H), 7.25 – 7.22 (m, 2H), 3.96 (q, *J* = 7.1 Hz, 2H), 2.80 (s, 3H), 1.12 (t, *J* = 7.1 Hz, 3H). $^{13}$C NMR (126 MHz, $CDCl_3$) δ 189.5, 161.6, 156.6, 146.8, 145.2, 136.0, 134.9, 132.3, 130.3, 129.2, 127.3, 126.1, 125.8, 125.1, 63.0, 24.4, 13.8.

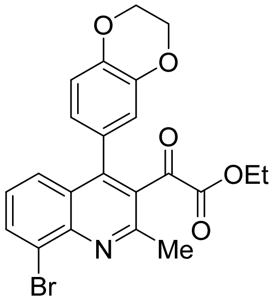


**Ethyl 2-(8-bromo-4-(2,3-dihydrobenzo[*b*][1,4]dioxin-6-yl)-2-methylquinolin-3-yl)-2-oxoacetate (13b):** To 100 mL oven-dried heavy wall pressure vessel round bottom flask, (2-amino-3-bromophenyl)(2,3-dihydrobenzo[*b*][1,4]dioxin-6-yl)methanone (2.57 g, 7.69 mmol), *p*-toluenesulfonic acid monohydrate (203 mg, 1.54 mmol), ethyl-2,4-dioxovalerate (3.78 mL, 26.9 mmol) were dissolved in EtOH (40 mL) and purged with argon gas. The reaction was allowed to stir and heat at 80 °C overnight. After the reaction was completed, the product was condensed *in vacuo* to an orange oil. The crude product was purified by flash chromatography using ethyl acetate (0-15%) in petroleum ether. The product was collected with a small amount of impurity to give a yellow slushy solid. The solid was further triturated with MeOH (10 mL) yield a yellow powder (1.21 g, 35%). $^{1}$H NMR (500 MHz, $CDCl_3$) δ 8.06 (dd, *J* = 7.4, 1.3 Hz, 1H), 7.70 (dd, *J* = 8.4, 1.3 Hz, 1H), 7.31 – 7.27 (m, 1H), 6.95 (d, *J* = 8.2 Hz, 1H), 6.80 (d, *J* = 2.1 Hz, 1H), 6.73 (dd, *J* = 8.2, 2.1 Hz, 1H), 4.32 – 4.26 (m, 4H), 3.97 (qd, *J* = 7.1, 1.2 Hz, 2H), 2.78 (s, 3H), 1.12 (t, *J* = 7.1 Hz, 3H). $^{13}$C NMR (126 MHz, $CDCl_3$) δ 189.7, 161.6, 156.7, 147.8, 145.2, 144.9, 143.8, 134.7, 130.2, 127.0, 126.7, 126.5, 126.3, 124.8, 124.5, 120.1, 117.9, 64.6, 64.4, 62.7, 24.4, 14.0.

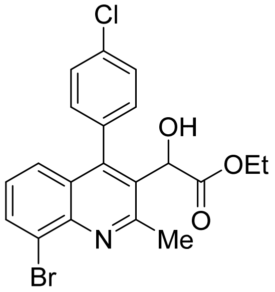


**Ethyl 2-(8-bromo-4-(4-chlorophenyl)-2-methylquinolin-3-yl)-2-hydroxyacetate (14a):** To a 200 mL Schlenk tube, ethyl 2-(8-bromo-4-(4-chlorophenyl)-2-methylquinolin-3-yl)-2-oxoacetate (3.81 g, 8.82 mmol), was dissolved in mixture of anhydrous THF and absolute ethanol (60 mL: 15 mL). The flask was placed on ice and purged with argon for 5 min. Sodium borohydride (334 mg, 8.82 mmol) was added portion wise. The flask was sealed and allowed to stir for 3 h. The reaction was quenched with 1M HCl (50 mL) and extracted with ethyl acetate (3x100 mL). The organic layer was dried with anhydrous sodium sulfate and condensed *in vacuo* to give an orange solid (3.79 g, 99%). $^{1}$H NMR (500 MHz, $CDCl_3$) δ 7.99 (dd, *J* = 7.1, 1.6 Hz, 1H), 7.52 – 7.47 (m, 2H), 7.29 – 7.25 (m, 2H), 7.24 – 7.20 (m, 2H), 5.19 (s, 1H), 4.26 – 4.12 (m, 2H), 2.80 (s, 3H), 1.19 (t, *J* = 7.1 Hz, 3H). $^{13}$C NMR (126 MHz, $CDCl_3$) δ 173.6, 159.5, 144.0, 134.9, 134.3, 134.0, 133.5, 131.6, 130.8, 129.1, 128.8, 127.7, 126.6, 126.5, 69.8, 62.9, 24.4, 14.2.

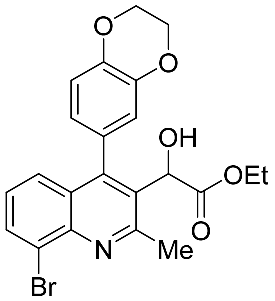

**Ethyl 2-(8-bromo-4-(2,3-dihydrobenzo[*b*][1,4]dioxin-6-yl)-2-methylquinolin-3-yl)-2-hydroxyacetate (14b):** To a 200 mL Schlenk tube, ethyl 2-(8-bromo-4-(2,3-dihydrobenzo[*b*][1,4]dioxin-6-yl)-2-methylquinolin-3-yl)-2-oxoacetate (2.67 g, 5.86 mmol), was dissolved in mixture of anhydrous THF and absolute ethanol (50 mL: 10 mL). The flask was placed on ice and purged with argon for 5 min. Sodium borohydride (222 mg, 5.86 mmol) was added portion wise. The flask was sealed and allowed to stir for 3h. The reaction was quenched with 1M HCl (30 mL) and extracted with ethyl acetate (3x100 mL). The organic layer was dried with anhydrous sodium sulfate and condensed *in vacuo* to give a yellow solid (1.98 g, 74%). $^{1}$H NMR (500 MHz, $CDCl_3$) δ 7.91 (dd, *J* = 7.4, 1.3 Hz, 1H), 7.36 (ddd, *J* = 8.4, 2.1, 1.3 Hz, 1H), 7.14 (dd, *J* = 8.4, 7.4 Hz, 1H), 6.92 (dd, *J* = 8.2, 6.2 Hz, 1H), 6.81 (dd, *J* = 9.7, 2.1 Hz, 1H), 6.76 – 6.68 (m, 1H), 5.32 (s, 1H), 4.29 – 4.20 (m, 4H), 4.18 – 4.07 (m, 2H), 2.75 (s, 3H), 1.15 – 1.10 (m, 3H). $^{13}$C NMR (126 MHz, $CDCl_3$) (isomer) δ 173.9 (173.8), 159.5, 149.1, 143.9 (143.9), 143.6 (143.5), 133.2, 128.8 (128.7), 128.2 (128.1), 127.1, 126.2, 124.3, 123.3, 122.5, 119.3, 118.4, 117.5 (117.4), 69.8, 64.5 (64.5), 62.7 (62.6), 60.5, 24.3, 14.3 (14.1).

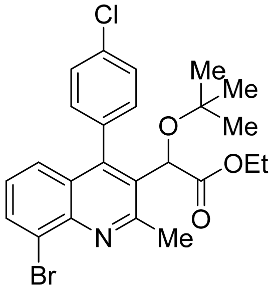

**Ethyl 2-(8-bromo-4-(4-chlorophenyl)-2-methylquinolin-3-yl)-2-(*tert*-butoxy)acetate (15a):** To a 500 mL tri-neck round bottomed flask, ethyl 2-(8-bromo-4-(4-chlorophenyl)-2-methylquinolin-3-yl)-2-hydroxyacetate (3.78 g, 8.71 mmol) was dissolved in *tert*-butyl acetate (60 mL). The vial was placed on ice and purged with argon. Perchloric acid (2.5 mL, 17.4 mmol) was added dropwise to the solution. The reaction vial was allowed to stir for 3 h. The reaction was neutralized with saturated sodium bicarbonate and extracted with ethyl acetate (3x150 mL). The combined organic layers were dried with anhydrous sodium sulfate and condensed *in vacuo.* Flash column chromatography was performed using (0 – 5%) ethyl acetate in hexane to give a faint yellow solid (1.45 g, 34%). $^{1}$H NMR (500 MHz, $CDCl_3$) δ 7.96 (dd, *J* = 7.4, 1.4 Hz, 1H), 7.55 – 7.46 (m, 2H), 7.46 – 7.40 (m, 1H), 7.27 – 7.20 (m, 2H), 7.17 (dd, *J* = 8.4, 7.3 Hz, 1H), 5.06 (s, 1H), 4.24 – 4.08 (m, 2H), 2.90 (s, 3H), 1.21 (t, *J* = 7.1 Hz, 3H), 0.97 (s, 9H). $^{13}$C NMR (126 MHz, $CDCl_3$) (isomer) δ 172.2, 160.8, 145.6, 143.8, 134.9, 134.5, 133.0, 132.3, 131.2, 130.7, 129.0, 128.5, 127.6, 126.4, 126.3, 124.6, 76.3, 70.8, 61.6, 28.2, 25.4, 14.2.

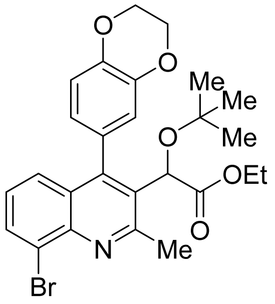

**Ethyl 2-(8-bromo-4-(2,3-dihydrobenzo[*b*][1,4]dioxin-6-yl)-2-methylquinolin-3-yl)-2-(*tert*-butoxy)acetate (15b):** To a 200 mL slenck tube, ethyl 2-(8-bromo-4-(2,3-dihydrobenzo[*b*][1,4]dioxin-6-yl)-2-methylquinolin-3-yl)-2-hydroxyacetate (1.98 g, 4.32 mmol) was dissolved in *tert*-butyl acetate (30 mL). The vial was placed on ice and purged with argon. perchloric acid (1.17 mL, 13.0 mmol) was added dropwise to the solution. The reaction vial was allowed to stir for 3 h. The reaction was neutralized with saturated sodium bicarbonate and extracted with ethyl acetate (3x100 mL). The combined organic layers were dried with anhydrous sodium sulfate and condensed *in vacuo.* Flash column chromatography was performed using (0 – 20%) ethyl acetate in hexane to give a faint yellow solid (1.20 g, 54%). $^{1}$H NMR (500 MHz, $CDCl_3$) δ 7.95 (dt, *J* = 7.4, 1.4 Hz, 1H), 7.43 – 7.36 (m, 1H), 7.18 (ddd, *J* = 8.4, 7.5, 6.0 Hz, 1H), 7.01 – 6.90 (m, 2H), 6.80 – 6.72 (m, 1H), 5.20 (d, *J* = 1.1 Hz, 1H), 4.39 – 4.27 (m, 4H), 4.21 – 4.09 (m, 2H), 2.88 (d, *J* = 1.9 Hz, 3H), 1.23 – 1.19 (m, 3H), 0.99 (d, *J* = 3.7 Hz, 9H). $^{13}$C NMR (126 MHz, $CDCl_3$) (isomer) δ 172.5 (172.4), 160.9 (160.9), 146.4 (146.4), 143.9 (143.9), 143.8 (143.6), 143.2, 132.8 (132.8), 130.9 (130.9), 128.9 (128.9), 128.1 (128.0), 127.0 (126.9), 126.0 (126.0), 124.4 (124.3), 124.0 (123.1), 120.0 (119.0), 117.5 (117.0), 76.2, 70.8 (70.8), 64.6 (64.6), 64.5 (64.5), 61.6 (61.5), 28.2, 25.4, 14.2 (14.2).

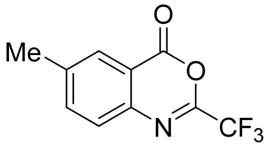

**6-Methyl-2-(trifluoromethyl)-4*H*-benzo[*d*][1,3]oxazin-4-one:** To a 200 mL oven-dried heavy wall pressure vessel round bottom flask, 5-methylanthranilic acid (4.0 g, 26.5 mmol) was dissolved in trifluoroacetic anhydride (15 mL, 105.9 mmol). The flask was purged with argon gas and sealed. The reaction was heated up to 130 °C for 24 h. The reaction was quenched with saturated sodium bicarbonate solution to pH 8 and extracted with ethyl acetate (3x50 mL). The combined organic layers were dried with Anhydrous sodium sulfate and condensed *in vacuo* to yield a brown powder (4.69 g, 78%). $^{1}$H NMR (500 MHz, $CDCl_3$) δ 8.13 – 7.97 (m, 1H), 7.74 – 7.71 (m, 1H), 7.66 (d, *J* = 8.2 Hz, 1H), 2.53 (s, 3H). $^{13}$C NMR (126 MHz, $CDCl_3$) δ 156.8, 146.7 (q, *J* = 42.0 Hz), 142.0, 141.9, 138.6, 129.0, 128.1, 117.6, 120.1 – 112.8 (m), 21.6. $^{19}$F NMR (471 MHz, $CDCl_3$) δ -72.3.

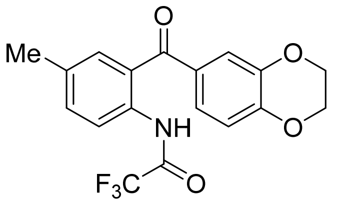


***N*-(2-(2,3-Dihydrobenzo[*b*][1,4]dioxine-6-carbonyl)-4-methylphenyl)-2,2,2-trifluoroacetamide:** To a 250 mL tri-neck round bottom flask, 6-methyl-2-(trifluoromethyl)-4*H*-benzo[*d*][1,3]oxazin-4-one (2.50 g, 10.9 mmol) was dissolved in anhydrous THF (30 mL). The tube was purged with argon and placed in ice mixed with table salt to maintain the temperature below -10 °C. To a different tall oven-dried vial, 6-bromobenzodioxane (2.2 mL, 16.4 mmol) was dissolved in THF (15 mL) and purged with argon gas. Turned magnesium (419 mg, 17.5 mmol), and a few crystals of $I_2$ were added. The vial was placed on a warm water bath and allowed to stir for 5 min. The reaction was allowed to stir for an additional 10 min after the solution turned colorless. The solution then was loaded onto the syringe and added dropwise to the Schlenk tube. The reaction was allowed to stir for an additional 3 h. The reaction was quenched with 1M HCl (20 mL), water (50 mL), and extracted with ethyl acetate (3x50 mL). The combined organic layers were dried with anhydrous sodium sulfate and condensed *in vacuo*. The crude solid was triturated with ice-cooled diethyl ether to yield a white power (1.87 g, 47%). $^1$H NMR (500 MHz, $CDCl_3$) δ 11.66 (s, 1H), 8.43 (d, *J* = 8.4 Hz, 1H), 7.48 – 7.45 (m, 1H), 7.42 (dd, *J* = 8.6, 2.1 Hz, 1H), 7.30 (d, *J* = 2.1 Hz, 1H), 7.25 (dd, *J* = 8.4, 2.1 Hz, 1H), 6.94 (d, *J* = 8.4 Hz, 1H), 4.36 – 4.25 (m, 4H), 2.35 (s, 3H). $^{13}$C NMR (126 MHz, $CDCl_3$) δ 197.9, 155.3 (q, *J* = 37.5 Hz), 148.3, 143.4, 135.2, 134.7, 134.3, 133.8, 131.2, 124.7, 124.6, 121.8, 119.8, 117.3, 115.9 (q, *J* = 288.5 Hz), 64.8, 64.2, 21.0. $^{19}$F NMR (471 MHz, $CDCl_3$) δ -76.0.

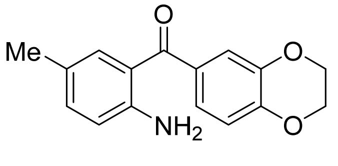


**(2-Amino-5-methylphenyl)(2,3-dihydrobenzo[*b*][1,4]dioxin-6-yl)methanone:** To a 200 mL heavy wall pressure vessel round bottom flask, *N*-(2-(2,3-dihydrobenzo[*b*][1,4] dioxine-6-carbonyl)-4-methylphenyl)-2,2,2-trifluoroacetamide (1.87 g, 5.13 mmol), sodium hydroxide (1.23 g, 30.75 mmol) was dissolved in MeOH/$H_2O$ (40 mL/20 mL). The flask was capped and heated at 60 °C overnight. After the reaction was completed, the solution was evaporated to a slushy solution *in vacuo*. To the oily solution was added water and extracted with methylene chloride (3x50 mL). The combined organic layers were dried with anhydrous sodium sulfate and condensed *in vacuo* to yield a yellow solid (1.24 g, 95%). $^1$H NMR (500 MHz, $CDCl_3$) δ 7.26 – 7.24 (m, 2H), 7.20 (dd, *J* = 8.4, 2.0 Hz, 1H), 7.09 (ddd, *J* = 8.3, 2.1, 0.7 Hz, 1H), 6.91 (d, *J* = 8.4 Hz, 1H), 6.64 (d, *J* = 8.3 Hz, 1H), 4.35 – 4.22 (m, 4H), 2.18 (d, *J* = 0.7 Hz, 3H). $^{13}$C NMR (126 MHz, $CDCl_3$) δ 197.6, 148.3, 146.7, 143.2, 135.0, 133.7, 133.4, 124.8, 123.7, 119.1, 118.9, 117.2, 116.9, 20.5.

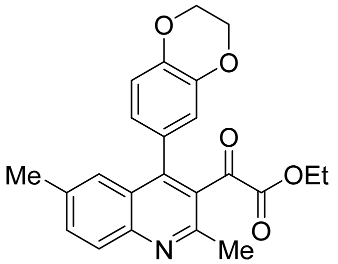


**Ethyl 2-(4-(2,3-dihydrobenzo[*b*][1,4]dioxin-6-yl)-2,6-dimethylquinolin-3-yl)-2-oxoacetate:** To 60 mL oven-dried heavy wall pressure vessel round bottom flask, (2-amino-5-methylphenyl) (2,3-dihydrobenzo[*b*][1,4]dioxin-6-yl)methanone (1.06 g, 3.92 mmol), *p*-toluenesulfonic acid monohydrate (149 mg, 0.784 mmol), ethyl-2,4-dioxovalerate (1.9 mL, 13.7 mmol) were dissolved in EtOH (20 mL) and purged with argon gas. The reaction was allowed to stir and heat at 80 °C overnight. After the reaction was completed, the product was condensed *in vacuo* to orange oil. The crude product was purified by flash chromatography using ethyl acetate (0-20%) in petroleum ether to give a yellow solid (965 mg, 63%). $^1$H NMR (500 MHz, $CDCl_3$) δ 7.98 (d, *J* = 8.5 Hz, 1H), 7.59 (dd, *J* = 8.6, 1.9 Hz, 1H), 7.50 – 7.49 (m, 1H), 6.98 (d, *J* = 8.2 Hz, 1H), 6.85 (d, *J* = 2.1 Hz, 1H), 6.77 (dd, *J* = 8.2, 2.1 Hz, 1H), 4.39 – 4.22 (m, 4H), 3.98 (qd, *J* = 7.2, 1.3 Hz, 2H), 2.74 (s, 3H), 1.15 (t, *J* = 7.1 Hz, 3H). $^{13}$C NMR (126 MHz, $CDCl_3$) δ 190.1, 161.9, 154.5, 146.9, 146.8, 144.7, 143.7, 136.8, 133.4, 129.3, 128.7, 127.3, 125.0, 124.9, 124.5, 120.1, 117.7, 64.5, 64.4, 62.5, 23.9, 21.8, 13.9.

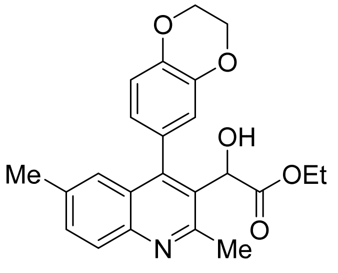


**Ethyl 2-(4-(2,3-dihydrobenzo[*b*][1,4]dioxin-6-yl)-2,6-dimethylquinolin-3-yl)-2-hydroxyacetate:** To a 100 mL tri-neck round bottom flask, ethyl 2-(4-(2,3-dihydrobenzo[*b*][1,4]dioxin-6-yl)-2,6-dimethylquinolin-3-yl)-2-oxoacetate (965 mg, 2.47 mmol), was dissolved in mixture of anhydrous THF and absolute ethanol (20 mL:4 mL). The flask was placed on ice and purged with argon for 5 min. Sodium borohydride (94 mg, 2.47 mmol) was added portionwise. The flask was sealed and allowed to stir for 3 h. The reaction was quenched with 1M HCl (10 mL) and extracted with ethyl acetate (4x20 mL). The organic layer was dried with anhydrous sodium sulfate and condensed *in vacuo*. The product was purified by flash chromatography using 0 - 5% methanol in methylene chloride to give a yellow solid (554

mg, 53%). $^{1}$H NMR (500 MHz, $CDCl_3$) δ 8.06 (dd, *J* = 8.8, 2.6 Hz, 1H), 7.54 – 7.50 (m, 1H), 7.21 – 7.18 (m, 1H), 6.98 (dd, *J* = 11.7, 8.2 Hz, 1H), 6.87 – 6.81 (m, 1H), 6.81 – 6.73 (m, 1H), 5.38 (s, 1H), 4.40 – 4.26 (m, 4H), 4.26 – 4.08 (m, 2H), 2.77 (d, *J* = 1.0 Hz, 3H), 2.41 (d, *J* = 1.0 Hz, 3H), 1.20 (dt, *J* = 8.1, 7.1 Hz, 3H). $^{13}$C NMR (126 MHz, $CDCl_3$) (isomer) δ 173.7 (173.7), 156.9, 144.0 (144.0), 143.6 (143.5), 136.8, 132.8, 128.6 (128.6), 126.8, 126.5, 126.0 (126.0), 123.2, 122.4, 119.1, 118.2, 117.5, 117.4, 69.7 (69.7), 64.5 (64.5), 62.6 (62.6), 22.6, 21.8, 14.1.

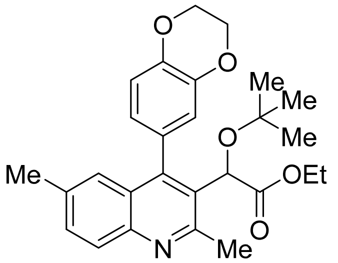

**Ethyl 2-(*tert*-butoxy)-2-(4-(2,3-dihydrobenzo[*b*][1,4]dioxin-6-yl)-2,6-dimethylquinolin-3-yl)acetate:** To a tall vial, ethyl 2-(4-(2,3-dihydrobenzo[*b*][1,4]dioxin-6-yl)-2,6-dimethylquinolin-3-yl)-2-hydroxyacetate (554 mg, 1.41 mmol) was dissolved in *tert*-butyl acetate (12 mL). The vial was placed on ice and purged with argon. Perchloric acid 70% (0.4 mL) was added dropwise to the solution. The reaction vial was allowed to stir for 3 h. The reaction was neutralized with saturated sodium bicarbonate and extracted with ethyl acetate (3x20 mL). The combined organic layers were dried with anhydrous sodium sulfate and condensed *in vacuo.* Flash column chromatography was performed using 20%) ethyl acetate in hexane to give a faint yellow solid (68 mg, 11%). $^{1}$H NMR (500 MHz, $CDCl_3$) δ 7.91 (d, *J* = 8.5 Hz, 1H), 7.46 (dt, *J* = 8.9, 1.8 Hz, 1H), 7.16 (ddt, *J* = 15.6, 1.8, 0.8 Hz, 1H), 7.02 – 6.90 (m, 2H), 6.84 – 6.74 (m, 1H), 5.19 (d, *J* = 5.5 Hz, 1H), 4.41 – 4.30 (m, 4H), 4.25 – 4.09 (m, 2H), 2.79 (d, *J* = 1.7 Hz, 3H), 2.39 (dd, *J* = 5.3, 1.0 Hz, 3H), 1.23 – 1.16 (m, 3H), 0.99 (d, *J* = 4.9 Hz, 9H). $^{13}$C NMR (126 MHz, $CDCl_3$) (isomer) δ 172.8 (172.7), 158.4 (158.4), 145.6 (145.4), 143.7 (143.6), 143.6 (143.1), 135.6 (135.5), 131.5 (131.4), 129.9, 129.4 (129.4), 128.3 (128.3), 126.6 (126.5), 125.7 (125.6), 124.1 (123.2), 120.0, 119.0, 117.4 (116.9), 76.1 (76.0), 70.9 (70.9), 64.6 (64.6), 64.5 (64.4), 61.4 (61.4), 28.2 (28.2), 24.8, 21.9 (21.8), 14.2 (14.2).

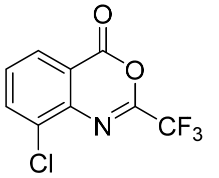

**8-Chloro-2-(trifluoromethyl)-4*H*-benzo[*d*][1,3]oxazin-4-one:** To a 200 mL oven-dried heavy wall pressure vessel round bottom flask, 3-chloroanthranilic acid (5.0 g, 29.1 mmol) was dissolved in trifluoroacetic anhydride (16.4 mL, 116.6 mmol). The flask was purged with argon gas and sealed. The reaction was heated up to 130 °C for 24 h. The reaction was quenched with saturated sodium bicarbonate solution to pH 8 and extracted with ethyl acetate (3x100 mL). The combined organic layers were dried with Anhydrous sodium sulfate, and condensed *in vacuo* to yield brown powder (6.09 g, 84%). $^{1}$H NMR (500 MHz, DMSO-$d_6$) δ 8.14 (d, *J* = 2.2 Hz, 1H), 8.12 (d, *J* = 2.2 Hz, 1H), 7.72 (t, *J* = 7.9 Hz, 1H). $^{13}$C NMR (126 MHz, DMSO-$d_6$) δ 156.8, 147.4 (q, *J* = 41.2 Hz), 141.1, 137.6, 132.0, 131.8, 127.9, 121.4, 119.9 – 113.0 (m). $^{19}$F NMR (471 MHz, DMSO-$d_6$) δ -71.5.

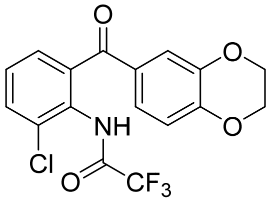

***N*-(2-Chloro-6-(2,3-dihydrobenzo[*b*][1,4]dioxine-6-carbonyl)phenyl)-2,2,2-trifluoroacetamide:** To a 200 mL tri-neck round bottom flask, 8-chloro-2-(trifluoromethyl)-4*H*-benzo[*d*][1,3]oxazin-4-one (2.0 g, 8.0 mmol) was dissolved in anhydrous THF (20 mL). The tube was purged with argon and placed in ice mixed with table salt to maintain the temperature below -10 °C. To a different tall oven-dried vial, 6-bromobenzodioxane (1.6 mL, 12.0 mmol) was dissolved in THF (20 mL) and purged with argon gas. Turned magnesium (385 mg, 16.0 mmol), and a few crystals of $I_2$ were added. The vial was placed on a warm water bath and allowed to stir for 5 min. The reaction was allowed to stir for an additional 10 min after the solution turned colorless. The solution then was loaded onto the syringe and added dropwise to the Schlenk tube. The reaction was allowed to stir for an additional 3 h. The reaction was quenched with 1M HCl (20 mL) and extracted with ethyl acetate (3x100 mL). The combined organic layers were dried with anhydrous sodium sulfate and condensed *in vacuo*. The crude solid was triturated with ice-cooled mixture of diethyl ether: petroleum ether (1:1) to yield brown-red solid (1.88 g, 41%). $^{1}$H NMR (500 MHz, DMSO-$d_6$) δ 11.39 (s, 1H), 7.79 (dd, *J* = 8.0, 1.4 Hz, 1H), 7.51 (t, *J* = 7.9 Hz, 1H), 7.43 (dd, *J* = 7.7, 1.4 Hz, 1H), 7.16 (dd, *J* = 8.5, 2.1 Hz, 1H), 7.10 (d, *J* = 2.1 Hz, 1H), 6.94 (d, *J* = 8.4 Hz, 1H), 4.32 – 4.21 (m, 4H). $^{13}$C NMR (126 MHz, DMSO-$d_6$) δ 192.6, 156.2 – 154.9 (m), 149.0, 143.6, 139.2, 132.8, 132.5, 130.4, 129.9, 128.5, 124.5, 119.1, 122.8 – 115.2 (m), 117.7, 115.0, 65.2, 64.45. $^{19}$F NMR (471 MHz, DMSO-$d_6$) δ -74.3.

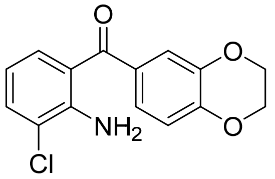

**(2-Amino-3-chlorophenyl)(2,3-dihydrobenzo[*b*][1,4]dioxin-6-yl)methanone:** To a tall vial, *N*-(2-chloro-6-(2,3-dihydrobenzo[*b*][1,4]dioxine-6-carbonyl)phenyl)-2,2,2-trifluoroacetamide (1.87 g, 4.86 mmol), sodium hydroxide (1.17 g, 29.2 mmol) was dissolved in MeOH/$H_2O$ (8 mL/16 mL). The flask was capped and heated at 60 °C for 3 h. After the reaction was completed, the solution was evaporated to a slushy solution *in vacuo*. To the oily solution was added water and extracted with methylene chloride (3x50 mL). The combined organic layers were dried with anhydrous sodium sulfate and condensed *in vacuo* to yield a yellow solid (835 mg, 55%). $^{1}$H NMR (500 MHz, $CDCl_3$) δ 7.43 – 7.36 (m, 2H), 7.23 (d, *J* = 2.1 Hz, 1H), 7.18 (dd, *J* = 8.3, 2.1 Hz, 1H), 6.90 (d, *J* = 8.4 Hz, 1H), 6.55 (t, *J* = 7.9 Hz, 1H), 6.33 (s, 2H), 4.32 – 4.24 (m, 4H). $^{13}$C NMR (126 MHz, $CDCl_3$) δ 196.9, 147.1, 146.5, 143.2, 133.5, 132.8, 132.8, 123.8, 120.5, 119.8, 119.3, 117.0, 115.3, 64.7, 64.3.

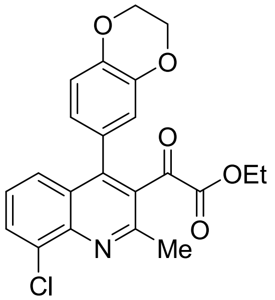

**Ethyl 2-(8-chloro-4-(2,3-dihydrobenzo[*b*][1,4]dioxin-6-yl)-2-methylquinolin-3-yl)-2-oxoacetate:** To tall vial, (2-amino-3-chlorophenyl)(2,3-dihydrobenzo[*b*][1,4]dioxin-6-yl)methanone (835 mg, 2.67 mmol), *p*-toluenesulfonic acid monohydrate (101 mg, 0.533 mmol), ethyl-2,4-dioxovalerate (1.3 mL, 9.33 mmol) were dissolved in EtOH (15 mL) and purged with argon gas. The reaction was allowed to stir and heat at 80 °C overnight. After the reaction was completed, the product was condensed *in vacuo* to orange oil. The crude product was purified by flash chromatography using ethyl acetate (0-20%) in petroleum ether to give a yellow solid (754 mg, 64%). $^{1}$H NMR (500 MHz, $CDCl_3$) δ 7.88 (dt, *J* = 7.4, 1.1 Hz, 1H), 7.68 (dt, *J* = 8.4, 1.1 Hz, 1H), 7.42 – 7.34 (m, 2H), 6.97 (dd, *J* = 8.1, 0.8 Hz, 1H), 6.83 (dd, *J* = 2.0, 0.7 Hz, 1H), 6.76 (ddd, *J* = 8.2, 2.1, 0.8 Hz, 1H), 4.39 – 4.21 (m, 6H), 2.81 (d, *J* = 0.8 Hz, 3H), 1.26 – 1.20 (m, 3H). $^{13}$C NMR (126 MHz, $CDCl_3$) δ 189.7, 161.6, 156.5, 147.8, 144.9, 144.4, 143.8, 133.4, 131.1, 130.2, 126.8, 126.5, 126.4, 125.5, 124.5, 120.1, 117.9, 64.6, 62.6, 58.2, 24.4, 14.0.

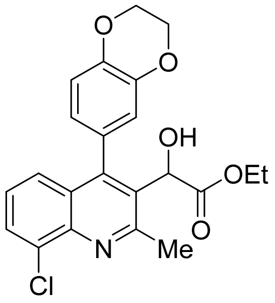

**Ethyl 2-(8-chloro-4-(2,3-dihydrobenzo[*b*][1,4]dioxin-6-yl)-2-methylquinolin-3-yl)-2-hydroxyacetate:** To a 100 mL tri-neck round bottom flask, ethyl 2-(8-chloro-4-(2,3-dihydrobenzo[*b*][1,4]dioxin-6-yl)-2-methylquinolin-3-yl)-2-oxoacetate (700 mg, 1.70 mmol), was dissolved in mixture of anhydrous THF and absolute ethanol (20 mL: 4 mL). The flask was placed on ice and purged with argon for 5 min. Sodium borohydride (65 mg, 1.70 mmol) was added portionwise. The flask was sealed and allowed to stir for 3 h. The reaction was quenched with 1M HCl (10 mL) and extracted with ethyl acetate (4x20 mL). The organic layer was dried with anhydrous sodium sulfate and condensed *in vacuo* to give a yellow solid (603 mg, 86%). $^{1}$H NMR (500 MHz, $CDCl_3$) δ 7.77 (dd, *J* = 7.4, 1.2 Hz, 1H), 7.38 – 7.32 (m, 1H), 7.30 – 7.24 (m, 1H), 6.98 (ddd, *J* = 9.5, 8.2, 1.2 Hz, 1H), 6.87 – 6.81 (m, 1H), 6.79 – 6.75 (m, 1H), 5.33 (d, *J* = 1.1 Hz, 1H), 4.33 (tdd, *J* = 4.9, 3.2, 1.6 Hz, 4H), 4.27 – 4.14 (m, 2H), 2.79 (d, *J* = 1.1 Hz, 3H), 1.19 (qd, *J* = 7.2, 1.2 Hz, 3H). $^{13}$C NMR (126 MHz, $CDCl_3$) (isomer) δ 174.0 (173.9), 159.3, 149.3, 144.0 (144.0), 143.6 (143.5), 143.1, 132.7, 129.8, 128.8, 128.7 (128.6), 128.2, 126.4, 125.8, 123.4 (122.6), 119.3 (118.5), 117.6 (117.4), 69.9 (69.8), 64.5 (64.5), 62.9, 62.8, 24.3 (24.3), 14.1.

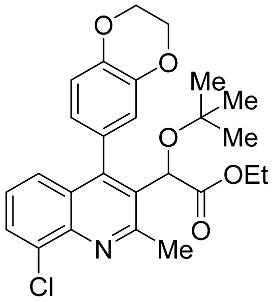

**Ethyl 2-(*tert*-butoxy)-2-(8-chloro-4-(2,3-dihydrobenzo[*b*][1,4]dioxin-6-yl)-2-methylquinolin-3-yl)acetate:** To a tall vial, ethyl 2-(8-chloro-4-(2,3-dihydrobenzo[*b*][1,4]dioxin-6-yl)-2-methylquinolin-3-yl)-2-hydroxyacetate (600 mg, 1.45 mmol) was dissolved in *tert*-butyl acetate (10 mL). The vial was placed on ice and purged with argon. Perchloric acid 70% (373 μL, 4.35 mmol) was added dropwise to the solution. The reaction vial was allowed to stir for 3 h. The reaction was neutralized with saturated sodium bicarbonate and extracted with ethyl acetate (3x20 mL). The combined organic layers were dried with anhydrous sodium sulfate and condensed *in vacuo*. Flash column chromatography was performed using (0 – 20%) ethyl acetate in hexane to give a faint yellow solid (234 mg, 34%). $^{1}$H NMR (500 MHz, $CDCl_3$) δ 7.73 (dt, *J* = 7.4, 1.5 Hz, 1H), 7.35 (ddd, *J* = 20.1, 8.4, 1.4 Hz, 1H), 7.27 – 7.20 (m, 1H), 7.00 – 6.90 (m, 2H), 6.80 – 6.71 (m, 1H), 5.20 (d, *J* = 1.0 Hz, 1H), 4.38 – 4.30 (m, 4H), 4.24 – 4.11 (m, 2H), 2.87 (d, *J* = 1.9 Hz, 3H), 1.27 – 1.21 (m, 3H), 0.98 (d, *J* = 3.7 Hz, 9H). $^{13}$C NMR (126 MHz, $CDCl_3$) (isomer) δ 172.5 (172.4), 160.6 (160.6), 146.4, 144.0 (143.9), 143.6, 143.2 (143.0), 132.8 (132.8), 130.9

(130.9), 129.3 (128.9), 128.1 (128.0), 126.2 (126.1), 125.5 (125.4), 124.0 (123.1), 120.0, 119.0, 117.5 (117.0), 76.2 (76.2), 70.8 (70.8), 64.6 (64.4), 61.6 (61.5), 28.2, 25.4 (25.4), 14.2 (14.2).

**Preparation of 6- and 8-Aryl Quinolines:**

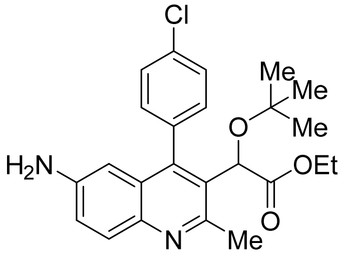


**Ethyl 2-(6-amino-4-(4-chlorophenyl)-2-methylquinolin-3-yl)-2-(tert-butoxy)acetate:** To a tall glass vial, ethyl 2-(6-bromo-4-(4-chlorophenyl)-2-methylquinolin-3-yl)-2-(*tert*-butoxy)acetate (150 mg, 0.308 mmol), L-Proline (46 mg, 0.400 mmol) was dissolved in DMSO (750 μL) and purged with argon gas for 5 min. $NaN_3$ (40 mg, 0.616 mmol) and CuI (59 mg, 0.308 mmol) were added. The solution was allowed to be purged with argon for additional 10 min. The vial was capped and heated at 100 °C for 3 h. After the reaction complete, the solution was quenched with saturated solution of $NH_4Cl$ (5 mL) and water (10 mL). The solution was extracted with ethyl acetate (3x20 mL). The combined organic layers were dried with anhydrous sodium sulfate, and condensed *in vacuo*. Flash chromatography was performed using (30-50%) ethyl acetate in hexane to give a brown solid (75 mg, 57%). $^1$H NMR (500 MHz, $CDCl_3$) δ 7.83 (d, *J* = 8.9 Hz, 1H), 7.52 – 7.46 (m, 2H), 7.43 – 7.37 (m, 1H), 7.25 – 7.22 (m, 1H), 7.08 (dd, *J* = 8.9, 2.6 Hz, 1H), 6.31 (d, *J* = 2.4 Hz, 1H), 5.01 (s, 1H), 4.21 – 4.06 (m, 2H), 3.81 (bs, 2H), 2.75 (s, 3H), 1.20 (t, *J* = 7.1 Hz, 3H), 0.97 (s, 9H). $^{13}$C NMR (126 MHz, $CDCl_3$) δ 172.6, 155.3, 144.4, 143.4, 141.8, 135.3, 134.4, 132.4, 131.3, 129.8, 128.9, 128.4, 127.6, 121.3, 106.8, 76.1, 70.9, 61.4, 28.2, 24.4, 14.2.

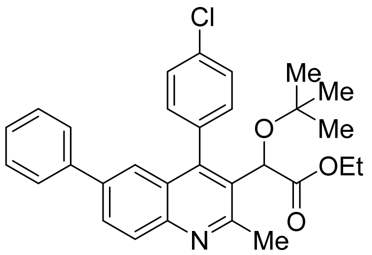


**Ethyl 2-(*tert*-butoxy)-2-(4-(4-chlorophenyl)-2-methyl-6-phenylquinolin-3-yl)acetate:** To a tall glass vial, ethyl 2-(*tert*-butoxy)-2-(4-(4-chlorophenyl)-6-iodo-2-methylquinolin-3-yl)acetate (100 mg, 0.186 mmol), $Na_2CO_3$ (40 mg, 0.372 mmol), tetrakis(triphenylphosphine) palladium (11 mg, 0.009 mmol), phenylboronic acid (34 mg, 0.279 mmol) in the mixture of 1,4-dioxane: $H_2O$ (2 mL:1 mL) was purged with nitrogen for 5 min and was capped. The reaction was stirred overnight at 80 °C. The solids were removed by filtration over Celite and washed with ethyl acetate. The filtrate was condensed *in vacuo*. Flash chromatography was performed using hexane: ethyl acetate (5:1) eluent to give light yellow oil (65 mg, 72%). $^1$H NMR (500 MHz, $CDCl_3$) δ 8.11 (dd, *J* = 8.6, 1.3 Hz, 1H), 7.92 (dt, *J* = 8.8, 1.8 Hz, 1H), 7.55 – 7.47 (m, 5H), 7.45 (dt, *J* = 2.0, 1.0 Hz, 1H), 7.44 – 7.39 (m, 2H), 7.36 – 7.30 (m, 2H), 5.09 (d, *J* = 1.4 Hz, 1H), 4.26 – 4.12 (m, 2H), 2.85 (d, *J* = 1.4 Hz, 3H), 1.24 (td, *J* = 7.1, 1.4 Hz, 3H), 1.00 (d, *J* = 1.3 Hz, 9H). $^{13}$C NMR (126 MHz, $CDCl_3$) δ 172.5, 159.5, 146.3, 145.5, 140.7, 138.9, 134.8, 134.7, 132.4, 131.3, 130.2, 129.2, 129.0, 129.0, 128.5, 127.7, 127.5, 126.3, 124.3, 76.2, 70.9, 61.6, 28.2, 25.0, 14.3.

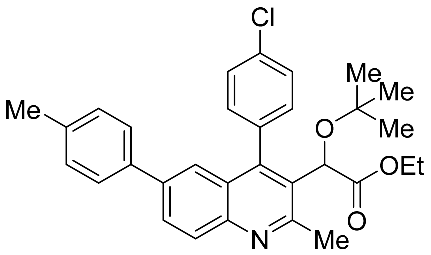


**Ethyl 2-(*tert*-butoxy)-2-(4-(4-chlorophenyl)-2-methyl-6-(*o*-tolyl)quinolin-3-yl)acetate:** To a tall glass vial, ethyl 2-(6-bromo-4-(4-chlorophenyl)-2-methylquinolin-3-yl)-2-(*tert*-butoxy)acetate (192 mg, 0.393 mmol), $Na_2CO_3$ (83 mg, 0.786 mmol), tetrakis(triphenylphosphine)palladium (23 mg, 0.020 mmol), 4-methylphenylboronic acid (80 mg, 0.589 mmol) in the mixture of 1,4-dioxane: $H_2O$ (2 mL:1 mL) was purged with nitrogen for 5 min and was capped. The reaction was stirred overnight at 80 °C. The solids were removed by filtration over Celite and washed with ethyl acetate. The filtrate was condensed *in vacuo*. Flash chromatography was performed using hexane: ethyl acetate (5:1) eluent to give light yellow oil (129 mg, 65%). $^1$H NMR (500 MHz, $CDCl_3$) δ 8.09 (dd, *J* = 8.6, 0.6 Hz, 1H), 7.90 (dd, *J* = 8.7, 2.0 Hz, 1H), 7.55 – 7.50 (m, 2H), 7.50 – 7.46 (m, 1H), 7.42 (dd, *J* = 2.0, 0.6 Hz, 1H), 7.41 – 7.38 (m, 2H), 7.32 – 7.29 (m, 1H), 7.24 – 7.20 (m, 2H), 5.09 (s, 1H), 4.24 – 4.12 (m, 2H), 2.85 (s, 3H), 2.37 (s, 3H), 1.23 (t, *J* = 7.1 Hz, 3H), 0.99 (s, 9H). $^{13}$C NMR (126 MHz, $CDCl_3$) δ 172.5, 159.3, 146.1, 145.5, 138.9, 137.8, 137.6, 134.8, 134.7, 132.4, 131.3, 129.7, 129.2, 129.1, 129.0, 128.5, 127.3, 126.4, 123.9, 76.2, 70.9, 61.6, 28.2, 24.9, 21.2, 14.3.

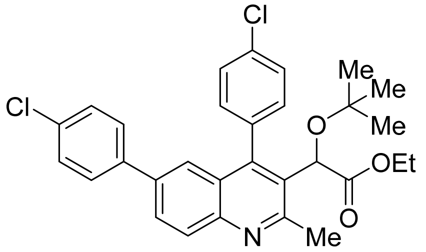


**Ethyl 2-(4,6-bis(4-chlorophenyl)-2-methylquinolin-3-yl)-2-(*tert*-butoxy)acetate:** To a tall glass vial, ethyl 2-(*tert*-butoxy)-2-(4-(4-chlorophenyl)-6-iodo-2-methylquinolin-3-yl)acetate (83 mg, 0.154 mmol), $Na_2CO_3$ (33 mg, 0.308 mmol), tetrakis(triphenylphosphine)palladium (9 mg, 0.008 mmol), 4-chlorophenylboronic acid (36 mg, 0.231 mmol) in the mixture of 1,4-dioxane: $H_2O$ (2 mL:1 mL) was purged with nitrogen for 5 min and was capped. The reaction was stirred overnight at 80 °C. The solids were removed by filtration over Celite and washed with ethyl acetate. The filtrate was condensed *in vacuo*. Flash chromatography was performed using hexane: ethyl acetate (5:1) eluent to give light yellow oil (73 mg, 91%). $^1H$ NMR (500 MHz, $CDCl_3$) δ 8.12 (dd, *J* = 8.7, 0.6 Hz, 1H), 7.86 (dd, *J* = 8.7, 2.1 Hz, 1H), 7.57 – 7.52 (m, 2H), 7.50 – 7.47 (m, 1H), 7.42 – 7.37 (m, 3H), 7.32 – 7.29 (m, 1H), 7.16 – 7.12 (m, 1H), 6.84 – 6.81 (m, 1H), 5.10 (s, 1H), 4.24 – 4.12 (m, 2H), 2.86 (s, 3H), 1.24 (t, *J* = 7.1 Hz, 3H), 1.00 (s, 9H). $^{13}C$ NMR (126 MHz, $CDCl_3$) δ 172.5, 159.8, 155.7, 146.0, 139.0, 137.9, 135.0, 134.4, 133.9, 132.3, 131.2, 130.5, 129.5, 129.2, 129.2, 129.1, 129.0, 128.7, 128.6, 126.4, 124.2, 117.1, 76.4, 70.8, 61.7, 28.2, 24.7, 14.3.

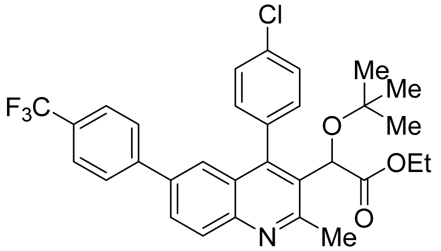


**Ethyl 2-(*tert*-butoxy)-2-(4-(4-chlorophenyl)-2-methyl-6-(4-(trifluoromethyl)phenyl) quinolin-3-yl)acetate:** To a tall glass vial, ethyl 2-(*tert*-butoxy)-2-(4-(4-chlorophenyl)-6-iodo-2-methylquinolin-3-yl)acetate (112 mg, 0.208 mmol), $Na_2CO_3$ (44 mg, 0.416 mmol), tetrakis(triphenylphosphine)palladium (12 mg, 0.010 mmol), 4-trifluoromethylphenylboronic acid (66 mg, 0.312 mmol) in the mixture of 1,4-dioxane: $H_2O$ (2 mL:1 mL) was purged with nitrogen for 5 min and was capped. The reaction was stirred overnight at 80 °C. The solids were removed by filtration over Celite and washed with ethyl acetate. The filtrate was condensed *in vacuo*. Flash chromatography was performed using hexane: ethyl acetate (5:1) eluent to give light yellow oil (108 mg, 94%). $^1H$ NMR (500 MHz, $CDCl_3$) δ 8.14 (dd, *J* = 8.7, 0.5 Hz, 1H), 7.90 (dd, *J* = 8.7, 2.1 Hz, 1H), 7.67 (d, *J* = 8.2 Hz, 2H), 7.61 – 7.58 (m, 2H), 7.57 – 7.52 (m, 2H), 7.51 – 7.45 (m, 2H), 7.33 – 7.29 (m, 1H), 5.10 (s, 1H), 4.25 – 4.14 (m, 2H), 2.86 (s, 3H), 1.24 (t, *J* = 7.1 Hz, 3H), 1.00 (s, 9H). $^{13}C$ NMR (126 MHz, $CDCl_3$) δ 172.4, 160.2, 146.5, 145.6, 144.2, 137.4, 135.0, 134.4, 132.4, 131.2, 130.5, 129.6, 129.1, 128.8, 128.6, 127.8, 130.0 – 123.1 (m), 126.3, 125.93 (q, J = 3.6 Hz), 124.8, 76.3, 70.9, 61.6, 28.2, 25.0, 14.2.

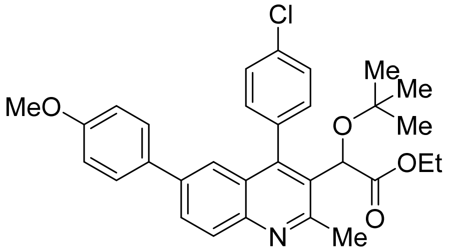


**Ethyl 2-(*tert*-butoxy)-2-(4-(4-chlorophenyl)-6-(4-methoxyphenyl)-2-methylquinolin-3-yl)acetate:** To a tall glass vial, ethyl 2-(*tert*-butoxy)-2-(4-(4-chlorophenyl)-6-iodo-2-methylquinolin-3-yl)acetate (140 mg, 0.260 mmol), $Na_2CO_3$ (55 mg, 0.520 mmol), tetrakis(triphenylphosphine) palladium (15 mg, 0.013 mmol), 4-methoxyphenylboronic acid (59 mg, 0.390 mmol) in the mixture of 1,4-dioxane: $H_2O$ (2 mL:1 mL) was purged with nitrogen for 5 min and was capped. The reaction was stirred overnight at 80 °C. The solids were removed by filtration over Celite and washed with ethyl acetate. The filtrate was condensed *in vacuo*. Flash chromatography was performed using hexane: ethyl acetate (5:1) eluent to give light yellow oil (124 mg, 93%). $^1H$ NMR (500 MHz, $CDCl_3$) δ 8.10 (d, *J* = 8.7 Hz, 1H), 7.89 (dd, *J* = 8.7, 2.1 Hz, 1H), 7.55 – 7.51 (m, 2H), 7.49 – 7.46 (m, 1H), 7.43 (d, *J* = 8.9 Hz, 1H), 7.39 (dd, *J* = 2.0, 0.6 Hz, 1H), 7.33 – 7.29 (m, 1H), 6.95 (d, *J* = 8.8 Hz, 1H), 5.09 (s, 1H), 4.28 – 4.00 (m, 2H), 3.83 (s, 3H), 2.85 (s, 3H), 1.23 (t, *J* = 7.1 Hz, 3H), 0.99 (s, 9H). $^{13}C$ NMR (126 MHz, $CDCl_3$) δ 172.5, 159.5, 159.1, 145.5, 138.6, 134.8, 134.7, 133.1, 132.4, 131.3, 130.1, 129.1, 129.0, 128.5, 128.5, 126.4, 123.4, 114.5, 76.2, 70.9, 61.6, 55.5, 28.2, 24.8, 14.2.

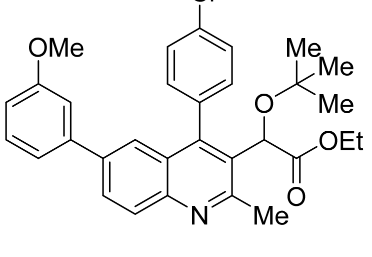


**Ethyl 2-(*tert*-butoxy)-2-(4-(4-chlorophenyl)-6-(3-methoxyphenyl)-2-methylquinolin-3-yl)acetate:** To a tall glass vial, ethyl 2-(6-bromo-4-(4-chlorophenyl)-2-methylquinolin-3-yl)-2-(*tert*-butoxy)acetate (107 mg, 0.219 mmol), $Na_2CO_3$ (46 mg, 0.438 mmol), tetrakis(triphenylphosphine)palladium (13 mg, 0.011 mmol), 3-methoxyphenylboronic acid (50 mg, 0.329 mmol) in the mixture of 1,4-dioxane: $H_2O$ (2 mL:1 mL) was purged with nitrogen for 5 min and was capped. The reaction was stirred overnight at 80 °C. The solids were removed by filtration over Celite and washed with ethyl acetate. The filtrate was condensed *in vacuo*. Flash chromatography was performed using hexane:

ethyl acetate (5:1) eluent to give light yellow oil (101 mg, 89%). $^{1}$H NMR (500 MHz, $CDCl_3$) δ 8.10 (dd, *J* = 8.7, 0.6 Hz, 1H), 7.90 (dd, *J* = 8.7, 2.0 Hz, 1H), 7.56 – 7.50 (m, 2H), 7.50 – 7.45 (m, 1H), 7.44 (dd, *J* = 2.1, 0.6 Hz, 1H), 7.36 – 7.26 (m, 2H), 7.07 (ddd, *J* = 7.7, 1.8, 1.0 Hz, 1H), 7.03 (dd, *J* = 2.6, 1.7 Hz, 1H), 6.88 (ddd, *J* = 8.3, 2.6, 0.9 Hz, 1H), 5.09 (s, 1H), 4.27 – 4.12 (m, 2H), 3.83 (s, 3H), 2.85 (s, 3H), 1.23 (t, *J* = 7.1 Hz, 3H), 0.99 (s, 9H). $^{13}$C NMR (126 MHz, $CDCl_3$) δ 172.5, 160.0, 159.6, 146.3, 145.5, 142.3, 138.8, 134.8, 134.7, 132.4, 131.3, 130.2, 130.0, 129.2, 129.2, 129.0, 128.5, 126.3, 124.3, 120.0, 113.6, 112.7, 76.2, 70.9, 61.6, 55.4, 28.2, 25.0 14.2.

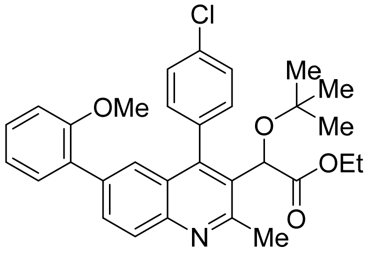


**Ethyl 2-(*tert*-butoxy)-2-(4-(4-chlorophenyl)-6-(2-methoxyphenyl)-2-methylquinolin-3-yl)acetate:** To a tall glass vial, ethyl 2-(6-bromo-4-(4-chlorophenyl)-2-methylquinolin-3-yl)-2-(*tert*-butoxy)acetate (71 mg, 0.145 mmol), $Na_2CO_3$ (31 mg, 0.290 mmol), tetrakis(triphenylphosphine)palladium (8 mg, 0.007 mmol), 2-methoxyphenylboronic acid (33 mg, 0.217 mmol) in the mixture of 1,4-dioxane: $H_2O$ (2 mL:1 mL) was purged with nitrogen for 5 min and was capped. The reaction was stirred overnight at 80 °C. The solids were removed by filtration over Celite and washed with ethyl acetate. The filtrate was condensed *in vacuo*. Flash chromatography was performed using hexane: ethyl acetate (5:1) eluent to give light yellow oil (87 mg, 99%). $^{1}$H NMR (500 MHz, $CDCl_3$) δ 8.05 (dd, *J* = 8.6, 0.6 Hz, 1H), 7.86 (dd, *J* = 8.6, 2.0 Hz, 1H), 7.52 – 7.45 (m, 3H), 7.41 (dd, *J* = 2.0, 0.6 Hz, 1H), 7.34 – 7.29 (m, 2H), 7.23 (dd, *J* = 7.5, 1.8 Hz, 1H), 7.02 – 6.94 (m, 2H), 5.10 (s, 1H), 4.25 – 4.10 (m, 2H), 3.77 (s, 3H), 2.85 (s, 3H), 1.30 – 1.18 (m, 3H), 0.99 (s, 9H). $^{13}$C NMR (126 MHz, $CDCl_3$) δ 172.5, 159.3, 156.6, 146.0, 145.4, 136.4, 134.9, 134.6, 132.4, 131.6, 131.4, 131.0, 130.1, 129.8, 129.1, 128.9, 126.7, 126.0, 121.0, 111.3, 76.2, 70.9, 61.5, 55.5, 28.2, 25.0, 14.3.

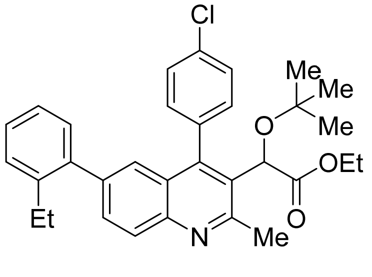


**Ethyl 2-(*tert*-butoxy)-2-(4-(4-chlorophenyl)-6-(2-ethylphenyl)-2-methylquinolin-3-yl)acetate:** To a tall glass vial, ethyl 2-(6-bromo-4-(4-chlorophenyl)-2-methylquinolin-3-yl)-2-(*tert*-butoxy)acetate (183 mg, 0.375 mmol), $Na_2CO_3$ (80 mg, 0.750 mmol), tetrakis(triphenylphosphine)palladium (22 mg, 0.019 mmol), 2-ethylphenylboronic acid (84 mg, 0.562 mmol) in the mixture of 1,4-dioxane: $H_2O$ (2 mL:1 mL) was purged with nitrogen for 5 min and was capped. The reaction was stirred overnight at 80 °C. The solids were removed by filtration over Celite and washed with ethyl acetate. The filtrate was condensed *in vacuo*. Flash chromatography was performed using hexane: ethyl acetate (5:1) eluent to give light yellow oil (168 mg, 87%). $^{1}$H NMR (500 MHz, $CDCl_3$) δ 8.09 (dd, *J* = 8.5, 0.6 Hz, 1H), 7.65 – 7.62 (m, 1H), 7.51 – 7.43 (m, 3H), 7.31 – 7.26 (m, 3H), 7.23 – 7.19 (m, 2H), 7.17 – 7.13 (m, 1H), 5.10 (s, 1H), 4.27 – 4.10 (m, 2H), 2.87 (s, 3H), 2.51 (q, *J* = 7.6 Hz, 2H), 1.27 – 1.24 (m, 3H), 1.04 – 0.98 (m, 12H). $^{13}$C NMR (126 MHz, $CDCl_3$) δ 172.5, 159.5, 145.6 (d, *J* = 36.6 Hz), 141.8, 141.0, 139.7, 134.7 (d, *J* = 10.1 Hz), 132.2, 131.4, 131.1, 130.2, 130.1, 129.0, 128.9, 128.5, 128.3, 127.9, 126.3, 125.9, 125.8, 120.3, 115.2, 76.3, 70.9, 61.6, 28.2, 26.2, 24.9, 15.8, 14.3.

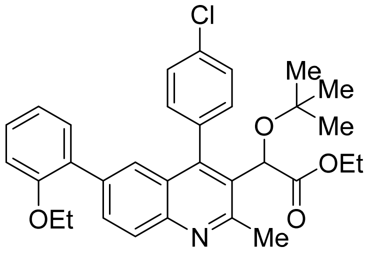


**Ethyl 2-(*tert*-butoxy)-2-(4-(4-chlorophenyl)-6-(2-ethoxyphenyl)-2-methylquinolin-3-yl)acetate:** To a tall glass vial, ethyl 2-(6-bromo-4-(4-chlorophenyl)-2-methylquinolin-3-yl)-2-(*tert*-butoxy)acetate (156 mg, 0.318 mmol), $Na_2CO_3$ (67 mg, 0.636 mmol), tetrakis(triphenylphosphine)palladium (19 mg, 0.016 mmol), 2-ethoxyphenylboronic acid (79 mg, 0.477 mmol) in the mixture of 1,4-dioxane: $H_2O$ (2 mL:1 mL) was purged with nitrogen for 5 min and was capped. The reaction was stirred overnight at 80 °C. The solids were removed by filtration over Celite and washed with ethyl acetate. The filtrate was condensed *in vacuo*. Flash chromatography was performed using hexane: ethyl acetate (4:1) eluent to give light yellow oil (158 mg, 93%). $^{1}$H NMR (500 MHz, $CDCl_3$) δ 8.06 (dd, *J* = 8.8, 0.6 Hz, 1H), 7.91 – 7.86 (m, 1H), 7.52 – 7.45 (m, 4H), 7.33 – 7.25 (m, 3H), 7.02 – 6.97 (m, 1H), 6.95 – 6.92 (m, 1H), 5.08 (s, 1H), 4.23 – 4.16 (m, 2H), 3.98 (qd, *J* = 7.0, 4.9 Hz, 2H), 2.85 (s, 3H), 1.26 – 1.22 (m, 6H), 1.00 (s, 9H).$^{13}$C NMR (126 MHz, $CDCl_3$) δ 172.6, 159.2, 155.9, 145.9, 145.5, 136.5, 134.9, 134.6, 132.4, 131.8, 131.3, 131.1, 130.1, 129.8, 129.0, 128.9, 128.4, 128.0 126.8, 125.9, 120.9, 112.4, 76.2, 70.9, 63.9, 61.5, 28.2, 24.9, 14.8, 14.3.

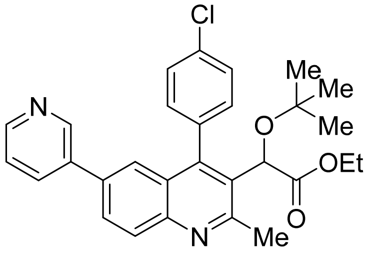

**Ethyl 2-(*tert*-butoxy)-2-(4-(4-chlorophenyl)-2-methyl-6-(pyridin-3-yl)quinolin-3-yl)acetate:** To a tall glass vial, ethyl 2-(*tert*-butoxy)-2-(4-(4-chlorophenyl)-6-iodo-2-methylquinolin-3-yl)acetate (92 mg, 0.171 mmol), $Na_2CO_3$ (36 mg, 0.342 mmol), tetrakis(triphenylphosphine)palladium (10 mg, 0.009 mmol), 3-pyridinylboronic acid (37 mg, 0.285 mmol) in the mixture of 1,4-dioxane: $H_2O$ (2 mL:1 mL) was purged with nitrogen for 5 min and was capped. The reaction was stirred overnight at 80 °C. The solids were removed by filtration over Celite and washed with ethyl acetate. The filtrate was condensed *in vacuo*. Flash chromatography was performed using hexane: ethyl acetate (1:2) eluent to give light yellow oil (70 mg, 88%). $^1$H NMR (500 MHz, $CDCl_3$) δ 8.74 (d, *J* = 2.0 Hz, 1H), 8.55 (dd, *J* = 4.8, 1.6 Hz, 1H), 8.13 (d, *J* = 8.7 Hz, 1H), 7.87 (dd, *J* = 8.7, 1.9 Hz, 1H), 7.82 – 7.72 (m, 1H), 7.55 – 7.50 (m, 2H), 7.50 – 7.42 (m, 2H), 7.37 – 7.27 (m, 2H), 5.08 (s, 1H), 4.24 – 4.13 (m, 2H), 2.85 (s, 3H), 1.22 (td, *J* = 7.1, 0.6 Hz, 3H), 0.98 (s, 9H). $^{13}$C NMR (126 MHz, $CDCl_3$) δ 172.4, 160.2, 148.8, 148.6, 146.5, 145.5, 136.2, 135.5, 135.0, 134.6, 134.4, 132.3, 131.2, 130.5, 129.7, 129.2, 128.6, 128.6, 126.4, 124.6, 123.7, 76.3, 70.8, 61.6, 28.2, 25.0, 14.3.

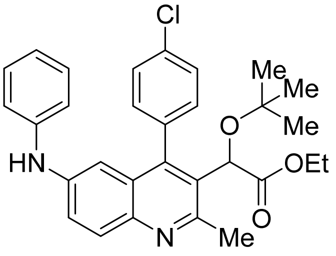

**Ethyl 2-(*tert*-butoxy)-2-(4-(4-chlorophenyl)-2-methyl-6-(phenylamino)quinolin-3-yl)acetate:** To a tall glass vial, ethyl 2-(6-bromo-4-(4-chlorophenyl)-2-methylquinolin-3-yl)-2-(*tert*-butoxy)acetate (176 mg, 0.360 mmol), aniline (40 µL, 0.432 mmol), JohnPhos (5.4 mg, 0.018 mmol), $Pd(OAc)_2$ (2 mg, 0.009 mmol), $Cs_2CO_3$ (235 mg, 0.72 mmol) were dissolved in 1,4 dioxane was purged with nitrogen for 5 min and was capped. The reaction was stirred overnight at 100 °C. The solids were removed by filtration over Celite and washed with ethyl acetate. The filtrate was condensed *in vacuo*. Flash chromatography was performed using hexane: ethyl acetate (6:1) eluent to give red solid (132 mg, 73%). $^1$H NMR (500 MHz, $CDCl_3$) δ 7.92 (d, *J* = 8.9 Hz, 1H), 7.51 – 7.41 (m, 4H), 7.28 – 7.25 (m, 1H), 7.24 – 7.19 (m, 2H), 7.02 – 6.97 (m, 2H), 6.93 (tt, *J* = 7.4, 1.1 Hz, 1H), 6.77 (d, *J* = 2.5 Hz, 1H), 5.95 (N-H, s, 1H), 5.06 (s, 1H), 4.18 (qq, *J* = 10.9, 7.1 Hz, 2H), 2.79 (s, 3H), 1.23 (t, *J* = 7.1 Hz, 3H), 0.99 (s, 9H). $^{13}$C NMR (126 MHz, $CDCl_3$) δ 172.6, 156.5, 143.8, 143.0, 142.5, 140.9, 135.0, 134.6, 132.4, 131.3, 130.0, 130.0, 129.5, 129.0, 128.3, 127.4, 122.6, 121.7, 117.9, 110.4, 76.2, 70.9, 61.5, 28.2, 24.6, 14.3.

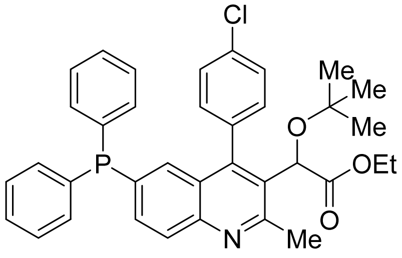

**Ethyl 2-(tert-butoxy)-2-(4-(4-chlorophenyl)-6-(diphenylphosphaneyl)-2-methylquinolin-3-yl)acetate:** To a tall glass vial, ethyl 2-(*tert*-butoxy)-2-(4-(4-chlorophenyl)-6-iodo-2-methylquinolin-3-yl)acetate (125 mg, 0.232 mmol), $Pd(OAc)_2$ (1.4 mg, 0.006 mmol), DiPPF (3 mg, 0.08 mmol), NaO*t*Bu (42 mg, 0.337 mmol) were dissolved in toluene (2 mL). The vial was purged with argon, capped, and allowed to stir at room temperature for 2 h. Diphenylphosphine (59 µL, 0.337 mmol) was added. The vial was then re-purged with argon, capped, and refluxed at 110 °C for 16 h. After the reaction complete, the solids were removed by filtration over Celite and washed with ethyl acetate. The filtrate was condensed *in vacuo*. Flash chromatography was performed using (0 – 20%) ethyl acetate in hexane eluent to give a clear colorless oil (231 mg, 99%). $^1$H NMR (500 MHz, $CDCl_3$) δ 7.98 (ddd, *J* = 8.6, 1.6, 0.6 Hz, 1H), 7.69 – 7.50 (m, 3H), 7.48 – 7.24 (m, 10H), 7.22 – 7.18 (m, 2H), 7.02 – 6.92 (m, 1H), 5.08 (d, *J* = 11.5 Hz, 1H), 4.22 – 4.13 (m, 2H), 2.90 – 2.73 (m, 3H), 1.27 – 1.20 (m, 3H), 0.98 (d, *J* = 12.1 Hz, 9H). $^{13}$C NMR (126 MHz, $CDCl_3$) δ 172.5, 160.3, 146.8, 145.4, 136.6, 136.5, 135.5, 134.5, 134.1, 133.8, 133.7, 132.4, 132.2, 132.1, 132.0 131.3, 131.0, 130.0, 129.5, 129.0, 129.0, 129.0, 128.9, 128.7, 128.7, 128.6, 128.6, 128.4, 126.1, 76.3, 70.8, 61.6, 28.2, 25.0, 14.2. $^{31}$P NMR (202 MHz, $CDCl_3$) δ -4.7.

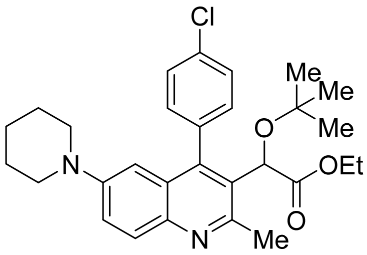

**Ethyl 2-(*tert*-butoxy)-2-(4-(4-chlorophenyl)-2-methyl-6-(piperidin-1-yl)quinolin-3-yl)acetate:** To a tall glass vial, ethyl 2-(6-bromo-4-(4-chlorophenyl)-2-methylquinolin-3-yl)-2-(*tert*-butoxy)acetate (176 mg, 0.360 mmol), piperidine (40 µL, 0.431 mmol), JohnPhos (5.4 mg, 0.018 mmol), $Pd(OAc)_2$ (2 mg, 0.009 mmol), $Cs_2CO_3$ (235 mg, 0.72 mmol) were dissolved in 1,4 dioxane was purged with nitrogen for 5 min and was capped. The reaction was stirred overnight at 100 °C. The solids were removed by filtration over Celite and washed with ethyl acetate. The filtrate was condensed *in vacuo*.

Flash chromatography was performed using (0 – 15%) ethyl acetate in hexane eluent to give yellow oil (119 mg, 69%). $^1$H NMR (500 MHz, $CDCl_3$) δ 7.89 (d, *J* = 9.3 Hz, 1H), 7.53 – 7.48 (m, 2H), 7.47 – 7.40 (m, 2H), 7.27 (dd, *J* = 8.4, 2.0 Hz, 1H), 6.46 (d, *J* = 2.7 Hz, 1H), 5.02 (s, 1H), 4.16 (m, 2H), 3.05 – 3.02 (m, 4H), 2.76 (s, 3H), 1.67 – 1.63 (m, 4H), 1.55 – 1.50 (m, 2H), 1.22 – 1.19 (m, 2H), 0.97 (s, 9H). $^{13}$C NMR (126 MHz, $CDCl_3$) δ 172.5, 155.9, 149.9, 143.9, 142.1, 135.3, 134.3, 132.4, 131.1, 129.6, 129.1, 128.9, 128.2, 127.0, 122.9, 108.3, 76.0, 70.9, 61.3, 50.6, 29.7, 28.1, 24.4, 24.2, 14.1.

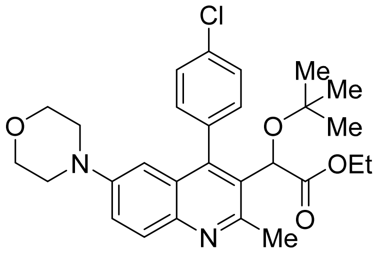


**Ethyl 2-(*tert*-butoxy)-2-(4-(4-chlorophenyl)-2-methyl-6-morpholinoquinolin-3-yl)acetate:** To a tall glass vial, ethyl 2-(*tert*-butoxy)-2-(4-(4-chlorophenyl)-6-iodo-2-methylquinolin-3-yl)acetate (125 mg, 0.232 mmol), morpholine (24 μL, 0.279 mmol), JohnPhos (3.5 mg, 0.012 mmol), $Pd(OAc)_2$ (1.3 mg, 0.006 mmol), $Cs_2CO_3$ (151 mg, 0.464 mmol) were dissolved in 1,4 dioxane was purged with nitrogen for 5 min and was capped. The reaction was stirred overnight at 100 °C. The solids were removed by filtration over Celite and washed with ethyl acetate. The filtrate was condensed *in vacuo*. Flash chromatography was performed using hexane: ethyl acetate (3:5) eluent to give red solid (46 mg, 40%). $^1$H NMR (500 MHz, $CDCl_3$) δ 7.93 (d, *J* = 9.2 Hz, 1H), 7.54 – 7.49 (m, 2H), 7.46 – 7.39 (m, 2H), 7.29 – 7.26 (m, 1H), 6.47 (d, *J* = 2.7 Hz, 1H), 5.03 (s, 1H), 4.16 (dddd, *J* = 17.9, 10.8, 7.1, 3.7 Hz, 2H), 3.83 – 3.80 (m, 4H), 3.06 – 3.03 (m, 4H), 2.77 (s, 3H), 1.22 (t, *J* = 7.1 Hz, 3H), 0.97 (s, 9H). $^{13}$C NMR (126 MHz, $CDCl_3$) δ 172.5, 156.6, 149.0, 144.1, 142.6, 135.2, 134.5, 132.4, 131.1, 130.0, 129.6, 129.1, 128.4, 127.0, 121.6, 108.2, 76.1, 70.9, 66.9, 61.5, 49.4, 28.2, 24.6, 14.2.

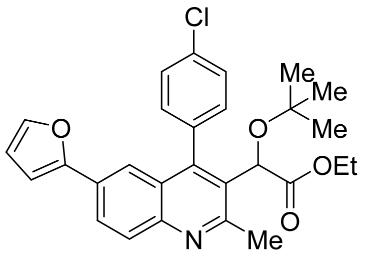


**Ethyl 2-(*tert*-butoxy)-2-(4-(4-chlorophenyl)-6-(furan-2-yl)-2-methylquinolin-3-yl)acetate:** To a tall glass vial, ethyl 2-(*tert*-butoxy)-2-(4-(4-chlorophenyl)-6-iodo-2-methylquinolin-3-yl)acetate (82 mg, 0.152 mmol), $Na_2CO_3$ (32 mg, 0.305 mmol), tetrakis(triphenylphosphine)palladium (9 mg, 0.008 mmol), 2-furanylboronic acid (26 mg, 0.229 mmol) in the mixture of 1,4-dioxane: $H_2O$ (2 mL:1 mL) was purged with nitrogen for 5 min and was capped. The reaction was stirred overnight at 80 °C. The solids were removed by filtration over Celite and washed with ethyl acetate. The filtrate was condensed *in vacuo*. Flash chromatography was performed using hexane: ethyl acetate (5:1) eluent to give light yellow oil (61 mg, 84%). $^1$H NMR (500 MHz, $CDCl_3$) δ 8.03 (d, *J* = 8.7 Hz, 1H), 7.94 (dd, *J* = 8.8, 1.9 Hz, 1H), 7.58 – 7.52 (m, 3H), 7.50 – 7.45 (m, 1H), 7.44 (dd, *J* = 1.9, 0.7 Hz, 1H), 7.33 – 7.27 (m, 1H), 6.63 (dd, *J* = 3.3, 0.8 Hz, 1H), 6.47 – 6.42 (m, 1H), 5.07 (s, 1H), 4.26 – 4.11 (m, 2H), 2.83 (s, 3H), 1.23 (t, *J* = 7.1 Hz, 3H), 0.99 (s, 9H). $^{13}$C NMR (126 MHz, $CDCl_3$) δ 172.4, 159.3, 153.4, 142.8, 134.9, 134.4, 132.4, 131.3, 130.4, 129.1, 128.8, 128.7, 128.5, 126.4, 126.2, 120.4, 112.0, 106.4, 76.3, 70.8, 61.6, 28.2, 24.6, 14.2.

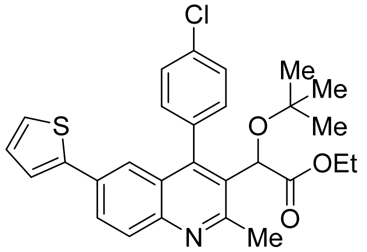


**Ethyl 2-(*tert*-butoxy)-2-(4-(4-chlorophenyl)-2-methyl-6-(thiophen-2-yl)quinolin-3-yl)acetate:** To a tall glass vial, ethyl 2-(6-bromo-4-(4-chlorophenyl)-2-methylquinolin-3-yl)-2-(*tert*-butoxy)acetate (244 mg, 0.489 mmol), $Na_2CO_3$ (104 mg, 0.978 mmol), tetrakis(triphenylphosphine)palladium (28 mg, 0.025 mmol), 2-thienylboronic acid (105 mg, 0.747 mmol) in the mixture of 1,4-dioxane: $H_2O$ (2 mL:1 mL) was purged with nitrogen for 5 min and was capped. The reaction was stirred overnight at 60 °C. The solids were removed by filtration over Celite and washed with ethyl acetate. The filtrate was condensed *in vacuo*. Flash chromatography was performed using hexane: ethyl acetate (5:1) eluent to give light yellow oil (153 mg, 63%). $^1$H NMR (500 MHz, $CDCl_3$) δ 8.05 (d, *J* = 8.7 Hz, 1H), 7.92 (dd, *J* = 8.7, 2.0 Hz, 1H), 7.57 – 7.51 (m, 2H), 7.50 – 7.45 (m, 2H), 7.33 – 7.29 (m, 1H), 7.26 (dd, *J* = 5.1, 1.2 Hz, 1H), 7.23 (dd, *J* = 3.6, 1.2 Hz, 1H), 7.05 (dd, *J* = 5.0, 3.6 Hz, 1H), 5.08 (s, 1H), 4.24 – 4.10 (m, 2H), 2.84 (s, 3H), 1.23 (t, *J* = 7.1 Hz, 3H), 0.99 (s, 9H). $^{13}$C NMR (126 MHz, $CDCl_3$) δ 172.4, 159.5, 146.3, 145.3, 143.8, 134.9, 134.5, 132.4, 132.2, 131.3, 130.4, 130.3, 129.3, 129.1, 128.4, 128.3, 128.1, 126.4, 125.6, 124.0, 122.7, 76.3, 70.8, 61.6, 28.2, 24.9, 14.3.

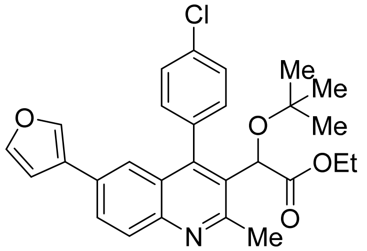


**Ethyl 2-(*tert*-butoxy)-2-(4-(4-chlorophenyl)-6-(furan-3-yl)-2-methylquinolin-3-yl)acetate:** To a tall glass vial, ethyl 2-(6-bromo-4-(4-chlorophenyl)-2-methylquinolin-3-yl)-2-(*tert*-butoxy)acetate (196 mg, 0.400 mmol), $Na_2CO_3$ (85 mg, 0.800 mmol), tetrakis(triphenylphosphine)palladium (23 mg, 0.02 mmol), 3-furanylboronic acid (67 mg, 0.600 mmol) in the mixture of 1,4-dioxane: $H_2O$ (2 mL:1 mL) was purged with nitrogen for 5 min and was capped. The reaction was stirred overnight at 80 °C. The solids were removed by filtration over Celite and washed with ethyl acetate. The filtrate was condensed *in vacuo*. Flash chromatography was performed using hexane: ethyl acetate (5:1) eluent to give light yellow oil (158 mg, 88%). $^1$H NMR (500 MHz, $CDCl_3$) δ 8.04 (dd, *J* = 8.7, 0.6 Hz, 1H), 7.79 (dd, *J* = 8.7, 2.0 Hz, 1H), 7.68 (dd, *J* = 1.6, 0.9 Hz, 1H), 7.56 – 7.52 (m, 2H), 7.49 – 7.46 (m, 1H), 7.42 (t, *J* = 1.7 Hz, 1H), 7.33 – 7.27 (m, 2H), 6.56 (dd, *J* = 1.9, 0.9 Hz, 1H), 5.08 (s, 1H), 4.18 (qq, *J* = 10.8, 7.1 Hz, 2H), 2.83 (s, 3H), 1.22 (t, *J* = 7.1 Hz, 3H), 0.99 (s, 9H). $^{13}$C NMR (126 MHz, $CDCl_3$) δ 172.5, 159.2, 146.1, 145.2, 144.0, 139.1, 134.8, 134.6, 132.4, 131.3, 130.2, 130.2, 129.3, 129.0, 128.4, 128.1, 126.4, 126.1, 122.4, 108.9, 76.2, 70.9, 61.6, 28.2, 24.9, 14.2.

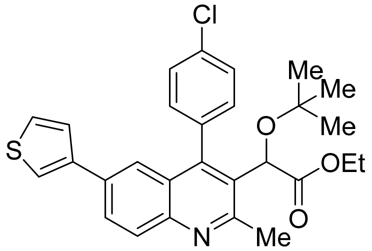


**Ethyl 2-(*tert*-butoxy)-2-(4-(4-chlorophenyl)-2-methyl-6-(thiophen-3-yl)quinolin-3-yl)acetate:** To a tall glass vial, ethyl 2-(*tert*-butoxy)-2-(4-(4-chlorophenyl)-6-iodo-2-methylquinolin-3-yl)acetate (102 mg, 0.190 mmol), $Na_2CO_3$ (40 mg, 0.380 mmol), tetrakis(triphenylphosphine)palladium (11 mg, 0.010 mmol), 3-thienylboronic acid (37 mg, 0.285 mmol) in the mixture of 1,4-dioxane: $H_2O$ (2 mL:1 mL) was purged with nitrogen for 5 min and was capped. The reaction was stirred overnight at 80 °C. The solids were removed by filtration over Celite and washed with ethyl acetate. The filtrate was condensed *in vacuo*. Flash chromatography was performed using hexane: ethyl acetate (5:1) eluent to give light yellow oil (74 mg, 78%). $^1$H NMR (500 MHz, $CDCl_3$) δ 8.11 – 8.04 (m, 1H), 7.92 (dd, *J* = 8.7, 2.0 Hz, 1H), 7.59 – 7.50 (m, 2H), 7.54 – 7.45 (m, 1H), 7.44 (dd, *J* = 2.0, 0.6 Hz, 1H), 7.40 (dd, *J* = 2.9, 1.4 Hz, 1H), 7.36 (dd, *J* = 5.0, 3.0 Hz, 1H), 7.32 – 7.26 (m, 2H), 5.08 (s, 1H), 4.19 (qq, *J* = 10.9, 7.1 Hz, 2H), 2.84 (s, 3H), 1.23 (t, *J* = 7.1 Hz, 3H), 0.99 (s, 9H). $^{13}$C NMR (126 MHz, $CDCl_3$) δ 172.5, 159.3, 146.1, 145.4, 141.7, 134.8, 134.6, 133.6, 132.4, 131.3, 130.2, 129.2, 129.0, 128.7, 128.4, 126.7, 126.5, 126.4, 123.2, 121.2, 76.3, 70.9, 61.6, 28.2, 24.9, 14.3.

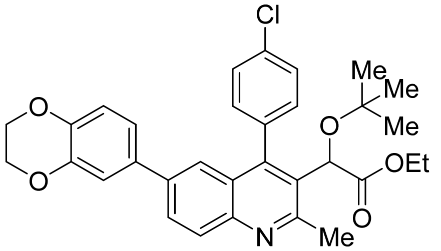


**Ethyl 2-(*tert*-butoxy)-2-(4-(4-chlorophenyl)-6-(2,3-dihydrobenzo[b][1,4]dioxin-6-yl)-2-methylquinolin-3-yl)acetate:** To a tall glass vial, ethyl 2-(*tert*-butoxy)-2-(4-(4-chlorophenyl)-6-iodo-2-methylquinolin-3-yl)acetate (190 mg, 0.353 mmol), $Na_2CO_3$ (75 mg, 0.706 mmol), tetrakis(triphenylphosphine)palladium (21 mg, 0.018 mmol), 1,4-benzodioxane-6-boronic acid (95 mg, 0.530 mmol) in the mixture of 1,4-dioxane: $H_2O$ (2 mL:1 mL) was purged with nitrogen for 5 min and was capped. The reaction was stirred overnight at 80 °C. The solids were removed by filtration over Celite and washed with ethyl acetate. The filtrate was condensed *in vacuo*. Flash chromatography was performed using hexane: ethyl acetate (4:1) eluent to give light yellow oil (121 mg, 63%). $^1$H NMR (500 MHz, $CDCl_3$) δ 8.07 (d, *J* = 8.7 Hz, 1H), 7.85 (dd, *J* = 8.7, 2.0 Hz, 1H), 7.55 – 7.50 (m, 2H), 7.47 (dd, *J* = 8.4, 2.2 Hz, 1H), 7.37 (d, *J* = 2.0 Hz, 1H), 7.30 (dd, *J* = 8.4, 2.2 Hz, 1H), 7.02 – 6.96 (m, 2H), 6.90 (d, *J* = 8.2 Hz, 1H), 5.08 (s, 1H), 4.27 (s, 4H), 4.23 – 4.13 (m, 2H), 2.84 (s, 3H), 1.23 (t, *J* = 7.1 Hz, 3H), 0.99 (s, 9H). $^{13}$C NMR (126 MHz, $CDCl_3$) δ 172.5, 159.3, 146.0, 145.4, 143.8, 143.5, 138.4, 134.8, 134.7, 134.2, 132.4, 131.3, 130.2, 129.1, 128.5, 126.3, 123.6, 120.6, 117.8, 116.2, 76.2, 70.9, 64.5, 61.6, 28.2, 24.9, 14.3.

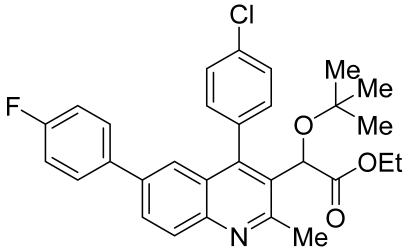


**Ethyl 2-(*tert*-butoxy)-2-(4-(4-chlorophenyl)-6-(4-fluorophenyl)-2-methylquinolin-3-yl)acetate:** To a tall glass vial, ethyl 2-(6-bromo-4-(4-chlorophenyl)-2-methylquinolin-3-yl)-2-(*tert*-butoxy)acetate (156 mg, 0.318 mmol), $Na_2CO_3$ (67 mg, 0.636 mmol), tetrakis(triphenylphosphine)palladium (19 mg, 0.016 mmol), 4-fluorophenylboronic acid (67 mg, 0.477 mmol) in the mixture of 1,4-dioxane: $H_2O$ (2 mL:1 mL) was purged with nitrogen for 5 min and was capped. The reaction was stirred overnight at 80 °C. The solids were removed by filtration over Celite and washed with ethyl acetate. The filtrate was condensed *in vacuo*. Flash

chromatography was performed using hexane: ethyl acetate (4:1) eluent to give light yellow oil (159 mg, 99%). $^{1}$H NMR (500 MHz, $CDCl_3$) δ 8.13 (d, *J* = 8.7 Hz, 1H), 7.86 (dd, *J* = 8.7, 2.1 Hz, 1H), 7.55 (ddd, *J* = 8.3, 5.9, 2.1 Hz, 2H), 7.51 – 7.39 (m, 4H), 7.31 (dd, *J* = 8.4, 2.1 Hz, 1H), 7.12 – 7.06 (m, 2H), 5.10 (s, 1H), 4.25 – 4.12 (m, 2H), 2.86 (s, 3H), 1.24 (t, *J* = 7.1 Hz, 3H), 1.00 (s, 9H). $^{13}$C NMR (126 MHz, $CDCl_3$) δ 172.4, 163.7, 161.7, 159.6, 145.9, 138.1, 136.7, 135.0, 134.4, 132.3, 131.2, 130.4, 129.3, 129.1, 129.1, 128.9, 128.6, 126.4, 124.1, 116.0, 115.9, 76.4, 70.8, 61.7, 28.2, 24.7, 14.3. $^{19}$F NMR (471 MHz, $CDCl_3$) δ -114.8.

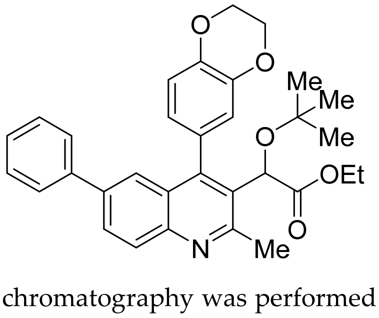


**Ethyl 2-(*tert*-butoxy)-2-(4-(2,3-dihydrobenzo[*b*][1,4]dioxin-6-yl)-2-methyl-6-phenylquinolin-3-yl)acetate:** To a tall glass vial, ethyl 2-(6-bromo-4-(2,3-dihydrobenzo[*b*][1,4]dioxin-6-yl)-2-methylquinolin-3-yl)-2-(*tert*-butoxy)acetate (140 mg, 0.271 mmol), $Na_2CO_3$ (58 mg, 0.512 mmol), tetrakis(triphenylphosphine)palladium (16 mg, 0.014 mmol), phenylboronic acid (50 mg, 0.407 mmol) in the mixture of 1,4-dioxane: $H_2O$ (2 mL:1 mL) was purged with nitrogen for 5 min and was capped. The reaction was stirred overnight at 80 °C. The solids were removed by filtration over Celite and washed with ethyl acetate. The filtrate was condensed *in vacuo*. Flash chromatography was performed using hexane: ethyl acetate (2:1) eluent to give light yellow oil (103 mg, 74%). $^{1}$H NMR (500 MHz, $CDCl_3$) δ 8.13 (d, *J* = 8.7 Hz, 1H), 7.90 (ddd, *J* = 8.7, 3.2, 2.1 Hz, 1H), 7.61 (ddd, *J* = 4.0, 2.1, 0.6 Hz, 1H), 7.54 – 7.50 (m, 2H), 7.45 – 7.37 (m, 2H), 7.35 – 7.28 (m, 1H), 7.06 – 6.96 (m, 2H), 6.88 – 6.81 (m, 1H), 5.25 (d, *J* = 8.7 Hz, 1H), 4.38 – 4.27 (m, 4H), 4.25 – 4.14 (m, 2H), 2.85 (s, 3H), 1.25 – 1.22 (m, 3H), 1.01 (d, *J* = 7.3 Hz, 9H). $^{13}$C NMR (126 MHz, $CDCl_3$) (isomer) δ 172.7 (172.6), 159.5, 146.7 (146.6), 146.0, 143.9 (143.8), 143.7 (143.2), 141.0 (140.9), 138.8 (138.7), 130.5 (130.5), 129.2 (129.1), 129.0 (129.0), 128.9 (128.8), 127.6 (127.5), 126.8 (126.8), 124.8 (124.7), 124.1, 123.2, 120.0, 118.9, 117.5 (117.0), 76.2 (76.2), 70.9 (70.9), 64.6 (64.4), 61.6 (61.4), 60.5, 28.2, 24.7 (24.7), 14.3 (14.2).

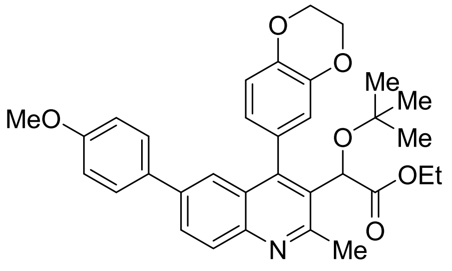


**Ethyl 2-(*tert*-butoxy)-2-(4-(2,3-dihydrobenzo[*b*][1,4]dioxin-6-yl)-6-(4-methoxyphenyl)-2-methylquinolin-3-yl)acetate:** To a tall glass vial, ethyl 2-(6-bromo-4-(2,3-dihydrobenzo[*b*][1,4]dioxin-6-yl)-2-methylquinolin-3-yl)-2-(*tert*-butoxy)acetate (105 mg, 0.204 mmol), $Na_2CO_3$ (43 mg, 0.408 mmol), tetrakis(triphenylphosphine)palladium (12 mg, 0.010 mmol), 4-methoxyphenylboronic acid (47 mg, 0.204 mmol) in the mixture of 1,4-dioxane: $H_2O$ (2 mL:1 mL) was purged with nitrogen for 5 min and was capped. The reaction was stirred overnight at 80 °C. The solids were removed by filtration over Celite and washed with ethyl acetate. The filtrate was condensed *in vacuo*. Flash chromatography was performed using hexane: ethyl acetate (1:1) eluent to give light yellow oil (129 mg, 99%). $^{1}$H NMR (500 MHz, $CDCl_3$) δ 8.06 (dt, *J* = 8.7, 0.7 Hz, 1H), 7.86 (ddd, *J* = 8.7, 2.9, 2.1 Hz, 1H), 7.54 (ddd, *J* = 3.6, 2.1, 0.6 Hz, 1H), 7.49 – 7.42 (m, 2H), 7.05 – 6.97 (m, 2H), 6.97 – 6.93 (m, 2H), 6.88 – 6.81 (m, 1H), 5.23 (d, *J* = 9.2 Hz, 1H), 4.41 – 4.29 (m, 4H), 4.26 – 4.09 (m, 2H), 3.83 (d, *J* = 1.8 Hz, 3H), 2.82 (s, 3H), 1.25 – 1.21 (m, 3H), 1.01 (d, *J* = 7.5 Hz, 9H). $^{13}$C NMR (126 MHz, $CDCl_3$) (isomer) δ 172.8 (172.7), 159.4 (159.2), 146.3 (146.3), 146.0, 143.8 (143.7), 143.6 (143.2), 138.3 (138.2), 133.5 (133.4), 130.4 (130.3), 129.2 (129.2), 128.9 (128.8), 128.7 (128.6), 126.8 (126.8), 124.1 (124.0), 124.0, 123.2, 120.1, 119.0, 117.5 (117.0), 114.4, 76.1 (76.1), 70.9 (70.9), 64.6 (64.5), 55.5, 28.2, 24.9 (24.8), 14.3 (14.2).

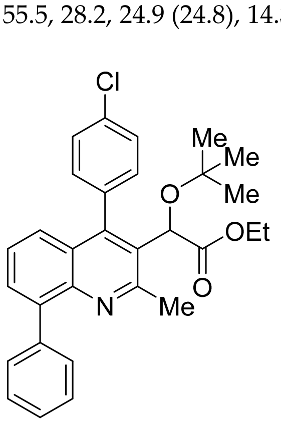


**Ethyl 2-(*tert*-butoxy)-2-(4-(4-chlorophenyl)-2-methyl-8-phenylquinolin-3-yl)acetate:** To a tall glass vial, ethyl 2-(8-bromo-4-(4-chlorophenyl)-2-methylquinolin-3-yl)-2-(*tert*-butoxy)acetate (112 mg, 0.225 mmol), $Na_2CO_3$ (48 mg, 0.450 mmol), tetrakis(triphenylphosphine)palladium (13 mg, 0.011 mmol), phenylboronic acid (41 mg, 0.338 mmol) in the mixture of 1,4-dioxane: $H_2O$ (2 mL:1 mL) was purged with nitrogen for 5 min and was capped. The reaction was stirred overnight at 80 °C. The solids were removed by filtration over Celite and washed with ethyl acetate. The filtrate was condensed *in vacuo*. Flash chromatography was performed using (0 – 15%) ethyl acetate in hexane eluent to give light yellow oil (98 mg, 89%). $^{1}$H NMR (500 MHz, $CDCl_3$) δ 7.84 – 7.80 (m, 2H), 7.71 (dd, *J* = 7.1, 1.4 Hz, 1H), 7.58 – 7.53 (m, 2H), 7.52 – 7.47 (m, 3H), 7.45 – 7.39 (m, 2H), 7.34 – 7.28 (m, 2H), 5.10 (s, 1H), 4.28 – 4.08 (m, 2H), 2.80 (s, 3H), 1.24 (t, *J* = 7.1 Hz, 3H), 1.02 (s, 9H). $^{13}$C NMR (126 MHz, $CDCl_3$) δ 172.6, 159.0, 145.3, 144.4, 140.0, 139.6, 135.2, 134.6, 132.5, 131.4, 131.2, 130.3, 129.4, 128.9, 128.4, 127.8, 127.3, 126.6, 126.1, 125.8, 76.2, 71.0, 61.6, 28.2, 25.4, 14.3.

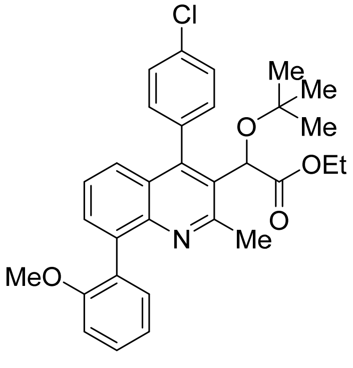

**Ethyl 2-(*tert*-butoxy)-2-(4-(4-chlorophenyl)-8-(2-methoxyphenyl)-2-methylquinolin-3-yl)acetate:** To a tall glass vial, ethyl 2-(8-bromo-4-(4-chlorophenyl)-2-methylquinolin-3-yl)-2-(*tert*-butoxy)acetate (98 mg, 0.200 mmol), $Na_2CO_3$ (42 mg, 0.400 mmol), tetrakis(triphenylphosphine)palladium (12 mg, 0.010 mmol), 2-methoxyphenylboronic acid (46 mg, 0.300 mmol) in the mixture of 1,4-dioxane: $H_2O$ (2 mL:1 mL) was purged with nitrogen for 5 min and was capped. The reaction was stirred overnight at 80 °C. The solids were removed by filtration over Celite and washed with ethyl acetate. The filtrate was condensed *in vacuo*. Flash chromatography was performed using (0 – 15%) ethyl acetate in hexane eluent to give light yellow solid (28 mg, 25%). $^1$H NMR (500 MHz, $CDCl_3$) δ 7.66 (dd, *J* = 7.1, 1.5 Hz, 1H), 7.54 – 7.51 (m, 2H), 7.48 – 7.45 (m, 1H), 7.44 – 7.40 (m, 2H), 7.40 – 7.37 (m, 1H), 7.33 – 7.29 (m, 1H), 7.27 (dd, *J* = 8.4, 1.5 Hz, 1H), 7.10 – 7.06 (m, 2H), 5.06 (s, 1H), 4.22 – 4.09 (m, 2H), 3.77 (s, 3H), 2.69 (s, 3H), 1.21 (t, *J* = 7.1 Hz, 3H), 0.98 (s, 9H). $^{13}$C NMR (126 MHz, $CDCl_3$) δ 172.6, 158.6, 157.7, 145.0, 144.9, 137.4, 135.3, 134.5, 132.6, 132.5, 131.4, 131.0, 129.2, 129.2, 128.8, 128.8, 128.3, 126.3, 126.2, 125.4, 120.4, 111.6, 76.1, 71.0, 61.5, 56.0, 28.2, 25.3, 14.3.

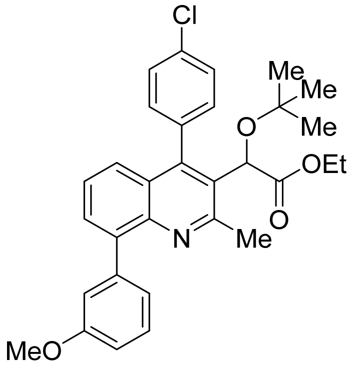

**Ethyl 2-(*tert*-butoxy)-2-(4-(4-chlorophenyl)-8-(3-methoxyphenyl)-2-methylquinolin-3-yl)acetate:** To a tall glass vial, ethyl 2-(8-bromo-4-(4-chlorophenyl)-2-methylquinolin-3-yl)-2-(*tert*-butoxy)acetate (105 mg, 0.214 mmol), $Na_2CO_3$ (45 mg, 0.428 mmol), tetrakis(triphenylphosphine)palladium (13 mg, 0.011 mmol), 3-methoxyphenylboronic acid (49 mg, 0.328 mmol) in the mixture of 1,4-dioxane: $H_2O$ (2 mL:1 mL) was purged with nitrogen for 5 min and was capped. The reaction was stirred overnight at 80 °C. The solids were removed by filtration over Celite and washed with ethyl acetate. The filtrate was condensed *in vacuo*. Flash chromatography was performed using (0 – 15%) ethyl acetate in hexane eluent to give light yellow solid (101 mg, 91%). $^1$H NMR (500 MHz, $CDCl_3$) δ 7.72 (dd, *J* = 7.1, 1.5 Hz, 1H), 7.57 – 7.51 (m, 2H), 7.51 – 7.47 (m, 1H), 7.45 – 7.35 (m, 4H), 7.33 – 7.27 (m, 2H), 6.97 (ddd, *J* = 7.9, 2.6, 1.4 Hz, 1H), 5.09 (s, 1H), 4.24 – 4.11 (m, 2H), 3.89 (s, 3H), 2.80 (s, 3H), 1.23 (t, *J* = 7.1 Hz, 3H), 1.01 (s, 9H). $^{13}$C NMR (126 MHz, $CDCl_3$) δ 172.6, 159.1, 159.0, 145.3, 144.3, 140.9, 139.7, 135.2, 134.6, 132.4, 131.4, 130.3, 129.5, 128.9, 128.8, 128.4, 126.6, 126.2, 125.7, 123.7, 116.9, 113.2, 76.2, 70.9, 61.6, 55.4, 28.2, 25.4, 14.3.

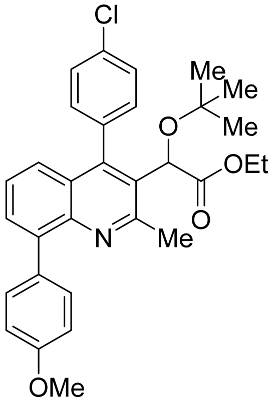

**Ethyl 2-(*tert*-butoxy)-2-(4-(4-chlorophenyl)-8-(4-methoxyphenyl)-2-methylquinolin-3-yl)acetate**: To a tall glass vial, ethyl 2-(8-bromo-4-(4-chlorophenyl)-2-methylquinolin-3-yl)-2-(*tert*-butoxy)acetate (107 mg, 0.219 mmol), $Na_2CO_3$ (46 mg, 0.438 mmol), tetrakis(triphenylphosphine)palladium (13 mg, 0.011 mmol), 4-methoxyphenylboronic acid (50 mg, 0.328 mmol) in the mixture of 1,4-dioxane: $H_2O$ (2 mL:1 mL) was purged with nitrogen for 5 min and was capped. The reaction was stirred overnight at 80 °C. The solids were removed by filtration over Celite and washed with ethyl acetate. The filtrate was condensed *in vacuo*. Flash chromatography was performed using (0 – 15%) ethyl acetate in hexane eluent to give light yellow solid (41 mg, 36%). $^1$H NMR (500 MHz, $CDCl_3$) δ 7.81 – 7.74 (m, 2H), 7.68 (dd, *J* = 7.1, 1.4 Hz, 1H), 7.57 – 7.50 (m, 2H), 7.50 – 7.44 (m, 1H), 7.39 (dd, *J* = 8.4, 7.1 Hz, 1H), 7.33 – 7.27 (m, 1H), 7.24 (dd, *J* = 8.5, 1.4 Hz, 1H), 7.06 – 7.00 (m, 2H), 5.08 (s, 1H), 4.23 – 4.10 (m, 2H), 3.89 (s, 3H), 2.79 (s, 3H), 1.22 (t, *J* = 7.1 Hz, 3H), 1.00 (s, 9H). $^{13}$C NMR (126 MHz, $CDCl_3$) δ 172.6, 159.1, 158.7, 145.2, 144.4, 139.5, 135.3, 134.6, 132.4, 132.3, 132.0, 131.4, 129.8, 129.3, 128.9, 128.4, 126.7, 125.8, 125.6, 113.4, 76.2, 70.9, 61.5, 55.4, 28.2, 25.4, 14.3.

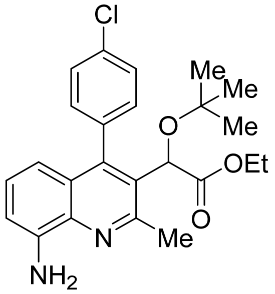

**Ethyl 2-(8-amino-4-(4-chlorophenyl)-2-methylquinolin-3-yl)-2-(*tert*-butoxy)acetate:** To a tall glass vial, ethyl 2-(8-bromo-4-(4-chlorophenyl)-2-methylquinolin-3-yl)-2-(*tert*-butoxy)acetate (106 mg, 0.216 mmol), L-Proline (32 mg, 0.281 mmol) was dissolved in DMSO (750 μL) and purged with argon gas for 5 min. $NaN_3$ (28 mg, 0.432 mmol) and CuI (41 mg, 0.216 mmol) were added. The solution was allowed to be purged with argon for additional 10 min. The vial was capped and heated at 100 °C for 3 h. After the reaction complete, the solution was quenched with saturated solution of $NH_4Cl$ (5 mL) and water (10 mL). The solution was extracted with ethyl acetate (3x20 mL). The combined organic layers were dried with anhydrous sodium sulfate and condensed *in vacuo* to give a brown solid (89 mg, 97%). $^1$H NMR (500 MHz, $CDCl_3$) δ 7.49 (td, *J* = 5.7, 2.7 Hz, 2H), 7.42 (dd, *J* = 8.5, 2.2 Hz, 1H), 7.29 – 7.24 (m, 1H), 7.14 (t, *J* = 7.9 Hz, 1H), 6.88 (d, *J* = 7.4 Hz, 1H), 6.56 (d, *J* = 8.4 Hz, 1H), 5.06 (s, 1H), 4.24 – 4.11 (m, 2H), 2.82 (s, 3H), 1.22 (t, *J* = 7.1 Hz, 3H), 0.99 (s, 9H). $^{13}$C NMR (126 MHz, $CDCl_3$) δ 172.6, 156.4, 145.4, 135.3, 134.4, 133.0, 132.3, 131.3, 128.7, 128.2, 126.8, 120.2, 114.9, 110.2, 76.1, 70.9, 61.5, 41.0, 28.2, 14.2.

**General Procedure for the Preparation of 6- and 8- Aryl Quinoline Acetic Acids**

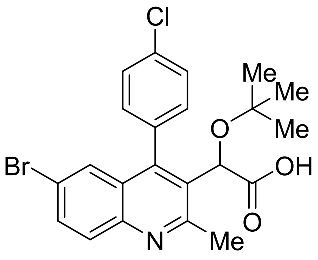

**2-(6-Bromo-4-(4-chlorophenyl)-2-methylquinolin-3-yl)-2-(*tert*-butoxy)acetic acid (ALLINI-2):** To a medium vial, ethyl 2-(6-bromo-4-(4-chlorophenyl)-2-methylquinolin-3-yl)-2-(*tert*-butoxy)acetate (180 mg, 0.335 mmol), NaOH (54 mg, 1.339 mmol) was dissolved in ethanol (2 mL) and $H_2O$ (1 mL). The vial was purged with argon, capped, and refluxed at 80 °C for 16 h. After the reaction was completed, the solution was condensed *in vacuo*. The solid was washed with methylene chloride on filter paper. The solid then was dissolved in $H_2O$ and acidified with 1M HCl to pH 1. The aqueous solution was extracted with ethyl acetate (3x50 mL). The combined organic layers were dried with anhydrous sodium sulfate, and condensed *in vacuo* to give white solid (123 mg, 77%). $^1$H NMR (500 MHz, DMSO-$d_6$) δ 7.87 (d, *J* = 8.9 Hz, 1H), 7.79 (dd, *J* = 8.9, 2.2 Hz, 1H), 7.66 (ddd, *J* = 20.7, 8.2, 2.3 Hz, 2H), 7.43 (ddd, *J* = 22.6, 8.2, 2.2 Hz, 2H), 7.30 (d, *J* = 2.2 Hz, 1H), 4.95 (s, 1H), 2.70 (s, 3H), 0.86 (s, 9H). $^{13}$C NMR (126 MHz, DMSO-$d_6$) δ 173.6, 159.9, 145.3, 144.2, 134.4, 134.1, 133.1, 132.7, 132.1, 131.4, 131.2, 129.5, 129.1, 128.3, 127.4, 119.9, 76.0, 70.4, 28.3, 25.1.

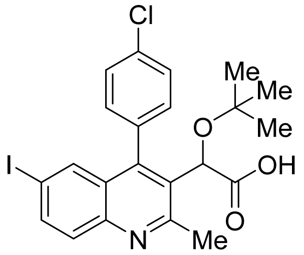

**2-(*Tert*-butoxy)-2-(4-(4-chlorophenyl)-6-iodo-2-methylquinolin-3-yl)acetic acid (8a):** To a medium vial, ethyl 2-(*tert*-butoxy)-2-(4-(4-chlorophenyl)-6-iodo-2-methylquinolin-3-yl)acetate (180 mg, 0.335 mmol), NaOH (54 mg, 1.339 mmol) was dissolved in ethanol (2 mL) and $H_2O$ (1 mL). The vial was purged with argon, capped, and refluxed at 80 °C for 16 h. After the reaction was completed, the solution was condensed *in vacuo*. The solid was washed with methylene chloride on filter paper. The solid then was dissolved in $H_2O$ and acidified with 1M HCl to pH 1. The aqueous solution was extracted with ethyl acetate (3x50 mL). The combined organic layers were dried with anhydrous sodium sulfate and condensed *in vacuo* to give white solid (115 mg, 68%). $^1$H NMR (500 MHz, DMSO-$d_6$) δ 7.95 (dd, *J* = 8.8, 2.0 Hz, 1H), 7.72 (d, *J* = 8.8 Hz, 1H), 7.70 – 7.64 (m, 2H), 7.51 (d, *J* = 1.9 Hz, 1H), 7.44 – 7.38 (m, 2H), 4.92 (s, 1H), 2.68 (s, 3H), 0.86 (s, 9H). $^{13}$C NMR (126 MHz, DMSO-$d_6$) δ 173.6, 159.9, 145.5, 143.9, 138.4, 134.7, 134.4, 134.1, 132.6, 132.2, 131.1, 131.0, 129.5, 129.1, 127.9, 93.2, 76.0, 70.3, 28.3, 25.1.

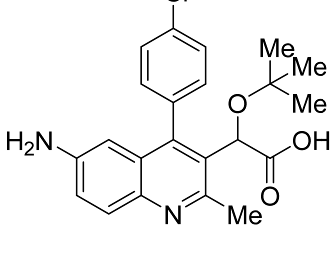

**2-(6-Amino-4-(4-chlorophenyl)-2-methylquinolin-3-yl)-2-(tert-butoxy)acetic acid (8b):** To a medium vial, ethyl 2-(6-amino-4-(4-chlorophenyl)-2-methylquinolin-3-yl)-2-(*tert*-butoxy)acetate (75 mg, 0.177 mmol), NaOH (28 mg, 0.708 mmol) was dissolved in ethanol (2 mL) and $H_2O$ (1 mL). The vial was purged with argon, capped, and refluxed at 80 °C for 16 h. After the reaction was completed, the solution was condensed *in vacuo*. The solid was re-dissolved in water and extracted with ethyl acetate (20 mL). The aqueous layers were acidified to pH 1 and extracted with ethyl acetate (30 mLx3). The combined organic layers were dried with anhydrous sodium sulfate, and condensed *in vacuo* to give a brown solid.

The solid was triturated with diethyl ether to give orange solid (44 mg, 94%). $^1$H NMR (500 MHz, DMSO-$d_6$) δ 8.17 (d, *J* = 9.1 Hz, 1H), 7.73 (ddd, *J* = 14.8, 8.2, 2.3 Hz, 2H), 7.46 – 7.36 (m, 3H), 6.28 (d, *J* = 2.3 Hz, 1H), 4.92 (s, 1H), 2.86 (s, 3H), 0.90 (s, 9H). $^{13}$C NMR (126 MHz, DMSO-$d_6$) δ 172.8, 172.6, 134.8, 133.7, 132.0, 131.7, 131.7, 131.6, 130.0, 129.7, 129.4, 129.4, 129.4, 129.3, 128.7, 125.2, 76.6, 70.1, 28.2, 21.6.

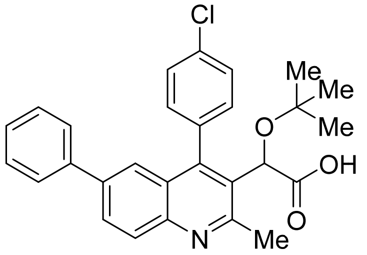


**2-(*Tert*-butoxy)-2-(4-(4-chlorophenyl)-2-methyl-6-phenylquinolin-3-yl)acetic acid (8c):** To a medium vial, ethyl 2-(*tert*-butoxy)-2-(4-(4-chlorophenyl)-2-methyl-6-phenylquinolin-3-yl)acetate (65 mg, 0.134 mmol), NaOH (22 mg, 0.536 mmol) was dissolved in ethanol (2 mL) and $H_2O$ (1 mL). The vial was purged with argon, capped, and refluxed at 80 °C for 16 h. After the reaction was completed, the solution was condensed *in vacuo*. The solid was re-dissolved in water and extracted with ethyl acetate (2x20 mL). The aqueous layer was acidified to pH 1 and extracted with ethyl acetate (3x30 mL). The combined organic layers in the second extraction were dried with anhydrous sodium sulfate, and condensed *in vacuo* to give a white solid (29 mg, 47%). $^1$H NMR (500 MHz, DMSO-$d_6$) δ 8.07 – 7.95 (m, 2H), 7.68 (ddd, *J* = 28.7, 8.0, 2.2 Hz, 2H), 7.55 – 7.45 (m, 4H), 7.45 – 7.37 (m, 3H), 7.32 (t, *J* = 7.4 Hz, 1H), 4.99 (s, 1H), 2.73 (s, 3H), 0.88 (s, 9H). $^{13}$C NMR (126 MHz, DMSO-$d_6$) δ 173.8, 159.2, 146.0, 145.2, 140.2, 138.6, 134.7, 134.1, 132.7, 132.4, 130.9, 129.7, 129.5, 129.4, 129.3, 128.9, 128.3, 127.5, 126.1, 123.9, 76.0, 70.5, 28.3, 25.0.

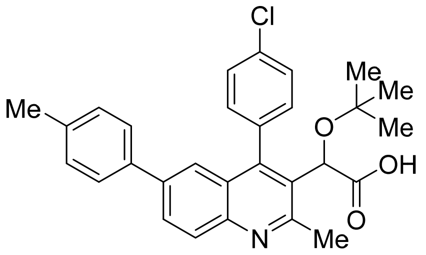


**2-(*Tert*-butoxy)-2-(4-(4-chlorophenyl)-2-methyl-6-(p-tolyl)quinolin-3-yl)acetic acid (8d):** To a medium vial, ethyl 2-(*tert*-butoxy)-2-(4-(4-chlorophenyl)-2-methyl-6-(o-tolyl)quinolin-3-yl)acetate (129 mg, 0.257 mmol), NaOH (41 mg, 1.03 mmol) was dissolved in ethanol (2 mL) and $H_2O$ (1 mL). The vial was purged with argon, capped, and refluxed at 80 °C for 16 h. After the reaction was completed, the solution was condensed *in vacuo*. The solid was re-dissolved in water and extracted with ethyl acetate (20 mL). The aqueous layers were acidified to pH 1 and extracted with ethyl acetate (3x30 mL). The combined organic layers were dried with anhydrous sodium sulfate, and condensed *in vacuo* to give a brown solid (27 mg, 22%). $^1$H NMR (500 MHz, DMSO-$d_6$) δ 8.06 – 7.94 (m, 2H), 7.71 – 7.62 (m, 2H), 7.52 – 7.44 (m, 2H), 7.41 – 7.38 (m, 2H), 7.37 – 7.35 (m, 1H), 7.24 – 7.20 (m, 2H), 4.98 (s, 1H), 2.73 (s, 3H), 2.27 (s, 3H), 0.88 (s, 9H). $^{13}$C NMR (126 MHz, DMSO-$d_6$) δ 173.7, 158.9, 138.7, 137.8, 137.2, 134.6, 134.2, 132.7, 132.4, 132.0, 130.9, 130.3, 129.5, 128.9, 127.3, 126.2, 123.6, 115.5, 76.0, 70.4, 28.3, 24.7, 21.2.

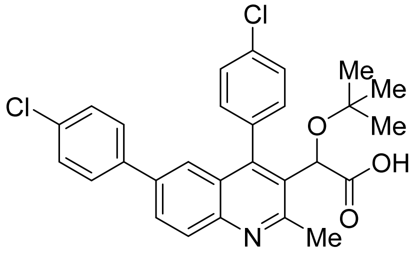


**2-(4,6-Bis(4-chlorophenyl)-2-methylquinolin-3-yl)-2-(*tert*-butoxy)acetic acid (8e):** To a medium vial, Ethyl 2-(4,6-bis(4-chlorophenyl)-2-methylquinolin-3-yl)-2-(*tert*-butoxy)acetate (60 mg, 0.115 mmol), NaOH (18 mg, 0.460 mmol) was dissolved in ethanol (2 mL) and $H_2O$ (1 mL). The vial was purged with argon, capped, and refluxed at 80 °C for 16 h. After the reaction was completed, the solution was condensed *in vacuo*. The solid was washed with methylene chloride on filter paper. The solid then was dissolved in $H_2O$ and acidified with 1M HCl to pH 1. The aqueous solution was extracted with ethyl acetate (3x50 mL). The combined organic layers were dried with anhydrous sodium sulfate, and condensed *in vacuo* to give yellow solid. The solid was triturated with diethyl ether to give tan powder (48 mg, 83%).$^1$H NMR (500 MHz, DMSO-$d_6$) δ 8.03 (dd, *J* = 8.7, 0.6 Hz, 1H), 7.99 (dd, *J* = 8.7, 2.0 Hz, 1H), 7.68 (ddd, *J* = 31.9, 8.8, 2.6 Hz, 2H), 7.56 – 7.52 (m, 2H), 7.51 – 7.46 (m, 4H), 7.38 (dd, *J* = 2.0, 0.7 Hz, 1H), 4.97 (s, 1H), 2.72 (s, 3H), 0.87 (s, 9H). $^{13}$C NMR (126 MHz, DMSO-$d_6$) δ 173.8, 159.4, 146.2, 145.2, 139.0, 137.3, 134.6, 134.2, 133.3, 132.7, 132.4, 131.0, 129.8, 129.7, 129.5, 129.3, 129.1, 129.0, 126.1, 124.0, 75.9, 70.5, 28.3, 25.1.

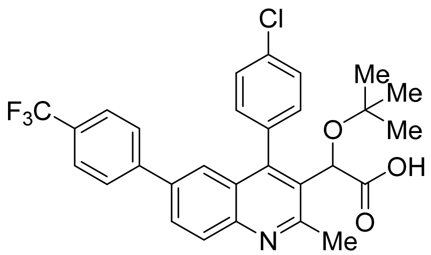


**2-(*Tert*-butoxy)-2-(4-(4-chlorophenyl)-2-methyl-6-(4-(trifluoromethyl)phenyl)quinolin-3-yl)acetic acid (8f):** To a medium vial ethyl 2-(*tert*-butoxy)-2-(4-(4-chlorophenyl)-2-methyl-6-(4-(trifluoromethyl)phenyl) quinolin-3-yl)acetate (78 mg, 0.140 mmol), NaOH (23 mg, 0.560 mmol) was dissolved in ethanol (2 mL) and $H_2O$ (1 mL). The vial was purged with argon, capped, and refluxed at 80 °C for 16 h. After the reaction was completed, the solution was condensed *in vacuo*. The solid was washed with methylene chloride on filter paper. The solid then was dissolved in $H_2O$ and acidified with 1M HCl to pH 1. The aqueous solution was extracted with ethyl acetate (3x50 mL). The combined organic layers were dried with anhydrous sodium sulfate, and condensed *in vacuo* to give yellow solid. The solid was triturated with diethyl ether to give white powder (57 mg, 77%).$^1$H NMR (500 MHz, DMSO-$d_6$) δ 8.08 – 8.01 (m, 2H), 7.76 (d, *J* = 8.4 Hz, 2H), 7.74 – 7.69 (m, 3H), 7.64 (dd, *J* = 8.1, 2.3 Hz, 1H), 7.52 – 7.44 (m, 3H), 4.99 (s, 1H), 2.73 (s, 3H), 0.87 (s, 9H). $^{13}$C NMR (126 MHz, DMSO-$d_6$) δ 173.8, 159.8, 146.4, 145.3, 144.2, 137.1, 134.5, 134.2, 132.7, 132.4, 131.1, 129.9, 129.5, 129.2, 129.0, 128.9, 128.4, 126.6 (q, *J* = 3.6 Hz), 128.6 – 123.6 (m), 126.0, 124.8, 76.0, 70.5, 28.3, 25.1. $^{19}$F NMR (471 MHz, DMSO-$d_6$) δ -60.88.

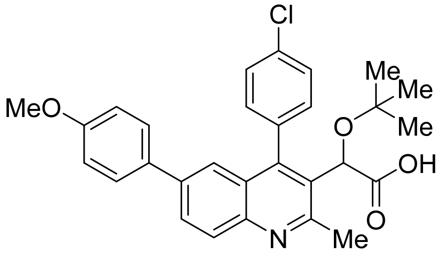


**2-(*Tert*-butoxy)-2-(4-(4-chlorophenyl)-6-(4-methoxyphenyl)-2-methylquinolin-3-yl)acetic acid (8g):** To a medium vial, ethyl 2-(*tert*-butoxy)-2-(4-(4-chlorophenyl)-6-(4-methoxyphenyl)-2-methylquinolin-3-yl)acetate (124 mg, 0.240 mmol), NaOH (40 mg, 0.960 mmol) was dissolved in ethanol (2 mL) and $H_2O$ (1 mL). The vial was purged with argon, capped, and refluxed at 80 °C for 16 h. After the reaction was completed, the solution was condensed *in vacuo*. The solid was washed with methylene chloride on filter paper. The solid then was dissolved in $H_2O$ and acidified with 1M HCl to pH 1. The aqueous solution was extracted with ethyl acetate (3x50 mL). The combined organic layers were dried with anhydrous sodium sulfate, and condensed *in vacuo* to give yellow solid. The solid was triturated with diethyl ether to give light yellow powder (59 mg, 50%).$^1$H NMR (500 MHz, $CDCl_3$) δ 8.13 (d, *J* = 8.7 Hz, 1H), 7.90 (dd, *J* = 8.7, 2.0 Hz, 1H), 7.71 (d, *J* = 8.2 Hz, 1H), 7.55 (ddd, *J* = 14.6, 8.1, 2.2 Hz, 2H), 7.46 – 7.40 (m, 3H), 7.36 – 7.31 (m, 1H), 6.97 – 6.92 (m, 2H), 5.23 (s, 1H), 3.83 (s, 3H), 2.87 (s, 3H), 1.02 (s, 9H).$^{13}$C NMR (126 MHz, $CDCl_3$) δ 207.1, 172.1, 159.6, 139.0, 135.2, 134.1, 133.0, 132.9, 131.1, 129.6, 128.5, 128.5, 123.4, 114.5, 78.2, 55.5, 31.0, 28.3, 24.7.

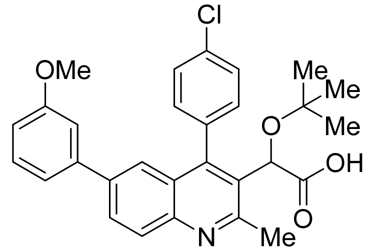


**2-(*Tert*-butoxy)-2-(4-(4-chlorophenyl)-6-(3-methoxyphenyl)-2-methylquinolin-3-yl)acetic acid (8h):** To a medium vial, ethyl 2-(*tert*-butoxy)-2-(4-(4-chlorophenyl)-6-(3-methoxyphenyl)-2-methylquinolin-3-yl)acetate (101 mg, 0.193 mmol), NaOH (32 mg, 0.80 mmol) was dissolved in ethanol (2 mL) and $H_2O$ (1 mL). The vial was purged with argon, capped, and refluxed at 80 °C for 16 h. After the reaction was completed, the solution was condensed *in vacuo*. The solid was washed with methylene chloride on filter paper. The solid then was dissolved in $H_2O$ and acidified with 1M HCl to pH 1. The aqueous solution was extracted with ethyl acetate (3x50 mL). The combined organic layers were dried with anhydrous sodium sulfate, and condensed *in vacuo* to give yellow solid. The solid was triturated with diethyl ether to give light yellow powder (78 mg, 82%).$^1$H NMR (500 MHz, DMSO-$d_6$) δ 8.01 (t, *J* = 1.5 Hz, 2H), 7.71 (dd, *J* = 8.4, 2.3 Hz, 1H), 7.66 (dd, *J* = 8.4, 2.3 Hz, 1H), 7.49 (ddt, *J* = 7.0, 4.9, 2.1 Hz, 2H), 7.37 (t, *J* = 1.3 Hz, 1H), 7.33 (t, *J* = 8.0 Hz, 1H), 7.07 – 7.03 (m, 2H), 6.91 (ddd, *J* = 8.2, 2.3, 1.0 Hz, 1H), 4.99 (s, 1H), 3.75 (s, 3H), 2.72 (s, 3H), 0.88 (s, 9H). $^{13}$C NMR (126 MHz, DMSO-$d_6$) δ 173.8, 160.3, 159.2, 146.2, 145.2, 141.7, 138.4, 134.7, 134.1, 132.7, 132.5, 130.9, 130.8, 129.5, 129.4, 129.3, 128.9, 126.0, 124.1, 119.8, 113.6, 113.3, 75.9, 70.5, 55.7, 28.3, 25.0.

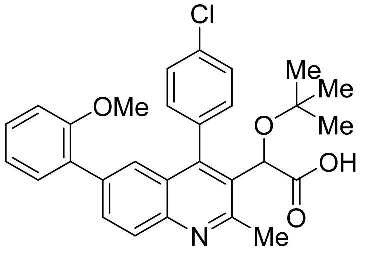

**2-(*Tert*-butoxy)-2-(4-(4-chlorophenyl)-6-(2-methoxyphenyl)-2-methylquinolin-3-yl)acetic acid (8i):** To a medium vial, ethyl 2-(*tert*-butoxy)-2-(4-(4-chlorophenyl)-6-(2-methoxyphenyl)-2-methylquinolin-3-yl)acetate (87 mg, 0.167 mmol), NaOH (27 mg, 0.669 mmol) was dissolved in ethanol (2 mL) and $H_2O$ (1 mL). The vial was purged with argon, capped, and refluxed at 80 °C for 16 h. After the reaction was completed, the solution was condensed *in vacuo*. The solid was washed with methylene chloride on filter paper. The solid then was dissolved in $H_2O$ and acidified with 1M HCl to pH 1. The aqueous solution was extracted with ethyl acetate (3x50 mL). The combined organic layers were dried with anhydrous sodium sulfate, and condensed *in vacuo* to give yellow solid. The solid was triturated with diethyl ether to give light yellow powder (36 mg, 44%).$^1$H NMR (500 MHz, DMSO-$d_6$) δ 8.26 (d, *J* = 8.6 Hz, 1H), 8.05 (d, *J* = 8.7 Hz, 1H), 7.77 – 7.67 (m, 2H), 7.52 – 7.42 (m, 3H), 7.34 (ddd, *J* = 8.3, 7.3, 1.7 Hz, 1H), 7.26 (dd, *J* = 7.6, 1.8 Hz, 1H), 7.08 (dd, *J* = 8.4, 1.1 Hz, 1H), 7.00 (td, *J* = 7.4, 1.1 Hz, 1H), 5.03 (s, 1H), 3.69 (s, 3H), 2.90 (s, 3H), 0.90 (s, 9H).$^{13}$C NMR (126 MHz, DMSO-$d_6$) δ 173.1, 158.3, 156.6, 134.8, 134.8, 132.4, 132.1, 131.6, 130.9, 130.4, 130.4, 130.4, 129.6, 129.0, 128.5, 128.5, 127.1, 126.3, 121.6, 112.5, 76.5, 70.2, 56.0, 28.2.

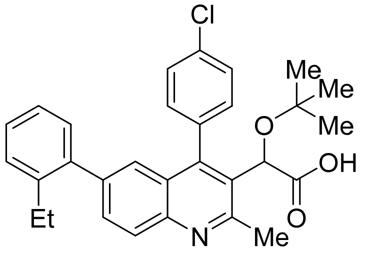

**2-(*Tert*-butoxy)-2-(4-(4-chlorophenyl)-6-(2-ethylphenyl)-2-methylquinolin-3-yl)acetic acid (8j):** To a medium vial, ethyl 2-(*tert*-butoxy)-2-(4-(4-chlorophenyl)-6-(2-ethylphenyl)-2-methylquinolin-3-yl)acetate (168 mg, 0.325 mmol), NaOH (52 mg, 1.30 mmol) was dissolved in ethanol (2 mL) and $H_2O$ (1 mL). The vial was purged with argon, capped, and refluxed at 80 °C for 16 h. After the reaction was completed, the solution was condensed *in vacuo*. The solid was re-dissolved in water and extracted with ethyl acetate (20 mL). The aqueous layers were acidified to pH 1 and extracted with ethyl acetate (30 mLx3). The combined organic layers were dried with anhydrous sodium sulfate, and condensed *in vacuo* to give a brown solid. The solid was triturated with diethyl ether to give white solid. (57 mg, 36%). $^1$H NMR (500 MHz, DMSO-$d_6$) δ 8.17 (s, 1H), 7.75 (d, *J* = 8.6 Hz, 1H), 7.63 (ddd, *J* = 30.2, 8.2, 2.3 Hz, 2H), 7.46 (dt, *J* = 12.5, 4.8 Hz, 2H), 7.25 (d, J = 3.7 Hz, 2H), 7.17 (ddd, *J* = 8.4, 5.1, 3.3 Hz, 1H), 7.13 – 7.06 (m, 2H), 4.99 (s, 1H), 2.82 (s, 3H), 2.40 (q, *J* = 7.5 Hz, 2H), 0.88 (d, *J* = 3.6 Hz, 12H). $^{13}$C NMR (126 MHz, DMSO-$d_6$) δ 173.3, 158.7, 148.6, 141.6, 140.6, 140.2, 134.6, 133.9, 132.9, 132.4, 132.0, 131.5, 130.3, 129.5, 129.0, 128.7, 126.5, 126.5, 126.1, 124.5, 76.3, 70.3, 28.2, 26.0, 16.3.

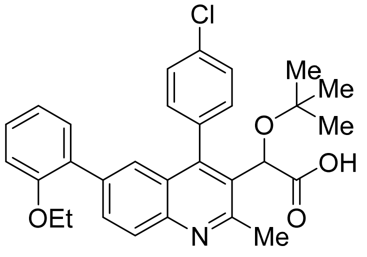

**2-(*Tert*-butoxy)-2-(4-(4-chlorophenyl)-6-(2-ethoxyphenyl)-2-methylquinolin-3-yl)acetic acid (8k):** To a medium vial, ethyl 2-(*tert*-butoxy)-2-(4-(4-chlorophenyl)-2-methyl-6-(2-ethoxy)quinolin-3-yl)acetate (158 mg, 0.297 mmol), NaOH (48 mg, 1.19 mmol) was dissolved in ethanol (2 mL) and $H_2O$ (1 mL). The vial was purged with argon, capped, and refluxed at 80 °C for 16 h. After the reaction was completed, the solution was condensed *in vacuo*. The solid was re-dissolved in water and extracted with ethyl acetate (20 mL). The aqueous layers were acidified to pH 1 and extracted with ethyl acetate (3x30 mL). The combined organic layers were dried with anhydrous sodium sulfate, and condensed *in vacuo* to give a brown solid. The solid was triturated with diethyl ether to give brown solid. (46 mg, 31%). $^1$H NMR (500 MHz, DMSO-$d_6$) δ 7.96 (d, *J* = 8.7 Hz, 1H), 7.82 (dd, *J* = 8.7, 2.0 Hz, 1H), 7.70 – 7.60 (m, 2H), 7.48 – 7.40 (m, 3H), 7.29 – 7.26 (m, 1H), 7.26 – 7.24 (m, 1H), 7.04 – 7.00 (m, 1H), 6.97 (td, *J* = 7.4, 1.0 Hz, 1H), 4.97 (s, 1H), 3.92 (qt, *J* = 6.9, 3.3 Hz, 2H), 2.71 (s, 3H), 1.08 (t, *J* = 7.0 Hz, 3H), 0.89 (s, 9H). $^{13}$C NMR (126 MHz, DMSO-$d_6$) δ 173.9, 158.9, 155.8, 145.6, 145.1, 136.3, 134.9, 134.0, 132.6, 132.3, 131.7, 131.1, 130.6, 129.8, 129.3, 129.3, 128.8, 128.4, 126.7, 125.6, 121.5, 113.3, 75.9, 70.5, 63.9, 28.3, 25.0, 14.9.

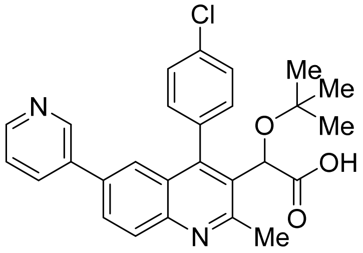

**2-(*Tert*-butoxy)-2-(4-(4-chlorophenyl)-2-methyl-6-(pyridin-3-yl)quinolin-3-yl)acetic acid (8m):** To a medium vial, ethyl 2-(*tert*-butoxy)-2-(4-(4-chlorophenyl)-2-methyl-6-(pyridin-3-yl)quinolin-3-yl)acetate (90 mg, 0.184 mmol), NaOH (29 mg, 0.736 mmol) was dissolved in ethanol (2 mL) and $H_2O$ (1 mL). The vial was purged with argon, capped, and refluxed at 80 °C for 16 h. After the reaction was completed, the solution was condensed *in vacuo*. The solid was washed with methylene chloride on filter paper. The solid then was dissolved in $H_2O$ and acidified with 1M HCl to pH 1. The aqueous solution was extracted with ethyl acetate (3x50 mL). The combined organic layers were dried with anhydrous sodium sulfate, and condensed *in vacuo* to give yellow solid. The solid was triturated with diethyl ether to give yellow solid (9 mg, 7%). $^1$H NMR (500 MHz, DMSO-$d_6$) δ 8.08 (d, *J* = 2.3 Hz, 2H), 8.04 (d, *J* = 8.0 Hz, 1H), 7.74 – 7.69 (m, 1H), 7.68 – 7.63 (m, 1H), 7.61 – 7.56 (m, 2H), 7.55 – 7.51 (m, 1H), 7.51 – 7.47 (m, 2H), 7.46 (d, *J* = 5.0 Hz, 1H), 5.00 (s, 1H), 2.74 (s, 3H), 0.88 (s, 9H). $^{13}$C NMR (126 MHz, DMSO-$d_6$) δ 173.8, 148.3, 146.3, 145.3, 135.5, 135.1, 134.4, 134.2, 132.7, 132.6, 132.5, 132.1, 132.0, 129.9, 129.5, 129.4, 129.2, 129.0, 126.1, 124.6, 124.5, 76.0, 70.4, 28.3, 25.1.

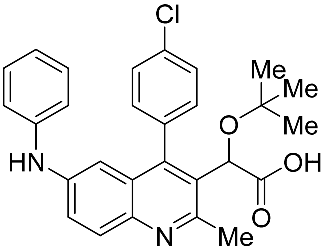

**2-(*Tert*-butoxy)-2-(4-(4-chlorophenyl)-2-methyl-6-(phenylamino)quinolin-3-yl)acetic acid (8n):** To a medium vial, ethyl 2-(*tert*-butoxy)-2-(4-(4-chlorophenyl)-2-methyl-6-(phenylamino)quinolin-3-yl)acetate (132 mg, 0.262 mmol), NaOH (42 mg, 1.05 mmol) was dissolved in ethanol (2 mL) and $H_2O$ (1 mL). The vial was purged with argon, capped, and refluxed at 80 °C for 16 h. After the reaction was completed, the solution was condensed *in vacuo*. The solid was re-dissolved in water and extracted with ethyl acetate (20 mL). The aqueous layers were acidified to pH 1 and extracted with ethyl acetate (3x30 mL). The combined organic layers were dried with anhydrous sodium sulfate, and condensed *in vacuo* to give a brown solid. The solid was triturated with diethyl ether to give orange solid (37 mg, 30%). $^1$H NMR (500 MHz, DMSO-$d_6$) δ 7.74 – 7.60 (m, 4H), 7.50 – 7.46 (m, 1H), 7.44 – 7.40 (m, 1H), 7.23 – 7.16 (m, 2H), 7.06 – 7.02 (m, 2H), 6.88 (t, *J* = 7.3 Hz, 1H), 6.79 (d, *J* = 2.5 Hz, 1H), 4.98 (s, 1H), 2.79 (s, 3H), 0.89 (s, 9H). $^{13}$C NMR (126 MHz, DMSO-$d_6$) δ 173.9, 154.90 143.9, 142.8, 142.1, 135.1, 133.9, 132.5, 130.4, 129.7, 129.3, 128.8, 127.3, 124.4, 123.1, 121.8, 121.3, 117.9, 117.6, 107.5, 75.9, 64.5, 28.2, 24.2.

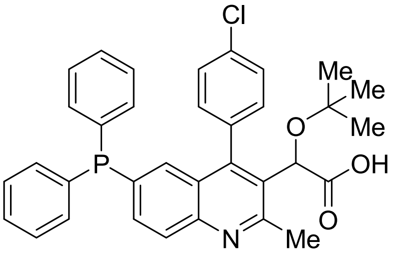

**2-(*Tert*-butoxy)-2-(4-(4-chlorophenyl)-6-(diphenylphosphaneyl)-2-methylquinolin-3-yl)acetic acid (8o):** To a medium vial, ethyl 2-(*tert*-butoxy)-2-(4-(4-chlorophenyl)-6-(diphenylphosphaneyl)-2-methylquinolin-3-yl)acetate (231 mg, 0.338 mmol), NaOH (62 mg, 1.55 mmol) was dissolved in ethanol (2 mL) and $H_2O$ (1 mL). The vial was purged with argon, capped, and refluxed at 80 °C for 16 h. After the reaction was completed, the solution was condensed *in vacuo*. The solid was re-dissolved in 0.1M NaOH solution (10 mL) and extracted with ethyl acetate (20 mL). The aqueous layers were acidified to pH 1 and extracted with ethyl acetate (3x30 mL). The combined organic layers were dried with anhydrous sodium sulfate, and condensed *in vacuo* to give a white solid (29 mg, 13%). $^1$H NMR (500 MHz, DMSO-$d_6$) δ 8.04 (dd, *J* = 8.7, 2.5 Hz, 1H), 7.82 – 7.73 (m, 1H), 7.70 – 7.60 (m, 1H), 7.59 – 7.33 (m, 13H), 7.32 – 7.17 (m, 1H), 4.94 (s, 1H), 2.73 (s, 3H), 0.84 (s, 9H). $^{13}$C NMR (126 MHz, DMSO-$d_6$) δ 172.6, 147.6, 134.1, 134.0, 133.1, 132.8, 132.6, 132.4, 132.3, 132.2, 132.1, 132.0, 132.0, 129.6, 129.5, 129.3, 129.3, 129.3, 129.2, 128.9, 126.5, 125.9, 125.3, 125.2, 75.8, 60.3, 28.3, 21.6.

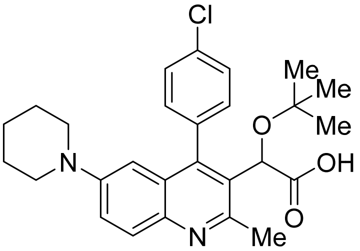

**2-(*Tert*-butoxy)-2-(4-(4-chlorophenyl)-2-methyl-6-(piperidin-1-yl)quinolin-3-yl)acetic acid (8p):** To a medium vial, ethyl 2-(*tert*-butoxy)-2-(4-(4-chlorophenyl)-2-methyl-6-(piperidin-1-yl)quinolin-3-yl)acetate (119 mg, 0.240 mmol), NaOH (39 mg, 0.962 mmol) was dissolved in ethanol (2 mL) and $H_2O$ (1 mL). The vial was purged with argon, capped, and refluxed at 80 °C for 16 h. After the reaction was completed, the solution was condensed *in vacuo*. The solid was re-dissolved in water and extracted with ethyl acetate (20 mL). The aqueous layers were acidified to pH 1 and extracted with ethyl acetate (3x30 mL). The combined organic layers were dried with anhydrous sodium

sulfate, and condensed *in vacuo* to give a brown solid. The solid was triturated with diethyl ether to give orange solid (71 mg, 63%). $^{1}$H NMR (500 MHz, DMSO-$d_6$) δ 7.99 (d, *J* = 9.2 Hz, 1H), 7.69 (ddd, *J* = 27.0, 8.8, 2.7 Hz, 2H), 7.64 – 7.57 (m, 1H), 7.42 (ddt, *J* = 7.0, 4.9, 2.2 Hz, 3H), 4.92 (s, 1H), 2.74 (s, 3H), 1.56 – 1.41 (m, 8H), 0.86 (d, *J* = 9.3 Hz, 11H). $^{13}$C NMR (126 MHz, DMSO-$d_6$) δ 173.9, 155.0, 150.0, 135.4, 133.8, 132.6, 132.2, 130.3, 129.4, 129.2, 128.8, 126.9, 123.3, 107.5, 75.8, 70.5, 50.0, 28.3, 25.6, 24.5, 24.2.

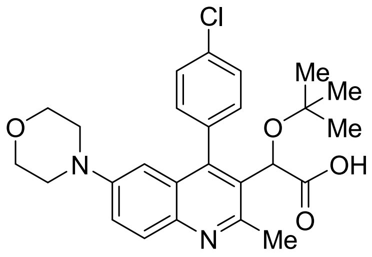


**2-(*Tert*-butoxy)-2-(4-(4-chlorophenyl)-2-methyl-6-morpholinoquinolin-3-yl)acetic acid (8q):** To a medium vial, ethyl 2-(*tert*-butoxy)-2-(4-(4-chlorophenyl)-2-methyl-6-morpholinoquinolin-3-yl)acetate (46 mg, 0.092 mmol), NaOH (15 mg, 0.367 mmol) was dissolved in ethanol (2 mL) and $H_2O$ (1 mL). The vial was purged with argon, capped, and refluxed at 80 °C for 16 h. After the reaction was completed, the solution was condensed *in vacuo*. The solid was re-dissolved in water and extracted with ethyl acetate (20 mL). The aqueous layers were acidified to pH 1 and extracted with ethyl acetate (3x30 mL). The combined organic layers were dried with anhydrous sodium sulfate, and condensed *in vacuo* to give a brown solid. The solid was triturated with diethyl ether to give orange solid (42 mg, 98%). $^{1}$H NMR (500 MHz, DMSO-$d_6$) δ 7.82 (d, *J* = 9.3 Hz, 1H), 7.69 – 7.55 (m, 3H), 7.40 (ddd, *J* = 26.3, 8.2, 2.2 Hz, 2H), 6.39 (d, *J* = 2.7 Hz, 1H), 4.90 (s, 1H), 3.66 – 3.63 (m, 4H), 3.01 – 2.89 (m, 4H), 2.64 (s, 3H), 0.85 (s, 9H). $^{13}$C NMR (126 MHz, DMSO-$d_6$) δ 173.8, 172.7, 159.1, 155.3, 149.5, 133.9, 132.6, 130.6, 128.9, 126.9, 122.4, 121.3, 118.9, 116.5, 114.1, 107.0, 75.8, 70.5, 66.4, 48.7, 28.2, 21.6.

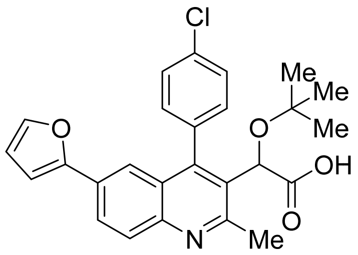


**2-(*Tert*-butoxy)-2-(4-(4-chlorophenyl)-6-(furan-2-yl)-2-methylquinolin-3-yl)acetic acid (8r):** To a medium vial, ethyl 2-(*tert*-butoxy)-2-(4-(4-chlorophenyl)-6-(furan-2-yl)-2-methylquinolin-3-yl)acetate (61 mg, 0.128 mmol), NaOH (21 mg, 0.512 mmol) was dissolved in ethanol (2 mL) and $H_2O$ (1 mL). The vial was purged with argon, capped, and refluxed at 80 °C for 16 h. After the reaction was completed, the solution was condensed *in vacuo*. The solid was washed with methylene chloride on filter paper. The solid then was dissolved in $H_2O$ and acidified with 1M HCl to pH 1. The aqueous solution was extracted with ethyl acetate (3x50 mL). The combined organic layers were dried with anhydrous sodium sulfate, and condensed *in vacuo* to give yellow solid. The solid was triturated with diethyl ether to give yellow powder (34 mg, 58%). $^{1}$H NMR (500 MHz, DMSO-$d_6$) δ 8.13 (dd, *J* = 8.8, 1.9 Hz, 1H), 8.04 (d, *J* = 8.8 Hz, 1H), 7.77 – 7.68 (m, 3H), 7.50 – 7.44 (m, 3H), 7.04 (dd, *J* = 3.4, 0.7 Hz, 1H), 6.57 (dd, *J* = 3.4, 1.8 Hz, 1H), 4.97 (s, 1H), 2.76 (s, 3H), 0.89 (s, 9H). $^{13}$C NMR (126 MHz, DMSO-$d_6$) δ 173.5, 158.6, 152.4, 144.5, 134.5, 134.2, 132.5, 132.2, 131.5, 129.5, 129.1, 129.1, 128.1, 127.4, 126.5, 119.5, 113.0, 108.5, 76.2, 70.3, 28.2, 24.01.

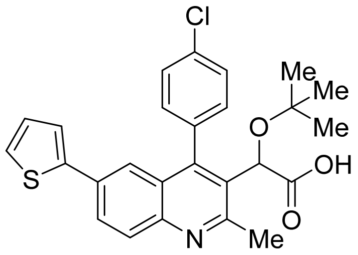


**2-(*Tert*-butoxy)-2-(4-(4-chlorophenyl)-2-methyl-6-(thiophen-2-yl)quinolin-3-yl)acetic acid (8s):** To a medium vial, ethyl 2-(*tert*-butoxy)-2-(4-(4-chlorophenyl)-2-methyl-6-(thiophen-2-yl)quinolin-3-yl)acetate (154 mg, 0.310 mmol), NaOH (50 mg, 1.24 mmol) was dissolved in ethanol (2 mL) and $H_2O$ (1 mL). The vial was purged with argon, capped, and refluxed at 80 °C for 16 h. After the reaction was completed, the solution was condensed *in vacuo*. The solid was re-dissolved in water and extracted with ethyl acetate (20 mL). The aqueous layers were acidified to pH 1 and extracted with ethyl acetate (3x30 mL). The combined organic layers were dried with anhydrous sodium sulfate, and condensed *in vacuo* to give a brown solid (74 mg, 51%). $^{1}$H NMR (500 MHz, DMSO-$d_6$) δ 8.11 – 7.96 (m, 2H), 7.71 (ddd, *J* = 24.8, 8.1, 2.3 Hz, 2H), 7.51 – 7.44 (m, 4H), 7.37 (d, *J* = 2.0 Hz, 1H), 7.08 (dd, *J* = 5.1, 3.6 Hz, 1H), 4.98 (s, 1H), 2.76 (s, 3H), 0.88 (s, 9H). $^{13}$C NMR (126 MHz, DMSO-$D_6$) δ 173.7, 159.0, 142.9, 134.4, 134.3, 132.7, 132.3, 132.1, 132.0, 131.2, 129.5, 129.3, 128.9, 128.3, 127.3, 126.4, 125.4, 122.1, 76.0, 70.4, 40.5, 40.4, 40.2, 40.0, 39.9, 39.7, 39.5, 28.3, 24.7.

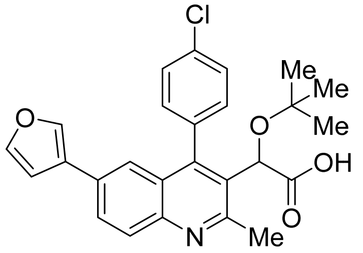

**2-(*Tert*-butoxy)-2-(4-(4-chlorophenyl)-6-(furan-3-yl)-2-methylquinolin-3-yl)acetic acid (8t):** To a medium vial, ethyl 2-(*tert*-butoxy)-2-(4-(4-chlorophenyl)-6-(furan-3-yl)-2-methylquinolin-3-yl)acetate (158 mg, 0.350 mmol), NaOH (53 mg, 1.32 mmol) was dissolved in ethanol (2 mL) and $H_2O$ (1 mL). The vial was purged with argon, capped, and refluxed at 80 °C for 16 h. After the reaction was completed, the solution was condensed *in vacuo*. The solid was re-dissolved in water and extracted with ethyl acetate (20 mL). The aqueous layers were acidified to pH 1 and extracted with ethyl acetate (3x30 mL). The combined organic layers were dried with anhydrous sodium sulfate, and condensed *in vacuo* to give a brown solid (25 mg, 17%). $^1$H NMR (500 MHz, DMSO-$d_6$) δ 8.15 (t, *J* = 1.3 Hz, 1H), 7.99 (d, *J* = 1.9 Hz, 1H), 7.75 – 7.63 (m, 3H), 7.45 (ddd, *J* = 20.9, 8.2, 2.2 Hz, 2H), 7.31 (t, *J* = 1.3 Hz, 1H), 6.72 – 6.65 (m, 1H), 4.97 (s, 1H), 2.72 (s, 3H), 0.87 (s, 9H).$^{13}$C NMR (126 MHz, DMSO-$d_6$) δ 173.8, 172.6, 167.6, 158.5, 145.3, 144.8, 140.7, 134.2, 132.6, 132.3, 129.4, 129.0, 125.8, 124.4, 123.9, 122.0, 109.1, 76.0, 70.4, 28.3, 21.6.

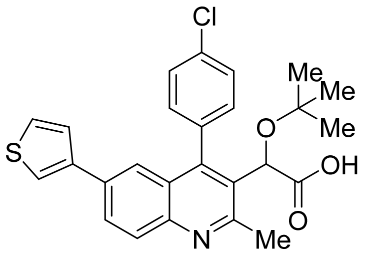

**2-(*Tert*-butoxy)-2-(4-(4-chlorophenyl)-2-methyl-6-(thiophen-3-yl)quinolin-3-yl)acetic acid (8u):** To a medium vial, ethyl 2-(*tert*-butoxy)-2-(4-(4-chlorophenyl)-2-methyl-6-(thiophen-3-yl)quinolin-3-yl)acetate (69 mg, 0.140 mmol), NaOH (22 mg, 0.560 mmol) was dissolved in ethanol (2 mL) and $H_2O$ (1 mL). The vial was purged with argon, capped, and refluxed at 80 °C for 16 h. After the reaction was completed, the solution was condensed *in vacuo*. The solid was washed with methylene chloride on filter paper. The solid then was dissolved in $H_2O$ and acidified with 1M HCl to pH 1. The aqueous solution was extracted with ethyl acetate (3x50 mL). The combined organic layers were dried with anhydrous sodium sulfate, and condensed *in vacuo* to give yellow solid. The solid was triturated with diethyl ether to give yellow powder (31 mg, 48%). $^1$H NMR (500 MHz, DMSO-$d_6$) δ 8.06 (dd, *J* = 8.7, 2.0 Hz, 1H), 7.97 (d, *J* = 8.7 Hz, 1H), 7.79 (dd, *J* = 3.1, 1.4 Hz, 1H), 7.69 (ddd, *J* = 28.6, 8.2, 2.3 Hz, 2H), 7.60 (dd, *J* = 5.0, 2.9 Hz, 1H), 7.47 (ddd, *J* = 15.9, 8.2, 2.2 Hz, 2H), 7.41 (d, *J* = 1.9 Hz, 1H), 7.30 (dd, *J* = 5.1, 1.4 Hz, 1H), 4.97 (s, 1H), 2.70 (s, 3H), 0.88 (s, 9H). $^{13}$C NMR (126 MHz, DMSO-$d_6$) δ 173.9, 158.9, 146.0, 145.0, 141.3, 134.6, 134.1, 133.5, 132.7, 132.5, 130.8, 129.6, 129.4, 129.0, 128.3, 126.6, 126.2, 122.7, 75.9, 70.4, 28.3, 25.0.

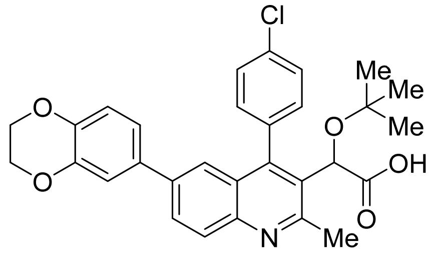

**2-(*Tert*-butoxy)-2-(4-(4-chlorophenyl)-6-(2,3-dihydrobenzo[*b*][1,4]dioxin-6-yl)-2-methylquinolin-3-yl)acetic acid:** To a medium vial, ethyl 2-(*tert*-butoxy)-2-(4-(4-chlorophenyl)-6-(2,3-dihydrobenzo[*b*]-[1,4]dioxin-6-yl)-2-methylquinolin-3-yl)acetate (121 mg, 0.222 mmol), NaOH (36 mg, 0.886 mmol) was dissolved in ethanol (2 mL) and $H_2O$ (1 mL). The vial was purged with argon, capped, and refluxed at 80 °C for 16 h. After the reaction was completed, the solution was condensed *in vacuo*. The solid was re-dissolved in water and extracted with ethyl acetate (20 mL). The aqueous layers were acidified to pH 1 and extracted with ethyl acetate (3x30 mL). The combined organic layers were dried with anhydrous sodium sulfate, and condensed *in vacuo* to give a brown solid. The solid was further triturated with diethyl ether to give bright yellow powder (47 mg, 41%). $^1$H NMR (500 MHz, DMSO-$d_6$) δ 8.00 – 7.91 (m, 2H), 7.73 – 7.65 (m, 2H), 7.50 – 7.44 (m, 2H), 7.29 (dd, *J* = 2.0, 0.6 Hz, 1H), 6.97 (dd, *J* = 6.7, 2.2 Hz, 2H), 6.89 (d, *J* = 9.0 Hz, 1H), 4.98 (s, 1H), 4.21 (s, 4H), 2.72 (s, 3H), 0.88 (s, 9H). $^{13}$C NMR (126 MHz, DMSO-$d_6$) δ 173.8, 158.8, 145.4, 144.3, 144.0, 138.2, 134.6, 134.2, 133.3, 132.7, 132.4, 130.9, 129.4, 129.3, 128.9, 126.1, 123.2, 120.3, 118.3, 115.9, 76.0, 70.4, 64.7, 28.3.

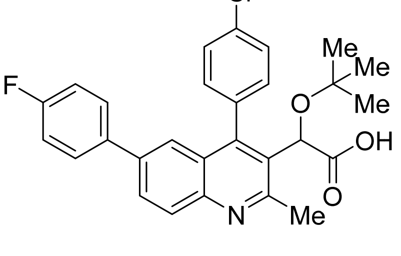

**2-(*Tert*-butoxy)-2-(4-(4-chlorophenyl)-6-(4-fluorophenyl)-2-methylquinolin-3-yl)acetic acid:** To a medium vial, ethyl 2-(*tert*-butoxy)-2-(4-(4-chlorophenyl)-6-(4-fluorophenyl)-2-methylquinolin-3-yl)acetate (159 mg, 0.314 mmol), NaOH (50 mg, 1.26 mmol) was dissolved in ethanol (2 mL) and $H_2O$ (1 mL). The vial was purged with argon, capped, and refluxed at 80 °C for 16 h. After the reaction was completed, the solution was condensed *in vacuo*. The solid was re-dissolved in water and extracted with ethyl acetate (20 mL). The aqueous layers were acidified to pH 1 and extracted with ethyl acetate (30 mLx3). The combined organic layers

were dried with anhydrous sodium sulfate, and condensed *in vacuo* to give a brown solid. The solid was triturated with diethyl ether to give orange solid (40 mg, 27%). $^1$H NMR (500 MHz, DMSO-*d*$_6$) δ 8.05 (d, *J* = 8.7 Hz, 1H), 8.01 (dd, *J* = 8.7, 2.0 Hz, 1H), 7.74 – 7.64 (m, 2H), 7.58 – 7.54 (m, 2H), 7.49 (ddt, *J* = 6.4, 4.3, 2.0 Hz, 2H), 7.37 (d, *J* = 2.0 Hz, 1H), 7.28 – 7.23 (m, 2H), 4.98 (s, 1H), 2.74 (s, 3H), 0.88 (s, 9H). $^{13}$C NMR (126 MHz, DMSO-*d*$_6$) δ 173.7, 163.6, 161.7, 159.1, 137.8, 136.6, 134.4, 134.2, 132.7, 132.4, 131.1, 129.6, 129.5, 129.0, 126.1, 123.9, 116.7, 116.6, 116.6, 116.5, 116.2, 116.1, 76.0, 70.4, 28.3, 24.7. $^{19}$F NMR (471 MHz, DMSO-*d*$_6$) δ -114.6.

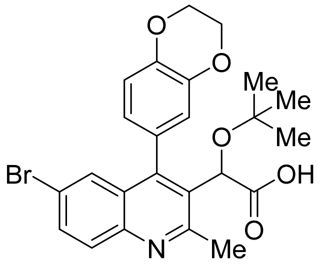


**2-(6-Bromo-4-(2,3-dihydrobenzo[*b*][1,4]dioxin-6-yl)-2-methylquinolin-3-yl)-2-(tert-butoxy)acetic acid (17):** To a medium vial, ethyl 2-(6-bromo-4-(2,3-dihydrobenzo[*b*][1,4]dioxin-6-yl)-2-methylquinolin-3-yl)-2-(*tert*-butoxy)acetate (77 mg, 0.149 mmol), NaOH (24 mg, 0.597 mmol) was dissolved in ethanol (2 mL) and $H_2O$ (1 mL). The vial was purged with argon, capped, and refluxed at 80 °C for 16 h. After the reaction was completed, the solution was condensed *in vacuo*. The solid was re-dissolved in water and extracted with ethyl acetate (20 mL). The aqueous layers were acidified to pH 1 and extracted with ethyl acetate (3x30 mL). The combined organic layers were dried with anhydrous sodium sulfate, and condensed *in vacuo* to give a yellow solid (69 mg, 96%). $^1$H NMR (500 MHz, DMSO-*d*$_6$) δ 7.91 (d, *J* = 8.9 Hz, 1H), 7.84 (ddd, *J* = 7.9, 2.3, 1.1 Hz, 1H), 7.50 (dd, *J* = 14.4, 2.2 Hz, 1H), 7.13 (dd, *J* = 10.1, 8.2 Hz, 1H), 7.01 – 6.83 (m, 2H), 5.14 (d, *J* = 0.7 Hz, 1H), 4.43 – 4.18 (m, 4H), 2.72 (d, *J* = 1.2 Hz, 3H), 0.93 (d, *J* = 6.2 Hz, 9H). $^{13}$C NMR (126 MHz, DMSO-*d*$_6$) (isomer) δ 173.9, 159.9 (159.9), 145.3 (145.30), 145.0 (144.9), 144.4 (144.3), 143.9 (143.7), 132.9, 131.5 (131.5), 131.2, 128.6 (128.5), 127.9 (127.8), 123.7 (123.4), 119.7 (119.5), 119.0 (119.0), 118.0, 117.6, 76.0, 75.9, 70.4, 64.7, 28.3, 25.0.

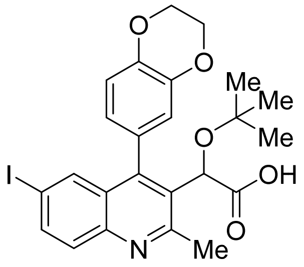


**2-(*Tert*-butoxy)-2-(4-(2,3-dihydrobenzo[*b*][1,4]dioxin-6-yl)-6-iodo-2-methylquinolin-3-yl)acetic acid:** To a medium vial, ethyl 2-(*tert*-butoxy)-2-(4-(2,3-dihydrobenzo[*b*][1,4]dioxin-6-yl)-6-iodo-2-methylquinolin-3-yl)acetate (21 mg, 0.037 mmol), NaOH (6 mg, 0.148 mmol) was dissolved in ethanol (2 mL) and $H_2O$ (1 mL). The vial was purged with argon, capped, and refluxed at 80 °C for 16 h. After the reaction was completed, the solution was condensed *in vacuo*. The solid was re-dissolved in water and extracted with ethyl acetate (20 mL). The aqueous layer was acidified to pH 1 and extracted with ethyl acetate (3x30 mL). The combined organic layers were dried with anhydrous sodium sulfate, and condensed *in vacuo* to give a yellow solid (9.5 mg, 48%).$^1$H NMR (500 MHz, DMSO-*d*$_6$) δ 7.96 – 7.87 (m, 1H), 7.73 – 7.31 (m, 2H), 7.10 – 7.02 (m, 1H), 6.95 – 6.75 (m, 2H), 5.19 – 5.00 (m, 1H), 4.46 – 4.15 (m, 4H), 2.73 – 2.52 (m, 3H), 0.95 – 0.81 (m, 9H). $^{13}$C NMR (126 MHz, DMSO-*d*$_6$) (isomer) δ 173.9, 159.8, 145.6, 144.7 (144.6), 144.3, 143.9 (143.6), 138.2, 135.0 (134.9), 131.3 (130.9), 129.8, 128.8, 128.3 (127.9), 123.7 (123.4), 119.5, 118.0 (117.6), 92.9, 76.0 (75.8), 70.5 (70.3), 64.8 (64.7), 28.3, 25.0 (25.0).

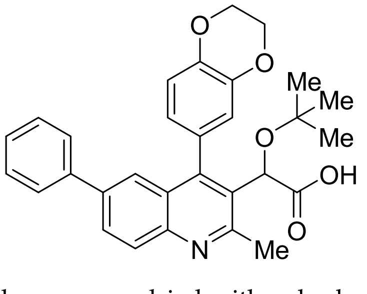


**2-(*Tert*-butoxy)-2-(4-(2,3-dihydrobenzo[*b*][1,4]dioxin-6-yl)-2-methyl-6-phenylquinolin-3-yl)acetic acid:** To a medium vial, ethyl 2-(*tert*-butoxy)-2-(4-(2,3-dihydrobenzo[*b*][1,4]dioxin-6-yl)-2-methyl-6-phenylquinolin-3-yl)acetate (88 mg, 0.172 mmol), NaOH (28 mg, 0.688 mmol) was dissolved in ethanol (2 mL) and $H_2O$ (1 mL). The vial was purged with argon, capped, and refluxed at 80 °C for 16 h. After the reaction was completed, the solution was condensed *in vacuo*. The solid was re-dissolved in water and extracted with ethyl acetate (20 mL). The aqueous layers were acidified to pH 1 and extracted with ethyl acetate (3x30 mL). The combined organic layers were dried with anhydrous sodium sulfate, and condensed *in vacuo* to give a yellow solid (45 mg, 54%). $^1$H NMR (500 MHz, DMSO-*d*$_6$) δ 8.00 (d, *J* = 8.6 Hz, 1H), 7.98 – 7.93 (m, 1H), 7.56 – 7.49 (m, 3H), 7.45 – 7.39 (m, 2H), 7.36 – 7.30 (m, 1H), 7.08 (dd, *J* = 14.8, 8.2 Hz, 1H), 7.01 – 6.86 (m, 2H), 5.13 (d, *J* = 5.6 Hz, 1H), 4.42 – 4.19 (m, 4H), 2.69 (d, *J* = 2.6 Hz, 3H), 0.89 (d, *J* = 9.7 Hz, 9H). $^{13}$C NMR (126 MHz, DMSO-*d*$_6$) (isomer) δ 173.5 (173.4), 172.0, 158.6 (158.5), 145.5 (145.5), 145.3 (145.2), 143.5 (143.5), 143.2 (142.9), 139.8 (139.7), 137.8 (137.7), 130.4 (130.4), 129.1 (128.9), 128.5 (128.4), 127.9 (127.9), 127.6, 126.9 (126.9), 125.9 (125.8), 123.7 (123.6), 123.1 (123.0), 121.5, 119.0 (118.5), 117.3 (116.8), 113.8, 75.2 (75.2), 69.9 (69.8), 64.2 (64.0), 55.1, 27.7 (27.7), 24.4 (24.4).

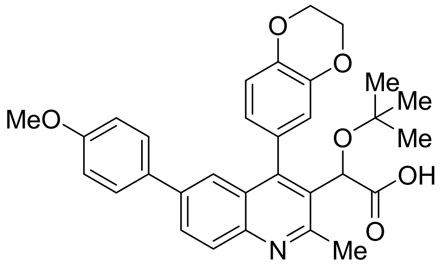


**2-(*Tert*-butoxy)-2-(4-(2,3-dihydrobenzo[*b*][1,4]dioxin-6-yl)-6-(4-methoxyphenyl)-2-methylquinolin-3-yl)acetic acid:** To a medium vial, ethyl 2-(*tert*-butoxy)-2-(4-(2,3-dihydrobenzo[*b*][1,4]dioxin-6-yl)-6-(4-methoxyphenyl)-2-methylquinolin-3-yl)acetate (129 mg, 0.238 mmol), NaOH (38 mg, 0.953 mmol) was dissolved in ethanol (2 mL) and $H_2O$ (1 mL). The vial was purged with argon, capped, and refluxed at 80 °C for 16 h. After the reaction was completed, the solution was condensed *in vacuo*. The solid was re-dissolved in water and extracted with ethyl acetate (20 mL). The aqueous layers were acidified to pH 1 and extracted with ethyl acetate (3x30 mL). The combined organic layers were dried with anhydrous sodium sulfate, and condensed *in vacuo* to give a yellow solid (107 mg, 88%). $^1$H NMR (500 MHz, DMSO-$d_6$) δ 7.96 (d, $J$ = 8.7 Hz, 1H), 7.92 (ddd, $J$ = 8.7, 3.8, 2.0 Hz, 1H), 7.48 (dd, $J$ = 4.3, 2.0 Hz, 1H), 7.47 – 7.40 (m, 2H), 7.07 (dd, $J$ = 18.0, 8.2 Hz, 1H), 7.02 – 6.92 (m, 3H), 6.89 – 6.84 (m, 1H), 5.14 (d, $J$ = 4.8 Hz, 1H), 4.36 – 4.20 (m, 4H), 3.72 (s, 3H), 2.70 (d, $J$ = 3.3 Hz, 3H), 0.89 (d, $J$ = 10.9 Hz, 9H). $^{13}$C NMR (126 MHz, DMSO-$d_6$) (isomer) δ 174.1 (174.1), 172.6, 159.7, 158.8 (158.7), 152.6 (151.7), 146.0 (145.6), 144.1 (144.1), 143.9 (143.6), 138.2 (138.1), 132.6 (132.6), 131.0 (131.0), 129.2 (128.9), 128.6 (128.6), 128.5, 126.6 (126.5), 123.8 (123.6), 123.4 (123.3), 119.6 (119.1), 117.9 (117.5), 116.2, 115.1, 75.9 (75.8), 70.5 (70.5), 64.8 (64.7), 60.3, 55.7, 28.3 (28.3), 24.9 (24.8).

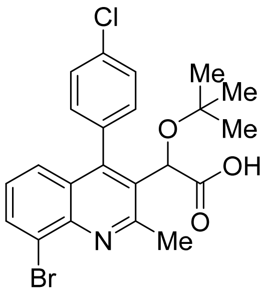


**2-(8-Bromo-4-(4-chlorophenyl)-2-methylquinolin-3-yl)-2-(*tert*-butoxy)acetic acid (16aa):** To a medium vial, ethyl 2-(8-bromo-4-(4-chlorophenyl)-2-methylquinolin-3-yl)-2-(*tert*-butoxy)acetate (108 mg, 0.217 mmol), NaOH (35 mg, 0.870 mmol) was dissolved in ethanol (2 mL) and $H_2O$ (1 mL). The vial was purged with argon, capped, and refluxed at 80 °C for 16 h. After the reaction was completed, the solution was condensed *in vacuo*. The solid was re-dissolved in water and extracted with ethyl acetate (20 mL). The aqueous layers were acidified to pH 1 and extracted with ethyl acetate (3x30 mL). The combined organic layers were dried with anhydrous sodium sulfate, and condensed *in vacuo* to give a brown solid (98 mg, 98%). $^1$H NMR (500 MHz, DMSO-$d_6$) δ 8.12 (dd, $J$ = 7.4, 1.3 Hz, 1H), 7.75 – 7.68 (m, 2H), 7.46 (ddd, $J$ = 32.7, 8.2, 2.2 Hz, 2H), 7.40 – 7.35 (m, 1H), 7.27 (dd, $J$ = 8.4, 1.3 Hz, 1H), 5.02 (s, 1H), 2.82 (s, 3H), 0.93 (s, 9H). $^{13}$C NMR (126 MHz, DMSO-$d_6$) δ 173.6, 160.2, 145.6, 143.2, 134.5, 134.3, 133.6, 132.5, 132.3, 131.4, 129.4, 128.9, 127.5, 127.5, 126.8, 124.3, 76.1, 70.4, 28.3, 25.4.

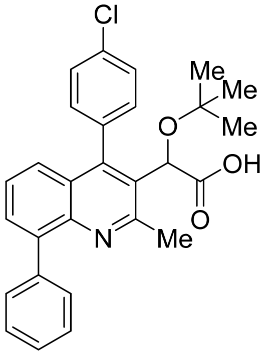


**2-(*Tert*-butoxy)-2-(4-(4-chlorophenyl)-2-methyl-8-phenylquinolin-3-yl)acetic acid (16ab):** To a medium vial, ethyl 2-(*tert*-butoxy)-2-(4-(4-chlorophenyl)-2-methyl-8-phenylquinolin-3-yl)acetate (95 mg, 0.195 mmol), NaOH (31 mg, 0.780 mmol) was dissolved in ethanol (2 mL) and $H_2O$ (1 mL). The vial was purged with argon, capped, and refluxed at 80 °C for 16 h. After the reaction was completed, the solution was condensed *in vacuo*. The solid was re-dissolved in water and extracted with ethyl acetate (20 mL). The aqueous layers were acidified to pH 1 and extracted with ethyl acetate (3x30 mL). The combined organic layers were dried with anhydrous sodium sulfate, and condensed *in vacuo* to give a brown solid (23 mg, 26%). $^1$H NMR (500 MHz, DMSO-$d_6$) δ 7.77 – 7.66 (m, 5H), 7.53 (dd, $J$ = 8.4, 7.1 Hz, 1H), 7.51 – 7.44 (m, 4H), 7.43 – 7.37 (m, 1H), 7.25 (dd, $J$ = 8.5, 1.4 Hz, 1H), 5.00 (s, 1H), 2.66 (s, 3H), 0.92 (s, 9H). $^{13}$C NMR (126 MHz, DMSO-$d_6$) δ 173.8, 145.3, 143.9, 139.9, 139.8, 135.1, 134.0, 132.6, 132.4, 131.3, 130.7, 129.3, 128.9, 128.2, 127.6, 126.7, 126.4, 126.3, 75.9, 70.5, 28.3, 25.4.

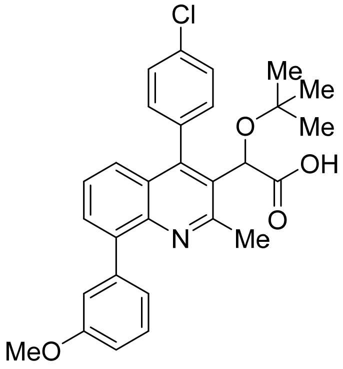

**2-(*Tert*-butoxy)-2-(4-(4-chlorophenyl)-8-(3-methoxyphenyl)-2-methylquinolin-3-yl)acetic acid (16ac):** To a medium vial, ethyl 2-(*tert*-butoxy)-2-(4-(4-chlorophenyl)-8-(3-methoxyphenyl)-2-methylquinolin-3-yl)acetate (101 mg, 0.194 mmol), NaOH (31 mg, 0.776 mmol) was dissolved in ethanol (2 mL) and $H_2O$ (1 mL). The vial was purged with argon, capped, and refluxed at 80 °C for 16 h. After the reaction was completed, the solution was condensed *in vacuo*. The solid was re-dissolved in water and extracted with ethyl acetate (20 mL). The aqueous layers were acidified to pH 1 and extracted with ethyl acetate (3x30 mL). The combined organic layers were dried with anhydrous sodium sulfate, and condensed *in vacuo* to give a brown solid (94 mg, 99%). $^1$H NMR (500 MHz, DMSO-$d_6$) δ 7.69 (ddd, *J* = 7.1, 5.0, 1.9 Hz, 2H), 7.64 (dd, *J* = 8.1, 2.3 Hz, 1H), 7.51 – 7.42 (m, 2H), 7.40 (dd, *J* = 8.2, 2.2 Hz, 1H), 7.33 (t, *J* = 7.9 Hz, 1H), 7.26 (dd, *J* = 2.6, 1.5 Hz, 1H), 7.23 – 7.14 (m, 2H), 6.93 (ddd, *J* = 8.4, 2.6, 1.0 Hz, 1H), 4.97 (s, 1H), 3.77 (s, 3H), 2.65 (s, 3H), 0.88 (s, 9H). $^{13}$C NMR (126 MHz, DMSO-$d_6$) δ 173.8, 159.1, 158.4, 145.3, 143.9, 141.2, 139.6, 135.1, 134.1, 132.6, 132.3, 130.6, 130.2, 129.3, 129.1, 128.9, 126.6, 126.4, 126.4, 123.5, 117.0, 113.2, 75.9, 70.5, 55.6, 28.3, 25.4.

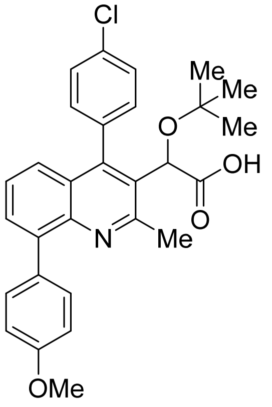

**2-(*Tert*-butoxy)-2-(4-(4-chlorophenyl)-8-(4-methoxyphenyl)-2-methylquinolin-3-yl)acetic acid (16ad):** To a medium vial, ethyl 2-(*tert*-butoxy)-2-(4-(4-chlorophenyl)-8-(4-methoxyphenyl)-2-methylquinolin-3-yl)acetate (41 mg, 0.079 mmol), NaOH (13 mg, 0.317 mmol) was dissolved in ethanol (2 mL) and $H_2O$ (1 mL). The vial was purged with argon, capped, and refluxed at 80 °C for 16 h. After the reaction was completed, the solution was condensed *in vacuo*. The solid was re-dissolved in water and extracted with ethyl acetate (20 mL). The aqueous layers were acidified to pH 1 and extracted with ethyl acetate (3x30 mL). The combined organic layers were dried with anhydrous sodium sulfate, and condensed *in vacuo* to give a faint yellow solid (40 mg, 99%). $^1$H NMR (500 MHz, DMSO-$d_6$) δ 7.69 (dd, *J* = 8.2, 2.3 Hz, 1H), 7.64 (dt, *J* = 8.0, 1.8 Hz, 2H), 7.62 – 7.59 (m, 2H), 7.48 – 7.42 (m, 2H), 7.39 (dd, *J* = 8.2, 2.2 Hz, 1H), 7.16 (dd, *J* = 8.4, 1.4 Hz, 1H), 7.02 – 6.98 (m, 2H), 4.96 (s, 1H), 3.79 (s, 3H), 0.88 (s, 9H). $^{13}$C NMR (126 MHz, DMSO-$d_6$) δ 173.8, 159.1, 158.2, 145.3, 144.0, 139.4, 135.2, 134.0, 132.6, 132.4, 132.3, 132.0, 130.2, 130.1, 129.3, 128.9, 126.7, 126.5, 125.8, 113.7, 75.9, 60.3, 55.6, 28.3, 25.4.

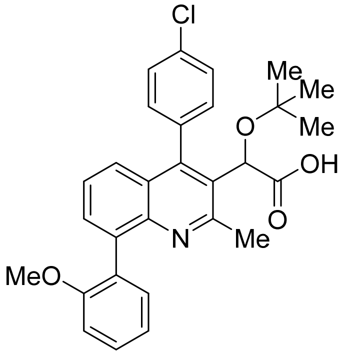

**2-(*Tert*-butoxy)-2-(4-(4-chlorophenyl)-8-(2-methoxyphenyl)-2-methylquinolin-3-yl)acetic acid (16ae):** To a medium vial, ethyl 2-(*tert*-butoxy)-2-(4-(4-chlorophenyl)-8-(2-methoxyphenyl)-2-methylquinolin-3-yl)acetate (28 mg, 0.054 mmol), NaOH (8 mg, 0.201 mmol) was dissolved in ethanol (2 mL) and $H_2O$ (1 mL). The vial was purged with argon, capped, and refluxed at 80 °C for 16 h. After the reaction was completed, the solution was condensed *in vacuo*. The solid was re-dissolved in water and extracted with ethyl acetate (20 mL). The aqueous layers were acidified to pH 1 and extracted with ethyl acetate (3x30 mL). The combined organic layers were dried with anhydrous sodium sulfate, and condensed *in vacuo* to give a brown solid (28 mg, 99%). $^1$H NMR (500 MHz, DMSO-$d_6$) δ 7.68 (ddd, *J* = 24.4, 8.2, 2.4 Hz, 2H), 7.55 (dd, *J* = 7.1, 1.5 Hz, 1H), 7.48 – 7.41 (m, 3H), 7.38 – 7.33 (m, 1H), 7.23 – 7.19 (m, 2H), 7.11 (dd, *J* = 8.3, 1.1 Hz, 1H), 7.00 (td, *J* = 7.4, 1.1 Hz, 1H), 4.96 (s, 1H), 3.63 (s, 3H), 2.55 (s, 3H), 0.88 (s, 9H). $^{13}$C NMR (126 MHz, DMSO-$d_6$) δ 173.9, 158.1, 157.7, 135.2, 135.1, 134.0, 132.6, 132.4, 132.4, 130.0, 129.3, 129.3, 128.9, 126.4, 126.4, 126.1, 126.1, 126.1, 120.5, 112.0, 75.9, 70.5, 55.9, 28.3, 25.3.

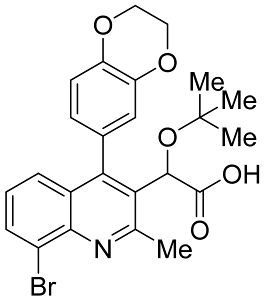

**2-(8-Bromo-4-(2,3-dihydrobenzo[*b*][1,4]dioxin-6-yl)-2-methylquinolin-3-yl)-2-(*tert*-butoxy)acetic acid (16ba):** To a medium vial, ethyl 2-(8-bromo-4-(2,3-dihydrobenzo[*b*][1,4]dioxin-6-yl)-2-methylquinolin-3-yl)-2-(*tert*-butoxy)acetate (100 mg, 0.194 mmol), NaOH (31 mg, 0.778 mmol) was dissolved in ethanol (2 mL) and $H_2O$ (1 mL). The vial was purged with argon, capped, and refluxed at 80 °C for 16 h. After the reaction was completed, the solution was condensed *in vacuo*. The solid was re-dissolved in water and extracted with ethyl acetate (20 mL). The aqueous layers were acidified to pH 1 and extracted with ethyl acetate (3x30 mL). The combined organic layers were dried with anhydrous sodium sulfate, and condensed *in vacuo* to give a faint yellow solid (80 mg, 85%).$^1$H NMR (500 MHz, DMSO-$d_6$) δ 8.09 – 7.87 (m, 1H), 7.70 – 7.39 (m, 1H), 7.39 – 7.30 (m, 2H), 7.06 (ddd, *J* = 14.2, 8.2, 2.4 Hz, 1H), 6.95 – 6.85 (m, 1H), 6.82 – 6.76 (m, 1H), 5.11 (dd, *J* = 8.7, 5.3 Hz, 1H), 4.39 – 4.23 (m, 4H), 2.70 (d, *J* = 24.7 Hz, 3H), 0.89 (dd, *J* = 3.3, 1.8 Hz, 9H). $^{13}$C NMR (126 MHz, DMSO-$d_6$) (isomer) δ 173.9 (173.9), 172.6, 160.2 (160.2), 146.4 (146.4), 144.3 (144.2), 143.8 (143.6), 143.2, 133.3 (131.5), 128.3, 127.9 (127.9), 127.2, 127.1 (127.0), 124.2 (124.2), 123.6 (123.5), 119.5 (119.2), 117.9 (117.5), 76.0 (75.9), 70.4 (70.4), 64.7 (64.7), 60.3, 28.3, 25.3.

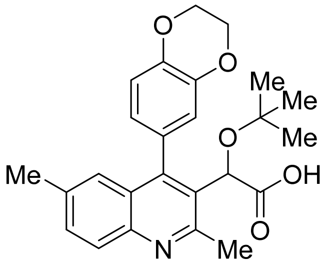

**2-(*Tert*-butoxy)-2-(4-(2,3-dihydrobenzo[*b*][1,4]dioxin-6-yl)-2,6-dimethylquinolin-3-yl)acetic acid (18):** To a medium vial, ethyl 2-(*tert*-butoxy)-2-(4-(2,3-dihydrobenzo[*b*][1,4]dioxin-6-yl)-2,6-dimethylquinolin-3-yl)acetate (68 mg, 0.151 mmol), NaOH (36 mg, 0.906 mmol) was dissolved in ethanol (2 mL) and $H_2O$ (1 mL). The vial was purged with argon, capped, and refluxed at 80 °C for 16 h. After the reaction was completed, the solution was condensed *in vacuo*. The solid was re-dissolved in water and extracted with ethyl acetate (20 mL). The aqueous layers were acidified to pH 1 and extracted with ethyl acetate (3x30 mL). The combined organic layers were dried with anhydrous sodium sulfate, and condensed *in vacuo* to give a faint yellow solid (43 mg, 68%). $^1$H NMR (500 MHz, DMSO-$d_6$) δ 7.99 (dd, *J* = 8.5, 4.5 Hz, 1H), 7.73 – 7.57 (m, 1H), 7.21 (d, *J* = 15.6 Hz, 1H), 7.08 (dd, *J* = 12.2, 8.2 Hz, 1H), 6.97 – 6.85 (m, 1H), 6.85 – 6.76 (m, 1H), 5.11 (s, 1H), 4.38 – 4.23 (m, 4H), 2.77 (d, *J* = 4.3 Hz, 3H), 2.36 (d, *J* = 7.2 Hz, 3H), 0.89 (d, *J* = 6.7 Hz, 9H). $^{13}$C NMR (126 MHz, DMSO-$d_6$) (isomer) δ 173.6 (173.6), 172.6, 157.5 (157.5), 144.5 (144.4), 143.9, 143.6, 137.5, 133.5, 131.2 (131.2), 127.9, 126.6 (126.5), 125.9 (125.9), 123.5 (123.3), 119.3, 119.0, 118.0 (117.6), 76.2 (76.1), 70.3 (70.3), 64.8 (64.7), 60.3, 28.22, 21.9 (21.9), 14.6.

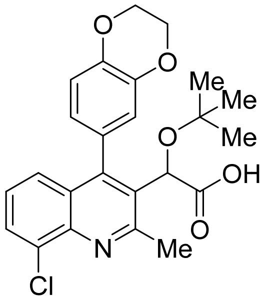

**2-(*Tert*-butoxy)-2-(8-chloro-4-(2,3-dihydrobenzo[*b*][1,4]dioxin-6-yl)-2-methylquinolin-3-yl)acetic acid (19):** To a medium vial, ethyl 2-(*tert*-butoxy)-2-(8-chloro-4-(2,3-dihydrobenzo[*b*][1,4]dioxin-6-yl)-2-methylquinolin-3-yl)acetate (220 mg, 0.469 mmol), NaOH (75 mg, 1.87 mmol) was dissolved in ethanol (2 mL) and $H_2O$ (1 mL). The vial was purged with argon, capped, and refluxed at 80 °C for 16 h. After the reaction was completed, the solution was condensed *in vacuo*. The solid was re-dissolved in water and extracted with ethyl acetate (20 mL). The aqueous layers were acidified to pH 1 and extracted with ethyl acetate (3x30 mL). The combined organic layers were dried with anhydrous sodium sulfate, and condensed *in vacuo* to give a faint yellow solid (165 mg, 80%). $^1$H NMR (500 MHz, DMSO-$d_6$) δ 7.85 – 7.80 (m, 1H), 7.40 – 7.26 (m, 2H), 7.05 (dd, *J* = 17.1, 8.2 Hz, 1H), 6.97 – 6.85 (m, 1H), 6.83 – 6.73 (m, 1H), 5.12 (d, *J* = 6.5 Hz, 1H), 4.38 – 4.20 (m, 4H), 2.74 (d, *J* = 1.3 Hz, 3H), 0.88 (d, *J* = 5.3 Hz, 9H). $^{13}$C NMR (126 MHz, DMSO-$d_6$) (isomer) δ 173.9 (173.9), 160.1 (160.0), 146.3 (146.3), 144.3 (144.2), 143.8, 143.6 (142.5), 132.5 (132.5), 131.6, 129.8, 128.4, 127.9 (127.9), 126.7, 126.3 (126.3), 123.6 (123.5), 119.5 (119.2), 117.9 (117.5), 79.1, 76.0 (75.9), 70.4, 64.8 (64.7), 28.3, 25.3.
